# National hydrologic connectivity classification links wetlands with stream water quality

**DOI:** 10.1038/s44221-023-00057-w

**Published:** 2023-04-06

**Authors:** Scott G. Leibowitz, Ryan A. Hill, Irena F. Creed, Jana E. Compton, Heather E. Golden, Marc H. Weber, Mark C. Rains, Chas E. Jones, E. Henry Lee, Jay R. Christensen, Rebecca A. Bellmore, Charles R. Lane

**Affiliations:** 1 US Environmental Protection Agency (EPA), Center for Public Health and Environmental Assessment (CPHEA), Pacific Ecological Systems Division (PESD), Corvallis, OR, USA; 2 Department of Physical and Environmental Science, University of Toronto, Toronto, Ontario, Canada; 3 US EPA, Center for Environmental Measurement and Modeling (CEMM), Watershed and Ecosystem Characterization Division, Cincinnati, OH, USA; 4 School of Geosciences, University of South Florida, Tampa, FL, USA; 5 ORISE Post-doctoral Participant, c/o US EPA, CPHEA, PESD, Corvallis, OR, USA; 6 National Research Council, c/o US EPA, CPHEA, PESD, Corvallis, OR, USA; 7 US EPA, CEMM, Ecosystem Processes Division, Athens, GA, USA; 8 Present address: Affiliated Tribes of Northwest Indians, Portland, OR, USA; 9 Present address: Southeast Alaska Watershed Coalition, Juneau, AK, USA

## Abstract

Wetland hydrologic connections to downstream waters influence stream water quality. However, no systematic approach for characterizing this connectivity exists. Here using physical principles, we categorized conterminous US freshwater wetlands into four hydrologic connectivity classes based on stream contact and flowpath depth to the nearest stream: riparian, non-riparian shallow, non-riparian mid-depth and non-riparian deep. These classes were heterogeneously distributed over the conterminous United States; for example, riparian dominated the south-eastern and Gulf coasts, while non-riparian deep dominated the Upper Midwest and High Plains. Analysis of a national stream dataset indicated acidification and organic matter brownification increased with connectivity. Eutrophication and sedimentation decreased with wetland area but did not respond to connectivity. This classification advances our mechanistic understanding of wetland influences on water quality nationally and could be applied globally.

Freshwater wetlands (hereafter wetlands) are critical watershed components contributing valuable ecosystem services to society^1^. They attenuate stormflows, augment baseflows and reduce damages from drought and low flows^2–4^. Wetlands support biodiversity, providing habitat for endemic and endangered species, and contribute to wetland–upland ecosystem mosaics occupying inland landscapes^5–7^. However, wetlands are perhaps best known for improving water quality. Within watersheds, wetlands are sources of ecologically beneficial materials (for example, dissolved organic matter) and sinks of detrimental materials, such as excess nutrients (for example, nitrogen and phosphorus), sediments and contaminants, including certain metals^2,3,8–10^. Wetlands are integral to watershed resilience through dampening of hydrological and biogeochemical variability^11^. Despite these ecosystem services, wetlands are vulnerable to degradation or destruction because they are frequently unmapped and poorly protected^12^.

Wetlands alter energy and material transport to downstream waters (for example, streams, rivers, lakes and coastal waters) via their functions and connectivity^13^. Wetlands perform a variety of source, sink, lag, transformation and refuge functions–processes that respectively increase, decrease, affect the timing of (storage and gradual release), alter (change in form) or prevent the loss of (provide suitable habitat to survive adverse conditions) energy, material fluxes or species. The connectivity from wetlands to downstream systems provides the pathways for energy and material transport to downstream waters. In this Article, we use a structural definition of connectivity: ‘the degree to which components of a system are connected and interact through various transport mechanisms’^13^. In contrast, functional definitions of connectivity relate to the frequency, magnitude and duration of actual material flows between system components.

Connectivity occurs along a gradient. At one extreme, hydrologically connected systems facilitate energy or material transport. At the other extreme, hydrologically isolated systems reduce energy or material transport but can increase transformation functions. Connectivity between wetlands and downstream waters influences the structure, function and dynamics of watersheds and broadly contributes to the physical, chemical and biological integrity of those downstream waters^3,10,14,15^. Owing to its effect on the integrity of downstream waters, connectivity can also be an important factor in determining whether wetlands are considered ‘waters of the United States’, that is, federally regulated under the US Clean Water Act^16^. Here, we focus on wetland hydrologic connectivity to downstream waters (hereafter wetland connectivity), since water movement determines fluxes of water quality constituents within watersheds.

As wetlands continue to be drained, filled and destroyed, the need to understand how wetland connectivity mediates water quality has grown^3,9,10,13–16^. Yet there is little research quantifying or classifying wetland connectivity in watersheds, regional wetland landscapes or at national scales. This limits us from addressing crucial questions concerning the role of wetland connectivity in watershed fate and transport processes. For example, a recent conterminous US (CONUS) analysis^8^ suggested that spatially targeting wetland restoration towards nitrogen hotspots would remove more nitrogen than randomly distributed wetland restoration. This analysis did not distinguish between wetland types nor their connections; we argue that wetland connectivity can influence water quality and is integral to understanding the role wetlands play in watersheds. A wetland connectivity classification would help determine whether connectivity influences water quality and should also be considered when prioritizing wetland restoration and protection.

Here, we (1) develop a nationwide wetland connectivity classification that can also be applied at other scales; (2) map and characterize the geospatial distribution of wetland connectivity throughout the CONUS; and (3) use this classification to illustrate the potential effects of wetland connectivity on stream water constituents associated with acidification, excess organic matter-based brownification, eutrophication and sedimentation. This CONUS-wide classification advances our understanding of how wetland connectivity contributes to watershed functions that, in turn, can lead to improved management of waters across the United States and, with increasing data availability, globally.

## Connectivity classification

**Fig. 1 |:**
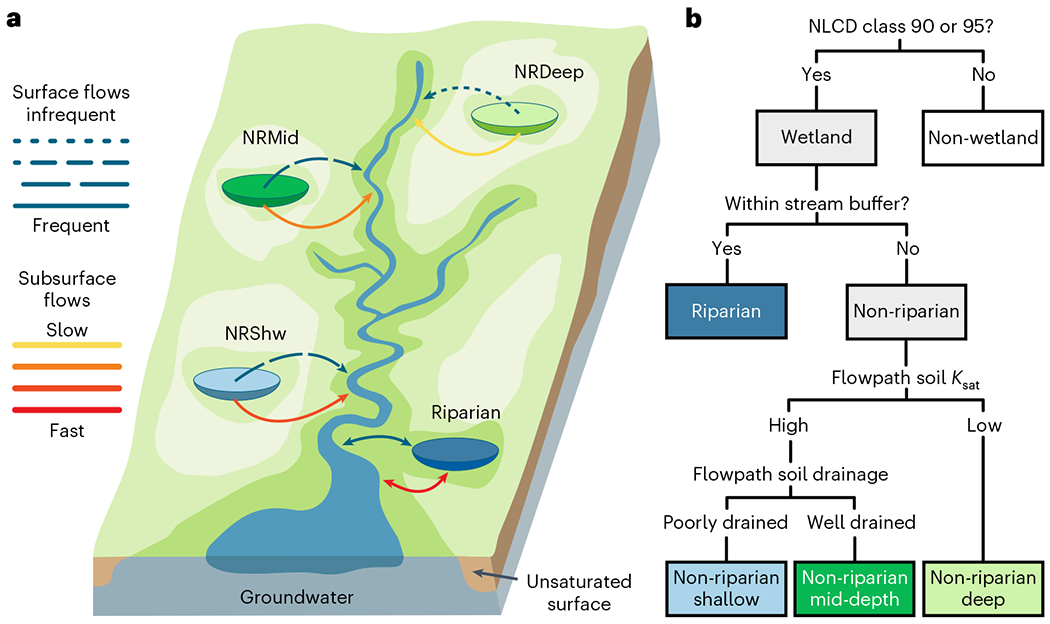
Wetland hydrologic connectivity classification. **a**, Four hydrologic connectivity classes: Riparian wetlands have an outlet within one 30 m pixel from a stream and bidirectional flows. The three non-riparian classes are greater than one pixel from a stream and all have unidirectional flows. NRShw have permeable and poorly drained soils on the flowpath between the wetland and downstream water. Owing to poor drainage, subsurface flows are shallow and surface flows can occur relatively frequently through saturation excess overland flow^17^. NRMid have permeable and well-drained soils on the flowpath. Owing to good drainage, subsurface flows are deeper (mid-depth), but surface flows can occur occasionally through infiltration excess overland flow^17^. NRDeep have impermeable soils on the flowpath. Non-channelized surface flows can occur when the wetland basin is filled with water and additional water input causes the wetland to either spill over or merge into downstream waters^18^, but this is limited to rare and episodic flooding events. Water transport is more common via deep subsurface flowpaths from the bottom of the wetland to downstream waters. Note that depth in the non-riparian class name refers to flowpath and not wetland depth. **b**, Flow chart summarizing classification of wetland hydrologic connectivity classes. Note wetlands are defined on the basis of 2011 NLCD^33^ classes 90 (woody wetland) and 95 (emergent herbaceous wetland); however, woody versus emergent herbaceous type is not incorporated into the resulting classification. For details, see Methods.

Our classification uses CONUS-wide geospatial data of stream networks, wetlands and flowpath characteristics to define four wetland classes on the basis of their hydrologic connectivity to downstream waters (Fig. 1, Table 1 and Methods); note that depth in the class name refers to flowpath and not wetland depth:

1. Riparian wetlands (Riparian) adjoin rivers and streams, have very frequent bidirectional connections to them, and provide unidirectional transport from upgradient hillslopes.
2. Non-riparian shallow wetlands (NRShw) have permeable and poorly drained soils on flowpaths between wetlands and downstream waters. Owing to poor drainage, subsurface flows are shallow and relatively infrequent surface flows can occur through saturation excess overland flow (that is, when soil water holding capacity is full)^17^.
3. Non-riparian mid-depth wetlands (NRMid) have permeable and well-drained soils on flowpaths between wetlands and downstream waters. Good drainage allows deeper, mid-depth subsurface flows, but surface flows can occur occasionally through infiltration excess overland flow (that is, when rainfall intensity exceeds soil infiltration)^17^.
4. Non-riparian deep wetlands (NRDeep) have impermeable soils on flowpaths between wetlands and downstream waters. Non-channelized surface flows can occur when the wetland basin is filled with water and spills over^17,18^, but this is limited to rare and episodic flooding events. Water transport via deep subsurface flowpaths from the bottom of the wetland to downstream waters is more common.

We hypothesized that Riparian connectivity is the highest, since these wetlands have frequent, short-distance, surface and subsurface hydrologic connections to streams. In contrast, we hypothesized that non-riparian wetland connectivity decreases with increasing depth of the subsurface flowpath, assuming non-riparian connectivity is dominated by subsurface hydrologic connections to the stream. Consequently, we rank wetland connectivity as Riparian > NRShw > NRMid > NRDeep. Note that our classification does not consider distances between wetlands and downstream waters, other than determining whether a wetland adjoins a stream (Methods). Nor does it account for travel times between wetlands and downstream waters. Rather, it qualitatively represents how quickly water would be expected to travel through flowpaths of similar lengths.

To validate our classification, we qualitatively estimated the expected magnitudes of the wetland connectivity classes and compared these with quantitative results on the areal distributions of these classes for six case study regions (Methods, Supplementary Information, Supplementary Fig. 1 and Supplementary Table 1). We overpredicted wetland area in three cases and underpredicted area in one. Overall, there was good concurrence between the expected magnitudes of wetland connectivity classes and quantitative results.

## Distribution of connectivity classes

Riparian was the dominant connectivity class based on total wetland number, total wetland area and per cent of CONUS area (Table 2). NRShw had the smallest total wetland number and among the smallest total wetland area. NRMid and NRDeep had similar wetland numbers, but NRMid had about 1.2 times the total wetland area compared with NRDeep. Riparian was made up 3.8% of the CONUS land area, compared with about 0.5% each for the three non-riparian classes. The mean area of 0.11 km^2^ per Riparian wetland was 1.8, 3.7 and 5.5 times larger than mean areas of NRShw, NRMid and NRDeep, respectively.

**Fig. 2 |:**
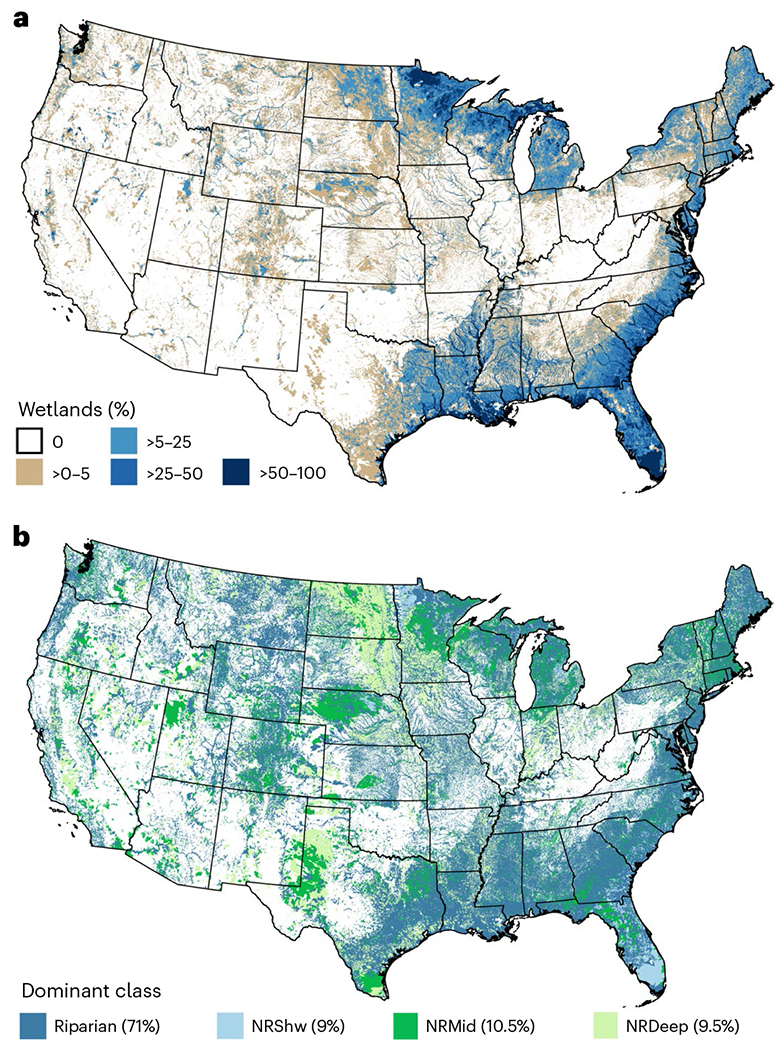
Wetland characteristics of stream catchments across the CONUS. **a**, Wetlands as a per cent of total land cover within the NHDPlusV2 catchment. **b**, Dominant wetland hydrologic connectivity class within NHDPlusV2 catchments (parenthetical values in key indicate per cent of catchments across the CONUS dominated by that particular connectivity class).

Wetlands are unevenly distributed across the CONUS (Fig. 2a) and by connectivity classes (Fig. 2b and Supplementary Figs. 2 and 3). Areas with the highest wetland occurrence (>50%) were parts of the South-eastern United States, a narrow band along the Atlantic and Gulf coasts, and areas in northern Minnesota and the upper peninsula of Michigan. These locations grade into more extensive areas with >25% wetlands. Among wetland connectivity classes, riparian dominated throughout much of the CONUS. NRShw was most widespread in only a few areas, including portions of Florida and the Atlantic coast, as well as parts of Minnesota, Nebraska and the lower peninsula of Michigan. NRMid dominated in western Texas and eastern New Mexico, the southern tip of Texas, around the Great Salt Lake in Utah, and parts of Minnesota and Nebraska. NRDeep was most prevalent in the eastern Dakotas, western Minnesota, the Texas panhandle and the southern portion of California’s Central Valley.

## Wetland connectivity and stream water quality

To assess the influence of wetland connectivity classes on downstream water quality, we developed a set of linear mixed effects regression models for 11 in-stream water quality constituents (Supplementary Table 2) that were placed into four functional groups (acidification, brownification, eutrophication and sedimentation; Methods). Although the precision of these models (Supplementary Table 3) was similar to those for models with all wetlands combined (Supplementary Table 4), the purpose of these models was not to produce better predictions of water quality. Rather, our purpose was to decompose and test the individual effects of the four wetland classes on water quality.

**Fig. 3 |:**
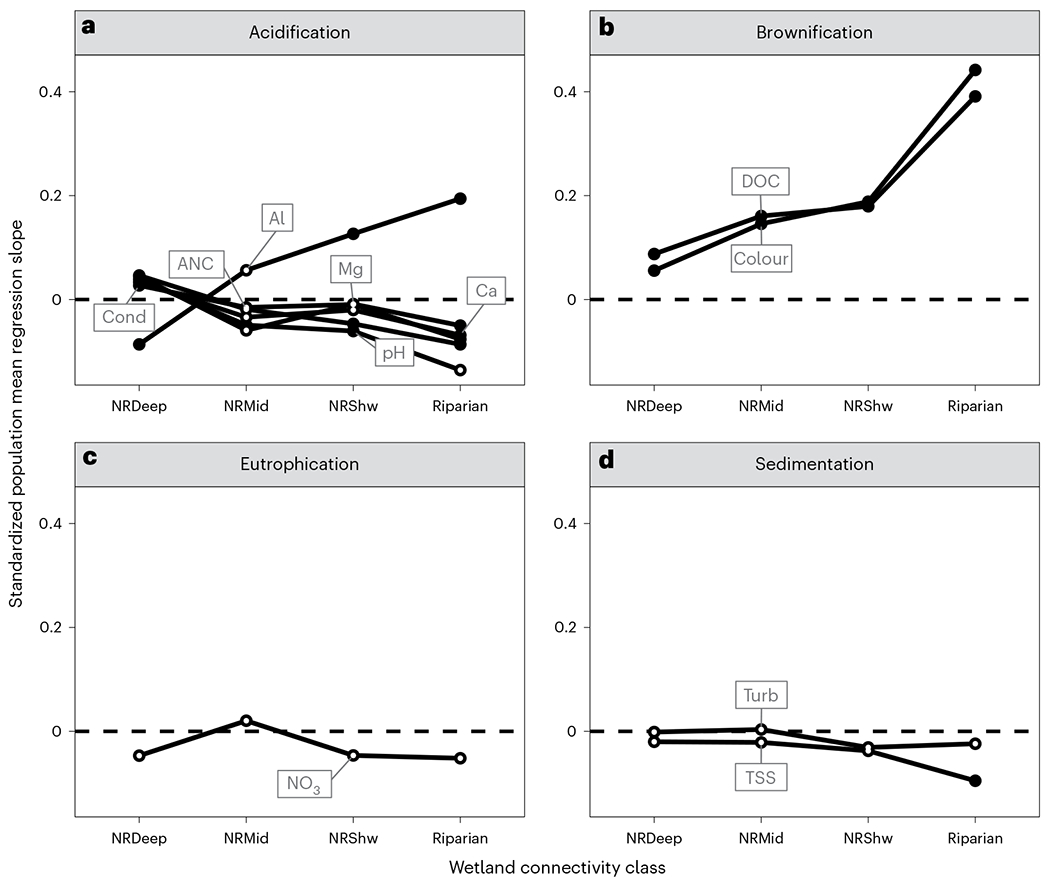
Relationships between four groups of stream constituents and wetland hydrologic connectivity based on standardized population mean regression slopes from linear mixed effects models. **a**–**d**, Mean slope represents the standardized relationship between the constituent and wetland connectivity class averaged across all regions. A filled (dark) circle indicates that the regression slope is significantly different than zero, that is, the regression slope plus or minus the confidence interval (two times the standard error of the slope estimate) does not overlap with zero; points represented by an open (light) circle are not significantly different than zero (Supplementary Table 3). Wetland connectivity for the four classes is NRDeep < NRMid < NRShw < Riparian. For details on the modelling approach, see Methods. Dashed line represents zero intercept. Acidification (**a**): cond (*n* = 1,764), Ca (*n* = 1,787), Mg (*n* = 1,787), Al (*n* = 1,180), pH (*n* = 1,764) and ANC (*n* = 1,788). Brownification (**b**): DOC (*n* = 1,788) and colour (*n* = 1,786). Eutrophication (**c**): NO_3_ (*n* = 1,338). Sedimentation (**d**): turb (*n* = 1,764) and TSS (*n* = 1,694).

The acidification constituents were related to wetland connectivity classes, reflecting the role of residence time on acidic and basic water conditions^19^ (Fig. 3a). Al had a negative standardized population mean regression slope (hereafter mean slope)–that is, an inverse relationship–with the lowest level of wetland connectivity (NRDeep), but the mean slopes switched signs and became increasingly positive with the increasing presence of wetlands with higher connectivity. Al, which is typically mobilized in acidic soils and soil waters^20^, increased with increasing wetland connectivity through an acidifying organic soil matrix. In contrast, Mg, Ca, acid neutralizing capacity (ANC) and specific conductance (cond) had a positive mean slope with the lowest level of wetland connectivity (NRDeep), and the mean slopes switched signs and became increasingly negative with increasing wetland connectivity (Fig. 3a). Mg, Ca, ANC and cond are typically mobilized via bedrock weathering^21^, with greater mobilization associated with longer residence times^19^ and lower acidification^22^. The negative mean slope between NRDeep and Al, together with the positive mean slope between NRDeep and Mg, Ca, ANC and cond, may reflect how water quality constituents are influenced by the longer water residence time and potentially greater bedrock weathering reaction potential along the deep pathway of wetland connections to streams^23^. However, the model fixed effects explained a low proportion of the variance in Al (Supplementary Table 3), and relationships should be interpreted with caution.

The brownification constituents, colour and dissolved organic carbon (DOC), were positively related to greater presence of each wetland connectivity class but had higher mean slopes in the presence of increased wetland connectivity (Fig. 3b). Higher browning as a function of increased wetland connectivity through shallow subsurface soils reflects the high DOC production potential of wetlands and the interaction with carbon-rich organic layers as water flows from the wetland to the stream. The low mean slope between NRDeep and browning further underscores this finding, as adsorption of DOC to sediments^24^ and microbial decomposition of DOC^25^ remove carbon along the deep flowpath.

Comparing mean slopes between classes, NRDeep and NRShw were significantly different for all acidification and brownification constituents (Supplementary Table 5). NRDeep and NRMid mean slopes were significantly different for most acidification and brownification constituents; the lack of significance for Ca and Mg could mean that these effects are weaker or that these constituents are not as sensitive for these classes. None were significantly different between NRMid and NRShw wetlands except Al. These similar behaviours could mean that the STATSGO2 soil drainage class is not the proper attribute to distinguish between NRMid and NRShw, or NRMid and NRShw do not represent two distinct classes.

While wetlands are considered important sites for denitrification and nitrate removal^3,8–10^ as well as sediment filtration^26^, these capacities did not vary with wetland connectivity class. Mean slopes of NO_3_, turbidity (turb) and total suspended solids (TSS) were not significantly different from zero for any connectivity class, except for a significant negative TSS mean slope for Riparian (Fig. 3c,d and Supplementary Table 3). Although NO_3_ and TSS were not related to wetland connectivity class, they both had significant negative mean slopes when all wetlands were combined into a single watershed measure (Supplementary Table 4 and Methods). This reaffirms the importance of wetland presence–but not connectivity–to the functions of nitrate and sediment removal^10^. As with Al, model fixed effects explained a low proportion of the variance in turb and TSS and should be interpreted with caution.

Riparian flowpath linkages to water quality were strong (Fig. 3 and Supplementary Table 3). These flowpaths are dominated by carbon-rich organic soils with relatively few base cations and lower pH, and they are expected to have short residence times and lower adsorption and weathering potential. Thus, streams with higher Riparian connections tended to have higher DOC and Al, and lower base cations. Riparian generally had a stronger filtering effect than other classes, as suggested by the negative TSS slope for Riparian. This may be because of the runoff capturing capacity of Riparian wetlands and their short connections to streams. Rapid DOC transport from its source can occur when there is limited residence time for biogeochemical transformations, and if hydrological transport mechanisms are frequently available^27^.

## Discussion and conclusions

Restoring and protecting wetlands has been important for managing water quality for decades^28^. This includes spatial targeting of wetland restoration or protection, which can improve water quality better than non-targeted efforts. For example, targeting restoration in nitrogen hotspots, where nutrient sources are abundant, is probably more effective than restoring wetlands randomly or in non-agricultural areas^8^. Our wetland connectivity classification provides a critical dimension for improving landscape-scale targeting for water quality management.

The strong relationship between wetland connectivity and acidification (Al and basic constituents) and brownification (colour and DOC) suggests watershed managers should consider wetland connectivity class for restoration or protection of stream water quality related to these constituent groups. For example, restoring NRDeep wetlands would produce less colour and DOC, if these were of concern, than the same increase in Riparian wetlands, since NRDeep has the smaller mean slope (Fig. 3). Targeting could be aided by maps showing the distribution of individual wetland classes (Supplementary Fig. 2), number of classes present (Supplementary Fig. 3) and dominant wetland class (Fig. 2b). For instance, in locations where wetlands are at risk and stream acidification is a threat, these maps could be used to identify NRDeep wetlands for protection or restoration, since NRDeep has a negative relationship with Al and a positive relationship with basic constituents^29,30^ (Fig. 3).

In contrast, watershed managers should spatially target restoration or protection of wetlands, regardless of connectivity, to improve water quality related to NO_3_ removal^8^ or filtration of TSS or turb. To maximize these functions, our analysis of combined wetlands (Supplementary Table 4) suggests that areas of restored or protected wetlands of any class should be maximized. In all such cases, both positive and negative effects of increasing a constituent need to be balanced, for example, increasing DOC concentrations may elevate methylmercury or drinking water disinfectant byproducts.

This national wetland connectivity classification system provides researchers and resource managers insight on a primary mechanism by which wetlands affect downstream water quality: wetland-to-stream hydrologic connectivity. The system affords improved methods for spatially targeting wetland restoration and protection. Our finding that 8 of 11 individual constituents responded to differences in connectivity supports incorporating wetland connectivity into watershed management decisions for constituents or constituent groups not yet researched. Our results also underscore that losing the ‘portfolio’–or full array–of wetland connectivity could cause negative impacts on downstream waters^14^. Creed et al. proposed maintaining this complete portfolio of wetland connectivity as an approach for watershed management^12^. Our classification could also prove useful in determining whether particular wetlands are considered waters of the United States under the US Clean Water Act^16^; this will depend on the standards for identifying waters of the United States established by regulation (86 FR 69372) or the US Supreme Court.

### Limitations and future developments

Our classification is a discrete characterization of continuous patterns and so is an approximation. For example, the Central Florida Everglades consists of a ridge–slough mosaic that might be considered riparian, but the National Hydrography Dataset Plus Version 2 (NHDPlusV2) indicated a few rivers. Consequently, the region was classified mostly as NRShw, indicative of the area’s shallow water table.

Our modelling approach demonstrates correlative relationships, a foundation of most statistical models. We supported our findings using (1) independent variables representing underlying hydrological processes driving wetland connectivity to streams and (2) foundational literature corroborating potential physically based explanations that underly our models’ statistical relationships. We further underscore that we have made associations between wetland connectivity and water quality across the entire CONUS.

Several developments would improve this approach. (1) Because our results are based on empirical models and correlation, our findings need to be validated with process-based approaches that can support causal mechanisms linking wetland connectivity to downstream water quality. Field studies are especially needed to investigate NRMid and NRShw wetlands, their effects on water quality, alternative geospatial indicators and whether these wetlands represent separate classes. (2) Distance or travel times between wetlands and downstream waters could be incorporated into the approach, although this would increase the complexity of the analysis and might not lead to a meaningful improvement. However, it might also serve as a metric of sensitivity to climate change as residence times and/or infrastructure can change with drought and floods. (3) Hydrologic alterations, such as canals, tile drains and drainage ditches, can affect wetland connectivity, so our method could include new information on these factors as it becomes available, for example, agricultural tile drainage data^31^. (4) Our classification system could incorporate smaller wetlands (<900 m^2^) by using higher-resolution datasets, such as Lidar-based depressional analyses^32^. In fact, the classification system could be applied to individual wetlands of any size by evaluating whether they adjoin a stream and the flowpath soil properties using field methods. The classification system is flexible and could be applied at multiple scales using various data sources. However, smaller wetlands, which are important biogeochemical reactors^10^, will not be captured at coarser scales, thereby influencing observed wetland–water quality relations. (5) Our classification approach could be expanded using international datasets, for example, WorldCover land cover data (https://esa-worldcover.org/en) and SoilGrids Global Soil Data (https://www.isric.org/explore/soilgrids, which includes soil properties that are empirically related to *K*_sat_). We are not aware of global-scale soil drainage class maps, but it is available at specific locations, for example, Africa SoilGrids drainage classes (https://data.isric.org/geonetwork/srv/api/records/953d0964-6746-489a-a8d1-f188595516a9). Our existing classification system could be implemented at international locations having complete data, or NRShw and NRMid could be combined where drainage class data are unavailable.

Our national-scale wetland connectivity classification system provides a critical link to improving water quality beyond individual wetland restoration alone. Elucidating the ‘black box’ between wetlands and water quality responses provides improved information for watershed-scale water quality management. Through our analysis, we demonstrated strong relationships between our wetland connectivity classes and most stream water quality constituents. Until now, no standardized approach existed to characterize hydrologic connectivity between wetlands and downstream waters at the CONUS scale. Consequently, determining regional and nationwide effects of wetland connectivity on endpoints such as water quality was impossible. Our systematic approach to quantifying the distribution of the wetland connectivity classes across the CONUS using hydrologic principles and geospatial analyses provides important insights on how wetlands affect stream water quality at the CONUS scale. The flexibility of the classification approach in handling diverse data can make it useful at different scales and across the globe.

## Methods

### Wetland dataset

#### Wetlands and streams.

We used the publicly available, 2011 National Land Cover Dataset (NLCD)^33^ and NHDPlusV2 (ref. 34) to create the hydrologic framework of wetlands and downstream waters. We used NLCD, rather than the National Wetlands Inventory (https://www.fws.gov/wetlands/), because it has a more consistent methodology than the National Wetlands Inventory across the United States and controls better for individual year and density, which affect our assessment. A disadvantage of NLCD is that the 900 m^2^ pixel was not sufficient for detecting small wetlands, which are important for water quality^10,12,14^. While small wetlands are numerous and can be regionally important, NLCD is appropriate for a CONUS-scale analysis. Our use of NLCD from a single year means that we cannot indicate change from wetlands to other land covers or vice versa; we can only examine extant wetland area. Wetlands, both inland and coastal, were identified using the woody (class 90) and emergent herbaceous (class 95) NLCD classes combined. However, class 90 versus class 95 were not separately incorporated into the resulting classification. Sets of adjoining wetland pixels that were completely surrounded by non-wetland pixels were grouped into contiguous wetland patches within the landscape and assigned a wetland identification number (WetId).

We used NHDPlusV2 as the hydrologic framework upon which we characterized wetland connectivity. NHDPlusV2 is a value-added version of the National Hydrography Dataset (NHD). The NHD depicts the stream network of the CONUS using 1:100,000 scale blue lines (for example, rivers and streams) of US Geological Survey topographic quadrangle maps. To make the NHDPlusV2, these blue lines were linked to 30 m digital elevation models and major river basin boundaries to derive local catchment boundaries for each stream segment, that is, the local area draining to a stream segment excluding upstream contributions. This process created 2.6 million catchments that are each tied to a respective stream segment. Just as the NLCD omits small wetlands, the 1:100,000 scale NHDPlusV2 excludes small streams. As a result, it underestimates total stream length and, in combination with the omission of small wetlands, reduces the number of observed wetland–stream connections^35^.

Contiguous wetland patches identified in the earlier step were subdivided when their areas crossed NHDPlusV2 hydrologic catchment boundaries. Subdividing wetlands among NHDPlusV2 catchments created hydrologically distinct wetlands from contiguous patches and a wetland framework that worked within the existing hydrologic framework of streams and local catchments of the NHDPlusV2. The subdivision of contiguous wetlands among hydrologic boundaries produced about 6.7 million unique wetland units (Table 2). This nested framework allowed us to calculate wetland-specific characteristics and to hydrologically aggregate these wetland characteristics to the 2.6 million receiving stream segments and their associated watersheds of the NHDPlusV2.

Characterizing the connectivity of wetland units to receiving waters required a geospatial representation of streams. We identified receiving waters with a combination of NLCD water pixels and a raster representation of stream lines extracted from the NHDPlusV2. The combination of these datasets (hereafter buffered streams) provided width for rivers that were large enough to be detected with Landsat imagery. For streams and rivers too narrow to be detected with Landsat imagery, a raster version of the NHDPlusV2 stream lines provided an estimate of stream locations. Note that while most of the NLCD water pixels represented rivers or streams, they could also represent lakes or estuaries.

Geospatial processing was performed with scripts in the Python programming language^36^, using the NumPy module^37^ and ESRI Arc-GIS^38^ tools. Other processing steps were done in the R programming language^39^.

#### Flowpaths.

Flowpaths from wetland outlets (pour points) to streams were necessary to conduct the connectivity classification. A secondary product of the NHDPlusV2 is a set of hydrologically corrected digital elevation models and rasters depicting flow (both direction and accumulation) across landscapes at a 30 m resolution^34^. We used these hydrologic rasters to define flowpaths connecting streams and wetlands. Within each wetland, we first defined the wetland outlet as the pixel with the highest flow accumulation value. We then used the ArcGIS Cost Path tool^38^ to delineate flowpaths from wetland outlets to the edge of receiving streams; we assumed that these surficial flowpaths also represented subsurface flowpaths. In some cases, NHDPlusV2 catchments do not contain a stream segment. In these catchments, wetland flowpaths extended into downstream catchments until they encountered a stream segment. Flowpaths that passed through multiple wetlands were topologically connected on the basis of hydrologic rasters that depict the pixel-to-pixel flow directions across land surfaces^40^.

### Wetland hydrologic connectivity classification

Several methods have been used to quantify wetland connectivity, at wetland to watershed scales (Table 2 in the US Environmental Protection Agency (EPA) ref. 16). Yet approaches for characterizing connectivity at large, national scales do not exist. Our classification system was inspired by the conceptual model of Cohen et al.^14^ Like all classification systems, ours prioritizes certain attributes over others. Specifically, we emphasize landscape attributes that (1) identify a gradient of high to low hydrologic connectivity on the basis of hydrologic principles, (2) qualitatively indicate how quickly water travels through unit flowpaths (our approach does not account for travel times between wetlands and downstream waters), and (3) are available as CONUS-wide datasets. Thus, factors that would be critical to travel time, such as slope and distance, were not included in our classification. Further, we define connectivity structurally^13^, rather than functionally, which represents hydrologic connectivity given sufficient surface water and/or groundwater availability. This removes the necessity for dynamic attributes such as climate-based indicators.

Given the above, we emphasized datasets that are used to determine whether a wetland directly adjoins a stream and, if not, the soil-based attributes that influence the flowpaths that water takes from the wetland to the downstream water. If a wetland outlet (pour point) was positioned within one 30 m pixel (ordinal and cardinal directions) from a buffered stream, it was classified as riparian (Fig. 1). In contrast, a non-riparian wetland had an outlet more than one 30 m pixel from a buffered stream.

Non-riparian wetlands were further distinguished on the basis of the soil characteristics of their flowpaths, specifically *K*_sat_ and soil drainage class, which were both based on STATSGO2 data (https://www.nrcs.usda.gov/resources/data-and-reports/description-of-statsgo2-database). For that effort, we used a *K*_sat_ cut-off value of 5.08 cm h^−1^ to distinguish between permeable and impermeable soils, that is, the threshold between very fine sandy loams and sandy loams (C. Johnson, USDA-NRCS Soil Survey, personal communication). In cases where more than one *K*_sat_ value occurred over the flowpath, the minimum value was used, with the assumption that the smaller value would be limiting to hydrologic flow (that is, lead to longer travel times). We defined poorly drained soils as consisting of somewhat poorly drained, poorly drained and very poorly drained NRCS classifications, and well-drained soils consisted of excessively drained, somewhat excessively drained, well drained and moderately well drained, NRCS classes^41^. If more than one drainage class value occurred over the length of the flowpath, the drainage class that occurred over the greatest length of the path was used. Our assumption was that drainage class was not limiting like *K*_sat_, because the latter was used to distinguish between surface and subsurface flows, while the former was used to represent differences between subsurface flows and so should be based on the most common occurrence.

NRShw have permeable (*K*_sat_ > 5.08 cm h^−1^) and poorly drained soils on the flowpath between the wetland and downstream water. Owing to poor drainage, subsurface flows are shallow and surface flows can occur relatively frequently through saturation excess overland flow^17^. NRMid have permeable (*K*_sat_ > 5.08 cm h^−1^) and well-drained soils on the flowpath between the wetland and downstream water. Owing to good drainage, subsurface flows are deeper (mid-depth), but surface flows can occur occasionally through infiltration excess overland flow^17^. NRDeep have impermeable soils (*K*_sat_ < 5.08 cm h^−1^) on the flowpath between the wetland and downstream water. Non-channelized surface flows can occur when the wetland basin is filled with water and additional water inputs cause the wetland to either spill over or merge into downstream waters^17,18^, but this is limited to rare and episodic flooding events. Instead, water transport is more commonly via deep subsurface flowpaths from the bottom of the wetland to downstream waters. Our four classes are similar to the ideas presented in the Cohen et al.^14^ conceptual model, but they add additional information. Further, these four classes logically resulted from the application of the riparian, *K*_sat_ and drainage data.

Since our classification of connectivity is structural, it was not possible to validate it by comparing our results with those from a functional approach such as Vanderhoof and colleagues, who examined changes in surface water connectivity as a function of climate variation^42^. To validate our approach, we conducted a qualitative validation assessment in six case study regions (Supplementary Fig. 1): California vernal pools, Louisiana bottomland hardwoods and swamps, playa lakes, pocosins and Carolina bays, prairie potholes and Southern Florida (although each region, except Southern Florida, is named for iconic wetlands, other wetlands occur within the regions and were incorporated into the analysis). Wetland area within these regions ranged from 621 km^2^ for the playa lakes region to 42,169 km^2^ for the pocosins and Carolina bays region (Supplementary Table 1). For each of these regions, we assembled the following information (Supplementary Information): (1) Background on each wetland type (for example, prairie potholes), including climatic, geologic and/or topographic controls. (2) The expected magnitude of the four wetland classes in each region. Expected magnitude was a categorical assessment (high, medium or low) of the relative area of wetlands on the basis of a qualitative evaluation of the factors affecting wetland connectivity in each region. We determined this magnitude on the basis of a combination of the literature, first principals, our own familiarity with these regions and, in the case of the playa lakes region, in consultation with a regional expert. (3) Evaluation of the expected magnitude values compared with the actual areal distributions of the four connectivity classes resulting from our analysis. For this evaluation, we considered the low, medium and high categories to comprise 0–10%, 11–50% and 51–100% wetland area, respectively.

Supplementary Figs. 4–9 show the wetland hydrologic connectivity classes in six different regional wetland landscapes. These figures illustrate several elements of the wetland dataset, including the connectivity classes, the NHDPlusV2 streams and NLCD water that combine to make the buffered streams, and the flowpaths between wetlands and downstream waters. A CONUS-wide dataset containing this information is available. For each of 59 hydrologic subregions of the CONUS, the dataset consists of three components: (1) A raster that contains NHDPlusV2 streams, NLCD water pixels and the flowpaths that connect wetlands and downstream waters. (2) A wetland raster that contains the WetId of each wetland, where each WetId corresponds to a single stand-alone wetland pixel or a group of adjoining wetland pixels. Note that WetIds are not globally unique, but are unique within each of the 59 subunits of NHDPlusV2 Hydrologic Regions (that is, raster processing units). Within these raster processing units, each WetId contains only a single pour point. (3) A table of wetland characteristics indexed by WetId, including wetland area, and wetland hydrologic connectivity class. These three components of the wetland dataset and details on their use are available by NHDPlusV2 Hydrologic Region at https://doi.org/10.23719/1528587 ref. 43.

### Empirical assessment

To explore the role of wetland connectivity on downstream water quality, we tested hypotheses that structural characteristics of wetland connectivity influence downstream biogeochemistry (Table 1). Riparian wetlands have a high degree of connectivity and convey water to downstream waters quickly, with minor processing relative to other wetland connectivity types^27^. Non-riparian wetlands have a lower degree of connectivity, convey water into surface waters via subsurface flowpaths more slowly and the water is altered during the longer residence times in the geologic deposits it traverses along the way^19^. We hypothesized Riparian connections would be brief and frequent, passing primarily through organic-rich surface soils, with less potential for adsorption–desorption processes or weathering in mineral soil. This could result in relatively high concentrations of DOC and low concentrations of base cations. In contrast, we hypothesized NRDeep connections would pass slowly through deeper mineral soil and bedrock, with more potential for weathering release of base cations along the flowpath. NRShw and NRMid should fall between Riparian and NRDeep. We tested these hypotheses of coupled hydrologic connectivity–biogeochemical behaviours against measured stream water quality data (‘Wetland connectivity and stream water quality’).

We focused on 11 water quality constituents that are known to affect stream ecosystem functions and associated services^44^ and are often sampled in stream assessments (Supplementary Table 2). These constituents were taken from distinct samples once during summer low flows at 1,788 sites as part of the US EPA’s 2008/09 National Rivers and Streams Assessment (NRSA). NRSA is a national survey that uses a spatially balanced, probabilistic sampling design^45,46^ and represents a spatially extensive set of synoptic water quality samples across the CONUS. The strength of the NRSA water quality data are in their spatial extent across the CONUS. The samples are taken during summer to capture water quality constituent concentrations under baseflow conditions. Since the samples are meant to represent spatial variability, only one sample is taken at each site (except 10% of sites were revisited for quality assurance purposes^45^), rendering before and after data unavailable–publicly or otherwise–across sites. Concomitant national data on soil solution chemistry are also not available.

These 11 constituents were placed in one of four functional groups reflecting processes known to affect stream ecosystem productivity, food web energy transfer and biodiversity^44^: (1) acidification: cond, Ca, Mg, Al, pH and ANC; (b) brownification^47,48^: DOC and colour; (c) eutrophication: NO_3_ and (d) sedimentation: turb and TSS. To investigate the effects of wetland connectivity on these constituents, we used linear mixed effects models to determine the influence of the four wetland connectivity classes on the 11 stream water quality constituents (Supplementary Table 2). We then examined the standardized population mean regression slopes (hereafter mean slopes) that represent the standardized relationship between the constituent and wetland connectivity type averaged across CONUS regions (Supplementary Table 3). We chose linear models to test the importance of wetland connectivity on water quality because (1) the test for the connectivity main effect on water quality is equivalent to the one-way analysis of covariance when each watershed is composed of a single connectivity class, (2) residual analysis did not indicate the assumption of a linear response was violated; and (3) generalized additive models do not allow for random slopes and also would have complicated the comparison of effect sizes among wetland classes and constituent types.

In each model, we included watershed-level summaries of the four wetland connectivity classes as predictors of in-stream water quality to assess the effect of wetland connectivity on downstream waters. Wetland connectivity classes were quantified within watersheds as the per cent of watershed area composed of each class. Thus, each wetland connectivity class was summarized as a continuous measurement (0–100%). Critically, the percentages of the four connectivity classes included the entire watershed area and would not sum to 100% on their own unless a watershed was comprised entirely of wetlands, which never occurred. Quantifying the wetland connectivity classes as percentages of the watershed allowed us to include all four classes as predictors for each water sample in all models and to test the importance of wetland connectivity on downstream water quality. The omission of small wetlands from the NLCD dataset would only be expected to impact model results in areas where these small wetlands make up a substantial proportion of total wetland area.

In the simple case where each watershed is composed of a single connectivity class, the basic linear model is equivalent to the analysis of variance (ANOVA) model with connectivity class as a qualitative factor having four levels. The *F*-test for the connectivity main effect is used to test whether the water quality is different between the four connectivity classes. As an extension of the ANOVA model to the situation where each watershed is composed of one or more connectivity classes, the 0–1 indicator variables for each connectivity class are replaced by the per cent of watershed area composed of each class. We further extend the basic model by selecting as covariates a unique set of additional predictor variables (Supplementary Table 2), on the basis of previous work^49^ or our professional expertize for each constituent of interest; this was done to minimize the chance of excluded variable bias in our models. The alternate models always included the four wetland connectivity class variables to test the connectivity main effect on water quality, analogous to a one-way ANOVA with covariates. These additional variables included one in-stream measurement of median sediment substrate size collected at the time of NRSA sampling as well as several watershed landscape variables from the StreamCat dataset^50^. Note that there was no covariate on wetland quality, due to the lack of such a national dataset. Although this could also affect water quality, we felt that this was not a major issue since the covariates in the model included factors that would affect wetland quality (Supplementary Table 2); for example, mean imperviousness of anthropogenic surfaces, point source N and per cent of watershed area classified as crop and hay land use. Further, this was a national-scale analysis with almost 7 million wetlands, and such issues should balance out at that scale and sampling magnitude.

All response variables were natural log transformed, except for pH which was raised to the power of four, to meet assumptions of normality of residual errors. Selected covariates (Supplementary Table 2) were also natural log transformed to achieve normality. Plots of residual errors versus fitted values identified diagonal stripes in TSS, Al and NO_3_, indicating inflation due to zeros or detection limits (censored values). Although techniques exist to model censored or zero-inflated data, we chose to remove these observations for simplicity and to use the same statistical methods across all constituent types. We visually confirmed that removal of these sites did not substantially alter or bias the distribution of samples across the United States. Doing so resulted in 1,694, 1,180 and 1,338 out of 1,788 possible observations to model TSS, Al and NO_3_, respectively. The remaining models were constructed with 1,764–1,788 samples depending on the number of valid measurements achieved for each constituent and available watershed metrics for sampled sites (Supplementary Table 3). In addition to transformations, response and predictor variables were standardized to have a mean of zero and standard deviation (s.d.) of one. Standardizing both response and predictor variables allowed for comparison of regression slopes across models and was necessary because the ranges of both response and predictor variables varied by several orders of magnitude (that is, pH had a range of one order of magnitude while at the opposite extreme, turb had a range of seven orders of magnitude). Standardized slopes of this form can be interpreted as the proportional change in the s.d. of the response variable for a one s.d. change in the predictor variable. For example, a regression parameter of 0.5 means that the response variable changed by one-half s.d. in response to a one s.d. change in the associated predictor variable. Finally, multicollinearity among predictors was tested at the time of modelling with variable inflation factors^51^. In all cases, variable inflation factors were below two, including those for wetlands, indicating low correlations among predictors.

Across large, physically complex regions, such as the CONUS, geophysical settings and processes that control relationships between landscapes and downstream waters can vary substantially. This geophysical variability is often reflected statistically as non-independence of sites within regions and as cross-regional variability in relationships between response and predictor variables (that is, slopes). In such cases, standard linear regression models may not be sufficient to characterize these complex relationships and mixed effects models are needed. Mixed effects modelling can account for variation in the response–predictor variable relationships by fitting random intercepts and slopes with respect to a grouping variable, such as eco-region or physiographic region^52^. This approach allows model parameters (intercepts, slopes or both) for certain factors to vary stochastically by region and come from some assumed distribution (that is, Gaussian) if needed. If a predictor variable has a consistent relationship with the response variable across regions, it can be modelled as a fixed effect without a random effect. A model that uses both fixed and random effects is called a mixed effects model. As a grouping variable, we tested the nine ecoregions of the US EPA National Aquatic Resource Surveys^45^ and 24 US Geological Survey Physiographic Divisions and Provinces (https://water.usgs.gov/GIS/metadata/usgswrd/XML/physio.xml). When assessed with Akaike’s Information Criterion^53^, Physiographic Provinces were the best grouping variable in all models, except for NO_3_ which was best modelled with the nine National Aquatic Resource Surveys ecoregions.

To develop the models, we began by fitting the most complex model possible that included both fixed and random coefficients for each covariate and the intercept with respect to the grouping variable (R package::function lme4::lmer^54^). We then conducted backward selection on the random effects followed by the fixed effects, while forcing the model to retain the fixed effects of the four wetland classes (R package::function lmerTest::step^55^). Forcing the model to retain fixed effects allowed us to extract parameter estimates for plotting even when they were not significantly different from zero. This process uses a likelihood ratio test to remove fixed and random effects with mean and/or variance, respectively, not significantly different from zero at the 0.05 level of significance^56^. The most parsimonious model was chosen by backwards hierarchical elimination of non-significant terms. Unlike model selection for choosing the set of predictor variables, a backward–forward model selection process is not recommended for the inclusion or exclusion of a random slope term to achieve a more parsimonious model. Indeed, Kuznetsova et al.^55^ suggest that backwards selection of mixed effects models, as used here, avoids the risk of applying a model that is too simple and that could produce excluded variable bias. We then extracted the standardized parameter estimates and standard errors for plotting and model interpretation (Fig. 3). When a fixed effect also has a random component in a mixed effects model, the slope of the fixed effect is the population mean of random slopes across the grouping variable. In some models, the selection procedure determined that random components of wetland measures were not needed. In those cases, the parameters of the fixed effects alone were extracted and used. All analyses were conducted in the R statistical language^39^.

### Reporting summary

Further information on research design is available in the Nature Portfolio Reporting Summary linked to this article.

## Data availability

All datasets are available at the US EPA’s ScienceHub website https://doi.org/10.23719/1528587 ref. 43.

## Code availability

All analysis code and processing steps are available in the GitHub repository https://github.com/USEPA/WetlandConnectivity/tree/v1.0 ref. 57.

## Supplementary Material

Supplement1

## Figures and Tables

**Table 1 |:** Expected connectivity and biogeochemical behaviours of four wetland hydrologic connectivity classes

Wetland hydrologic connectivity class	Flowpath	Expected connectivity behaviour			Expected biogeochemical behaviour	
		Periodicity	Direction	Velocity	Residence time	Reaction matrix^a^
Riparian	Surface	Frequent	Bidirectional	Rapid	Short	Organic
	Subsurface	Frequent	Bidirectional	Intermediate^b^	Short	Organic/mineral
NRShw	Surface^c^	Infrequent	Lateral	Intermediate	Short	Organic
	Subsurface	Frequent	Lateral	Intermediate	Intermediate	Organic/mineral
NRMid	Surface^d^	Infrequent	Lateral	Intermediate	Short	Organic
	Subsurface	Frequent	Lateral	Slow	Long	Mineral
NRDeep	Surface^e^	Very infrequent	Lateral	Intermediate	Short	Organic
	Subsurface	Constant	Lateral	Very slow	Very long	Mineral/bedrock

a Reaction matrix refers to the soil characteristics of the flowpath from the wetland to the downstream water. These include general physical characteristics of the soil profile, specifically whether organic, mineral, bedrock or a mix of these materials are assumed to be present, given the particular flowpath depth.

b Including hyporheic flows.

c Surface flows due to saturation excess overland flow^17^.

d Surface flows due to infiltration excess overland flow^17^.

e Surface flows due to fill and spill^18^.

**Table 2 |:** Geospatial and modelling analysis results by wetland hydrologic connectivity class and CONUS.

	Wetland statistics^a^					
	Wetland hydrologic connectivity class^b^					
	Riparian	NRShw	NRMid	NRDeep	Unclassified	CONUS
Total wetlands (number)^c^	2,679,963	621,659	1,646,321	1,702,633	12,417	6,662,993
Total wetland area (km^2^)	294,410	35,767	43,399	38,992	644	413,211
Mean area per wetland (km^2^)^c^	0.11	0.06	0.03	0.02	0.05	0.06
Wetlands (%)^d^	3.84	0.47	0.57	0.51	0.01	5.39
	Biogeochemical responses^e^					
	Wetland hydrologic connectivity class^b^					
	Connectivity trend^f^		Riparian	NRShw	NRMid	NRDeep
Acidification^g^	▴		▴	▵	▵	▾
Brownification^h^	▴		▴	▴	▴	▴
Eutrophication^i^	▶◀		▶◀	▶◀	▶◀	▶◀
Sedimentation^j^	▶◀		▿	▶◀	▶◀	▶◀

a Wetland statistics based on analysis of 2011 NLCD wetland data (Methods).

b Riparian wetlands (highest connectivity); NRShw (moderate–high connectivity); NRMid (moderate–low connectivity); NRDeep (lowest connectivity); CONUS, wetlands aggregated over the entire CONUS.

c Wetland number is limited based on the 30 m pixel size of the NLCD used for detecting wetlands. This was not sufficient for detecting small wetlands, such as vernal pools. It also creates a bias in our values of total wetlands, which will be too small, and mean area per wetland, which will be too large.

d Wetland classes as a per cent of total CONUS area.

e Summary of overall biogeochemical responses from linear mixed effects modelling (for details on the modelling approach, see Methods and for specific results, see Supplementary Table 3). Symbols: ▴,▾ >50% of the constituents within the functional group have a positive or negative median relationship with the wetland class, respectively. ▵,▿ >0–50% of the constituents within the functional group have a positive or negative median relationship with the wetland class, respectively. ▶◀ None of the constituents within the functional group has a median relationship with the wetland class that is significantly different than zero.

f The trend of the standardized population mean regression slope as connectivity increases with wetland hydrologic connectivity class; Fig. 3.

g Aluminium; cond, calcium, magnesium, pH and ANC are basic constituents and have the opposite relationship to aluminium (Fig. 3).

h DOC and colour.

i Nitrate.

j TSS.
