# Supplementary material for: National hydrologic connectivity classification links wetlands with stream water quality
Source: Nat Water. Author manuscript; Available in PMC 2024 Apr 6. (PMC10302404; doi:10.1038/s44221-023-00057-w)
Supplement: Supplement1 [file NIHMS1908354-supplement-Supplement1.pdf]

nature water

Article https://doi.org/10.1038/s44221-023-00057-w

National hydrologic connectivity classification links wetlands with stream water quality

In the format provided by the
authors and unedited

Table of Contents

Wetland Classification Assessment	Pg. 2
Figs. S1 to S10	Pg. 9
Tables S1 to S5	Pg. 19
References	Pg. 29
Mixed Effects Models - Four Wetland Classes	Pg. 34
Mixed Effects Models – Single Wetland Class	Pg. 53

Wetland Classification Assessment

We qualitatively estimated the expected magnitudes of the wetland connectivity classes and compared these with quantitative results on the areal distributions of these classes for six case study regions (Methods, Fig. S1, Table S1). As discussed below, we overpredicted the wetland area in three cases (NRShw California vernal pools, NRMid Pocosins and Carolina Bays, and NRMid Southern Florida), and underpredicted area in one case (NRMid prairie potholes). Overall, there was good concurrence between the expected magnitudes of wetland connectivity classes and quantitative results. Spatial patterns in the distribution of the dominant connectivity classes across the six regions are shown in Fig. S10.

California Vernal Pools

West coast vernal pools are small, shallow, depressional wetlands in the low-elevation, Mediterranean climates of southern Oregon, California, and northern Baja California[1]. West coast vernal pools occur on many geological surfaces, but in all cases are underlain by low-permeability layers such as claypans or hardpans (e.g., silica-cemented duripans), clay-rich soils, mudflows or lahars, or bedrock[2-6]. These low-permeability layers are typically associated with specific geological formations, landforms, and soils. As a result, vernal pools in California (Fig. S4) are typically clustered into vernal pool landscapes[7], which are special cases of the hydrologic landscapes described by Winter[8]. California vernal pool landscapes are particularly prevalent, covering more than 4,100 km^2, or more than 5% of the total land surface of the Central Valley[9].

For this region, annual precipitation is strongly seasonal, and typically occurs during a few discrete storms[10]. Surface water and shallow groundwater storage are limited due to the shallow microtopographic relief and the low-permeability layers in the shallow subsurface[3,4,11]. This limited storage fills rapidly with the onset of the wet season and/or individual storms[4]. Once this limited storage is filled, subsequent precipitation initiates immediate inundation and subsequent runoff to downgradient waters[3,4,11]. The duration of runoff is a function of precipitation, which is both strongly seasonal and highly variable[10,11]. Therefore, the annual duration of runoff to downgradient waters is also highly variable, ranging from a few weeks to many months[3,4,11].

As a result of this hydrology, individual California vernal pools can occur as surface-water isolated wetlands. These may still be connected to one another and to downgradient waters by groundwater flows. This may occur as shallow groundwater flow perched on low-permeability layers in the shallow subsurface[4], which we expect to appear as NRShw wetlands. This can also occur as deep groundwater flow as part of regional recharge to deeper aquifers[12], which we would expect to occur as NRDeep wetlands. However, California vernal pools, swales, and headwater streams more commonly develop into integrated flow networks, forming the headward extent of small, low-elevation watersheds[3,4,11]. As a result, we expect most wetlands in this region to be Riparian.

Our connectivity classes were largely consistent with these expectations. Riparian, NRDeep, NRMid, and NRShw classes comprised 20,404 (53%), 11,076 (29%), 5304 (14%), and 1454 (4%) of the region's wetlands by number (percent), respectively. Riparian and NRDeep wetland abundance were an order of magnitude greater than the other two classes. With respect to area, Riparian is by far the dominant class at 1151 km^2 (74%), followed by NRDeep at 291 km^2 (19%), NRMid at 83 km^2 (5%), and NRShw at 32 km^2 (2%); note that we overpredicted NRShw as medium magnitude. Riparian area was an order of magnitude greater than NRDeep, and two

orders of magnitude greater than the remaining two classes. The NRMid and NRShw classes, which we did not predict would occur broadly across the area, each represented 5% or less of the wetland area.

Louisiana Bottomland Hardwoods and Swamps

This region consists of a broad area of forested wetlands that occurs within the floodplains and backwater areas of several major river systems (Fig. S5). This includes the entire Lower Mississippi River Alluvial Valley (LMRAV) occurring within Louisiana (e.g., the Mississippi, Atchafalaya, Ouachita, Boeuf, and Tensas Rivers and Bayou Macon), plus the Red River which originates from the west in the southern Rockies. The Ouachita, Boeuf, Tensas, and Red Rivers and Bayou Macon all enter the Mississippi River near where the Mississippi and Atchafalaya Rivers diverge, subsequently taking their own separate pathways to the Gulf of Mexico. In addition to oxbow lakes, the LMRAV contains numerous meander belts – whose widths are a function of the size of the river – that represent old abandoned river channels due to the shifting of the river's course over time[13]. The Atchafalaya River is the largest distributary of the Mississippi and receives 30% of its flow. This river's basin contains the largest remaining bottomland hardwood forests within the conterminous US. The Atchafalaya has been modified to the point where overbank flooding to the floodplain has nearly been eliminated[14].

Sediment deposits on the outside of river bends form natural levees and alluvial ridges[13]. These levees serve as barriers to river water entering the floodplain under conditions that are less than flood stage, i.e., during spring floods. Coarse materials are generally deposited near the channel where energies are highest, and finer-textured materials (i.e., clays) are deposited further from the channel, in backwater areas and floodplain areas where energies are lowest[15]. As a result, low permeability clay soils are common in these areas; i.e., just two clay soils, Sharkey and Fausse, make up 47% of the areal extent of soils in the Mississippi River Alluvial Plain[16].

Alluvial swamps and bottomland hardwoods experience inundation from either high winter precipitation during times when water tables are high due to low evapotranspiration, or overbank flooding from spring flooding[17]. Given that the Mississippi is the largest river in the US, the latter was historically most significant in the LMRAV. This connectivity between the river and floodplain wetlands was characterized by infrequent, high magnitude events that overtopped the natural levee, as described by the flood pulse concept[18,19]. However, the natural hydrology and overbank flooding of these rivers and the adjacent floodplains has been greatly impacted by the construction of flood control structures, especially levees and dams[17,18]. Compared to historical conditions, restriction of floodwaters by levees and dams resulted in higher and more frequent flooding within the levee, and a lower frequency and magnitude of flooding outside of the levee[17]. For those bottomland hardwood and swamp forests outside the levee, this means that their major source of water is no longer overbank flooding, but a combination of precipitation and backwater flooding (inundation from drainage that is impeded by the levee)[17]. However, these forested wetlands can also occur within the embanked floodplain, whose widths can vary from at least 0.4 to 65 km in the LMRAV[18]. Forested wetlands in this setting will therefore be subjected to higher and more frequent flood events.

Given the prominent occurrence of large rivers in this region, we expect Riparian wetlands to be the most abundant and to have the largest area of the four connectivity classes. The remaining floodplain and backwater wetlands are expected to be classified as non-riparian, because they are

not directly in contact with a river. Since those areas are underlain by impermeable clays, we would expect most of these wetlands to be classified as NRDeep; i.e., they would have standing water with deep, slow subsurface flowpaths to nearby rivers.

Our connectivity classes were largely consistent with these expectations. Riparian and NRDeep wetlands occurred in nearly equal numbers at 27,405 and 27,316, respectively. NRMid and NRShw wetlands were an order of magnitude less abundant, at 1401 and 1404, respectively. Together, Riparian and NRDeep represented 48% and 47% of all the region's wetlands. While Riparian and NRDeep were nearly equal in number, the former represented an order of magnitude more area than the latter: 12,386 km^2 for Riparian vs. 1794 km^2 for NRDeep, representing 86% and 13% of the region's wetland area, respectively. NRMid and NRShw wetland areas were 1-2 orders of magnitude smaller than the area of NRDeep: 61 km^2 (0.4%) and 103 km^2 (1%), respectively.

Playa Lakes

Playas are shallow depressional wetlands that are nearly circular and flat bottomed, and primarily occur in the High Plains Region of the western Great Plains; they are found in deserts and semi-arid prairies[20,21]. Playa lakes (Fig. S6) are a subset of 25,000 playas that occur within the Playa Lakes Region of the Southern Great Plains, which extends from southeastern Colorado and southwestern Kansas through eastern New Mexico and western Texas[20]. The highest number and density of playa lakes is found in the Southern High Plains (SHP), which occurs within Texas and New Mexico in the southern portion of the Playa Lakes Region on the Llano Estacado, an elevated escarpment[20,22,23]. Playa lakes in the SHP are circular, with an average area of 6 ha.

Playa lakes are formed through aeolian processes by dissolution of subsurface basin material and wind deflation[20]. Local thunderstorms can provide precipitation for these wetlands during May through September, but they are normally dry in late summer, late winter, and early spring. There can be multiple wet-dry cycles during a single growing season, and these features may not wet up every year depending on precipitation conditions[21].

Playa lakes in the SHP are the lowest points in closed watersheds, and are not interconnected by surface water[22]. The input side of the water balance equation for playa lakes is dominated by precipitation and local runoff, and losses occur through evaporation, transpiration, and infiltration; few receive groundwater inputs[20,21]. Playa lakes are main recharge points for the underlying Ogallala Aquifer[20,21,24]. Different studies have estimated that as little as 15% and as much as 84% of the water in a playa lake will reach the Ogallala Aquifer, with published recharge rates as high as 219 mm/yr, compared to a maximum 0.8 mm/yr recharge rate for the surrounding interplaya area[24]. Recharge does not occur directly through the bottom of playa lakes, since they are lined by Randall clay soils that limit infiltration; instead, infiltration occurs when the water level rises to the level of the surrounding, more permeable upslope soils[22].

The Playa Lake Region contains a few rivers, including the Pawnee, Arkansas, Cimarron, Canadian, Red, and Pecos Rivers; most of these originate in the Rockies to the west. Given these rivers, it is expected that the region will have many Riparian wetlands. Because playa lakes occur in closed watersheds and are not interconnected, they are non-riparian. Since they recharge the Ogalla aquifer, they will tend to have mid to deep flowpaths depending on the depth to the aquifer. Playa lakes on the Llano Estacado are expected to have the deepest flowpaths

(NRDeep), since they are on an escarpment that rises 600-1200 m above sea level with the greatest depth to water in the area[25,26]. In other areas, for example, towards the southern border of the region, playa lakes should have medium depth flowpaths because the water table is closer to the surface[26]. Since the Llano Estacado has the highest density and number of playa lakes, NRDeep playa lakes are expected to exceed NRMid in both number and area.

Our connectivity classes were largely consistent with these expectations. Riparian wetlands are the most abundant in the Playa Lakes Region (7481, or 38%), followed by NRDeep (6182, or 31%), NRMid (5654, or 29%), and NRShw (377, or 2%). The distribution of area follows the same order: Riparian (327 km^2, or 53%), NRDeep (184 km^2, or 30%), NRMid (107 km^2, or 17%), and NRShw (4 km^2, or 1%). Throughout this region, Riparian wetlands dominate in number and area. Of the non-riparian wetlands (i.e., playa lakes), NRDeep slightly exceeds NRMid in number, (31 vs. 29% of total wetland number). However, NRDeep playa lakes exceed NRMid by over 70% with respect to area. NRShw wetlands are an order of magnitude less in abundance compared to the other classes, and two orders of magnitude less in area.

Pocosins and Carolina Bays

The Atlantic Coastal Plain of the United States includes low-lying areas of five southern states bordering the Atlantic Ocean (Virginia, North and South Carolina, Georgia, and Florida) and comprises portions of the Middle Atlantic Coastal Plain, Southeastern Plains, and Southern Coastal Plains ecoregions[27]. Wetlands are abundant on these landscapes[28]; the southern US has the greatest abundance of hydric soils in the US outside of Alaska[29]. Inland wetlands found in the study area include depressional wetlands (e.g., pocosins and Carolina bays), as well as abundant riparian[30] and lake-fringing systems.

Pocosins and Carolina bays (Fig. S7) are forested or shrub-dominated wetlands common along the Atlantic coastal plains[31,32], with highest abundance in the lower and middle coastal plain areas of Georgia, South Carolina, and North Carolina[21]. Pocosins occur frequently in interfluvial flatwoods on saturated soils. Carolina bays occur usually as elongated oval depressions with the long axis aligned northwest to southeast[33]; a sand rim is typical of the southeast border[34]. Other depressional wetland types similar to and co-occurring with Carolina bays and pocosins include Citronelle Ponds[35] in northern Florida and Delmarva bays[36] in the Delaware/Maryland/Virginia peninsula.

Both pocosins and Carolina bays are predominantly ombrotrophic, with precipitation inputs and evapotranspiration outputs as the dominant water balance components[37]. Pocosins are typically characterized by deep organic horizons (e.g., >130 cm) that are frequently saturated year-round, though during droughts pocosins can dry and burn (thereby removing organic matter creating pond-like openings[32]. Carolina bays are depressions underlain by sandy clay hardpans with some localized groundwater interactions[38]. Most bays and pocosins lack natural surface-water outlets[32,39].

As pocosins and Carolina bays are typically not surface water connected, the lack of natural drainage outlets coupled with low water permeability through clay hard pans and extensive peaty soils also limiting water movement would suggest both NRShw and NRMid as predominant connectivity types in coastal plains with extensive pocosin and Carolina bay abundance. The difference would hinge on the drainage characteristics of the system. However, pocosins and

Carolina bays that do have surface water connections to streams or riparian wetlands would be classified as Riparian (e.g., Fig. S7). In addition, the extensive lotic network occurring across the southeastern United States with abundant floodplain wetlands[30] suggests an abundance of Riparian wetland classes.

Our connectivity classes were largely consistent with these expectations. NRShw was the most abundant wetland class (108,013, or 36%) followed closely by NRMid (96,248, or 32%) and Riparian (85,264, or 29%). Very few NRDeep wetlands occur in the area (8973, or 3%). Except for NRDeep, the distribution by count was evenly distributed, with NRShw, Riparian, and NRMid all within 29-36% of the distribution. With respect to area, however, Riparian dominated by an order of magnitude (32,479 km^2, or 77%), over NRShw and NRMid, which both had somewhat similar areas (5487 km^2, or 13%, and 3793 km^2, or 9%, respectively). However, we overpredicted NRMid as medium magnitude. NRDeep had two orders of magnitude less area than Riparian (410 km^2, or 1%). Greater amounts of NRShw wetlands were found in the lower coastal plain region (Fig. S10), where highly organic soils with high soil water-holding capacities (i.e., pocosins with highly organic soils overlain by peat) predominate the interfluvial areas. The middle and upper coastal plain have relatively less organic matter and higher slopes, facilitating drainage through permeable soils – though the water table is frequently near the surface, decreasing capacity.

Prairie Potholes

The Prairie Pothole Region (PPR) covers much of the Northern Great Plains of the US and Canada, with the US PPR extending across large portions of North and South Dakota, Minnesota, Iowa, and into Montana. Formed by the deposition of glacial till and the retreat of the most recent glaciation (10,000-18,000 years ago), the PPR is home to high densities of depressional wetlands (prairie potholes; Fig. S8) and small lakes. Most potholes have small catchments that are primarily fed by winter snowmelt and spring/summer rains. Water losses are primarily via evapotranspiration, which exceeds precipitation in the Dakotas and Montana – resulting in high salinities for some wetlands with no outlets[40]. Prairie potholes are typically underlain by silty-clay soils with low permeability and poor drainage. Thus, while proximal wetlands may connect via shallow groundwater[41-43], substantial subsurface flows likely occur only in areas of sand lenses[44,45] or along moraine edges[46]. During very wet periods, overland connections have been shown to occur where potholes will fill and spill over or merge together[47-50], with some potentially connecting to the stream network[50,51]. Due to these features, the dominant wetland class for prairie potholes should be NRDeep wetlands, i.e., those with infrequent surface flows and limited or slow/deep groundwater flows.

While the stream-river network itself is geologically young in the PPR, riparian wetlands are also present in the lower portions of the watersheds where river floodplains exist. However, the eastern portion is fertile agricultural land (US Corn Belt) and many of the natural potholes have been lost due to extensive artificial drainage (e.g., subsurface tiles have drained 96% of wetlands from the PPR portion of Iowa[52]). Thus, the proportion of Riparian wetlands in the row-crop dominated eastern PPR should be more prominent, as remaining wetlands will primarily be associated with the rivers and floodplains and fall into the Riparian class.

Our connectivity classes were largely consistent with these expectations. NRDeep was the most abundant class (253,771, or 66%), which was an order of magnitude greater than both Riparian

(65,443, or 17%) and NRMid (57,858, or 15%). NRShw abundance was two orders of magnitude less than NRDeep (9615, or 2%). NRDeep also dominates the region by area, although not as strongly, since these depressional wetlands tend to have small individual areas. Areas were 5958 km^2 (54%) for NRDeep, 3348 km^2 (30%) for Riparian, 1508 km^2 (14%) for NRMid, and 224 km^2 (2%) for NRShw. NRMid was underpredicted as low magnitude, however. As expected, with the influence of agriculture and wetland loss, the western portion in North and South Dakota was dominated by NRDeep, while the agricultural portions in Minnesota and Iowa had higher numbers of riparian wetlands (Fig. S10).

Southern Florida

The Southern Florida (or peninsular Florida) region includes the Southern Florida Flatwoods (155), Southern Florida Lowlands (156B), and the Florida Everglades and Associated Areas (156A) Major Land Resource Areas[53] (MLRAs). The region has low elevation, often not more than six meters above sea level, with abundant wetlands and poorly drained soils; land cover is dominated by wetland vegetation[53]. This is borne out by the reported abundance of wetlands detailed in the region by many researchers[28,54,55] as well as the presence of the iconic Everglades wetlands[56].

Soils are characterized as typically sandy soils in the northern portions of the peninsula, with peaty soils dominating in the southern portions, then grading to limerock-influenced soils in the extreme south[57,58]. Surface runoff is generally a function of precipitation less evapotranspiration. Florida's subtropical climate results in widespread and abundant precipitation (especially during the summer), partially offset by high evaporative rates[59]. Annual precipitation typically varies from 112-165 cm, while evaporative demands are lower (117-137 cm). Some 1700 rivers drain the state, and the near-surface water table is often revealed during periods of high rainfall[60,61].

The many different types of freshwater wetlands on the Florida peninsula (Fig. S9) include blackwater and spring-fed swamps[62], forested and herbaceous sloughs[55], widespread marshlands[56], and forested, herbaceous, and open-water non-floodplain wetlands[63] (also known as geographically isolated wetlands[21]). Connectivity will favor Riparian wetlands due to the abundance of streams and rivers on the peninsula. Both NRShw and NRMid connectivity classes are also expected, because of the ample non-floodplain wetlands on the landscape as well as the presence of both sandy and peaty soils throughout[64]. NRShw is expected to dominate in areas over poorly drained siliceous soils abundant in sand as well as some areas of peaty soils and clayey substrata[64] and in areas like the non-riparian portions of the Everglades, where the water table is at or above the surface for much of the year.

Our connectivity classes were largely consistent with these expectations. NRShw wetlands strongly dominate the number of Southern Florida wetlands (114,226, or 68%), followed by Riparian (28,685, or 17%), NRMid (22,863, or 14%), and NRDeep (1877, or 1%). In terms of area, however, Riparian is the dominant connectivity class, with 17,615 km^2 (57%). This is followed by NRShw with 11,842 km^2 (39%). The NRMid and NRDeep classes represent much less wetland area, 1141 km^2 (4%) and 68 km^2 (0.2%), respectively, although we overpredicted NRMid as medium magnitude. The Riparian and NRMid wetland classes are especially prevalent within the Southern Florida Flatwoods and Florida Everglades and Associated Areas MLRAs. NRMid wetlands, which make up a small proportion (4%) of the total area, are found

predominantly in eastern regions of the southern peninsula (Fig. S10), encompassing both the Southern Florida Flatwoods and Southern Florida Lowlands.

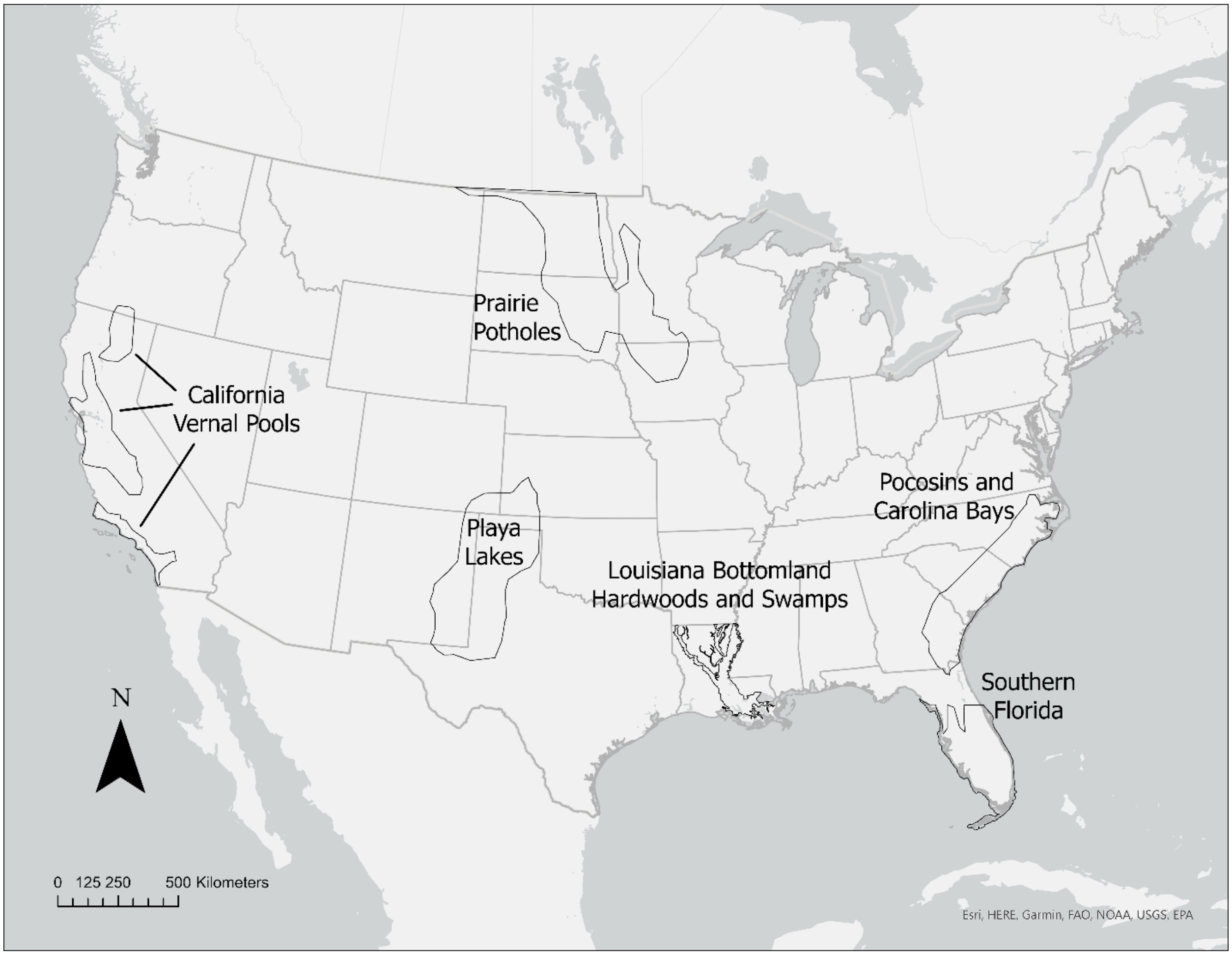

Fig. S1. Locations of six regional case studies used for validation of connectivity classification. Regions for California vernal pools, playa lakes, pocosins and Carolina bays and prairie potholes based on Tiner[21]. Louisiana bottomland hardwoods and swamps area from a map[65] based on US EPA[27]. Southern Florida is the area south of a karst region that covers most of northwestern Florida[21], extended to the eastern coast.

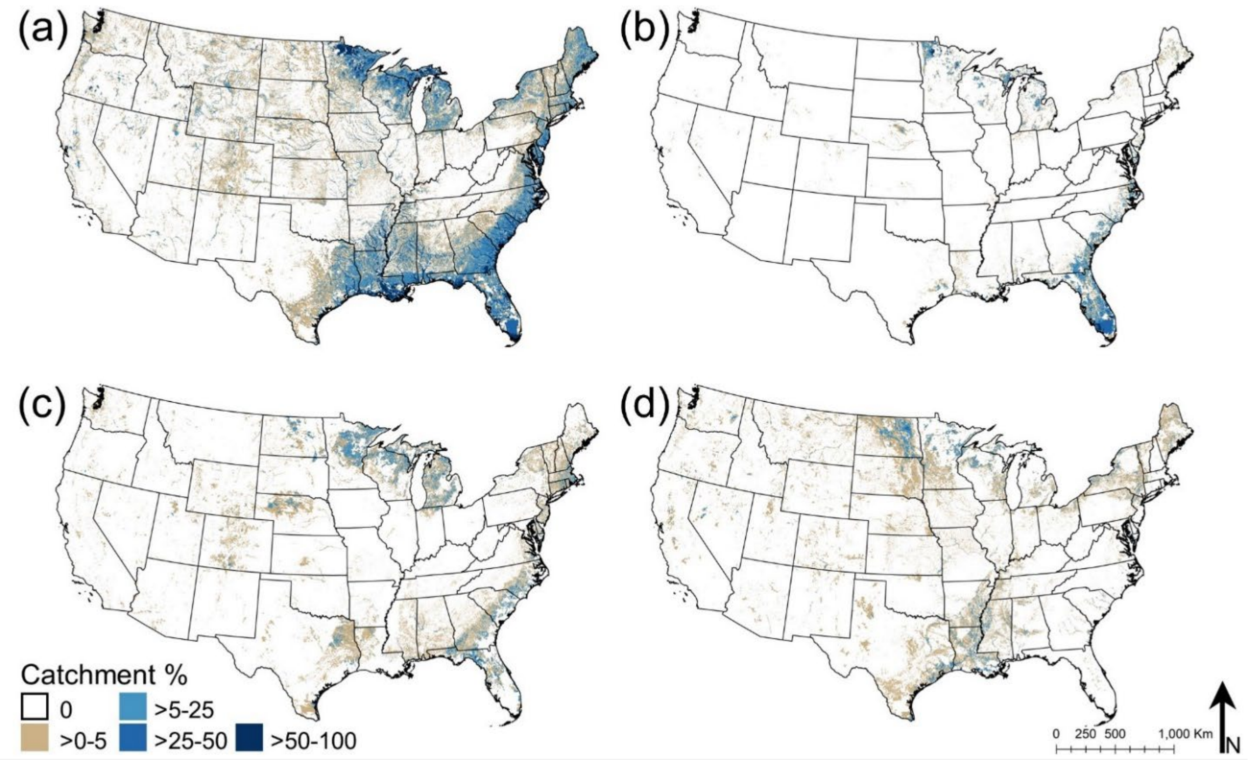

Fig. S2. Four wetland hydrologic connectivity classes as a percent of total land cover within the NHDPlusV2 catchment. (a) Riparian wetlands. (b) Non-riparian shallow wetlands (NRShw). (c) Non-riparian mid-depth wetlands (NRMid). (d) Non-riparian deep wetlands (NRDeep).

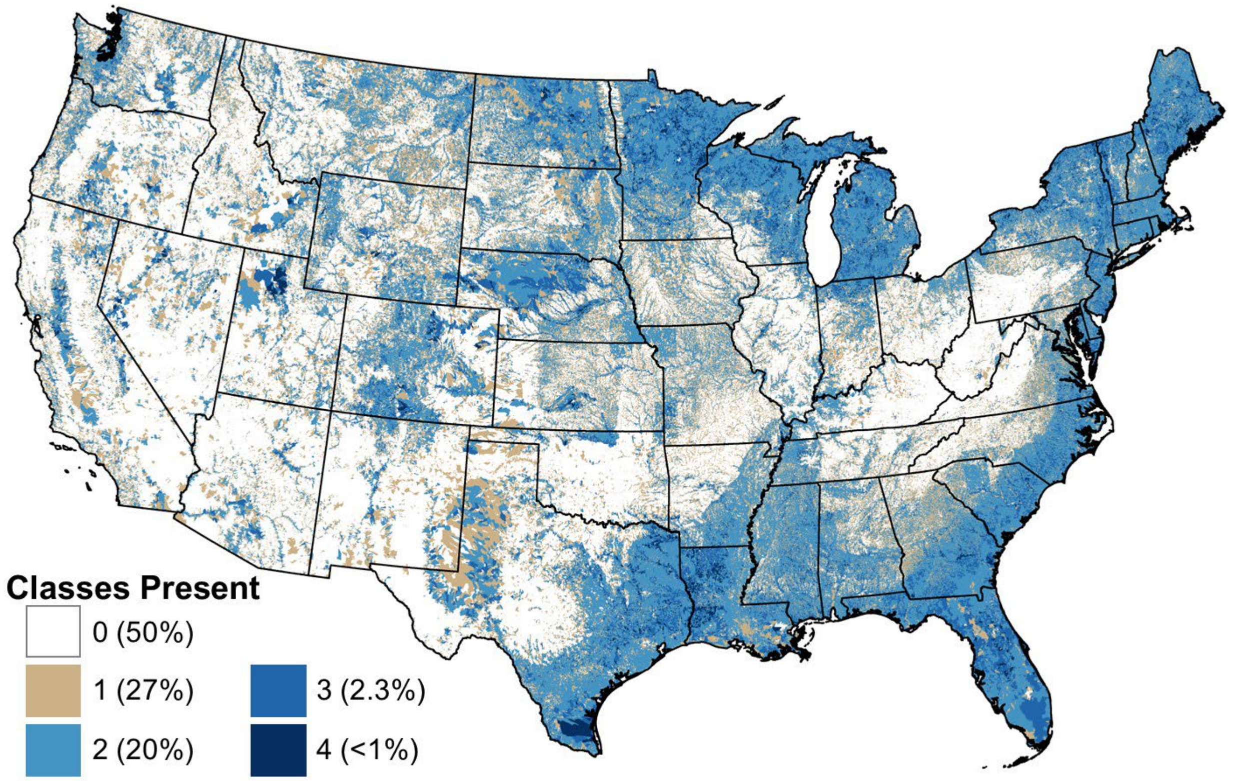

Fig. S3. Number of wetland hydrologic connectivity classes present by NHDPlusV2 catchment. Parenthetical values in key indicate the percent of catchments across the conterminous US containing the associated number of connectivity classes; e.g., half of all catchments have no wetlands, 27% of all catchments contain only one wetland class, while less than one percent of all catchments contain all four wetland classes.

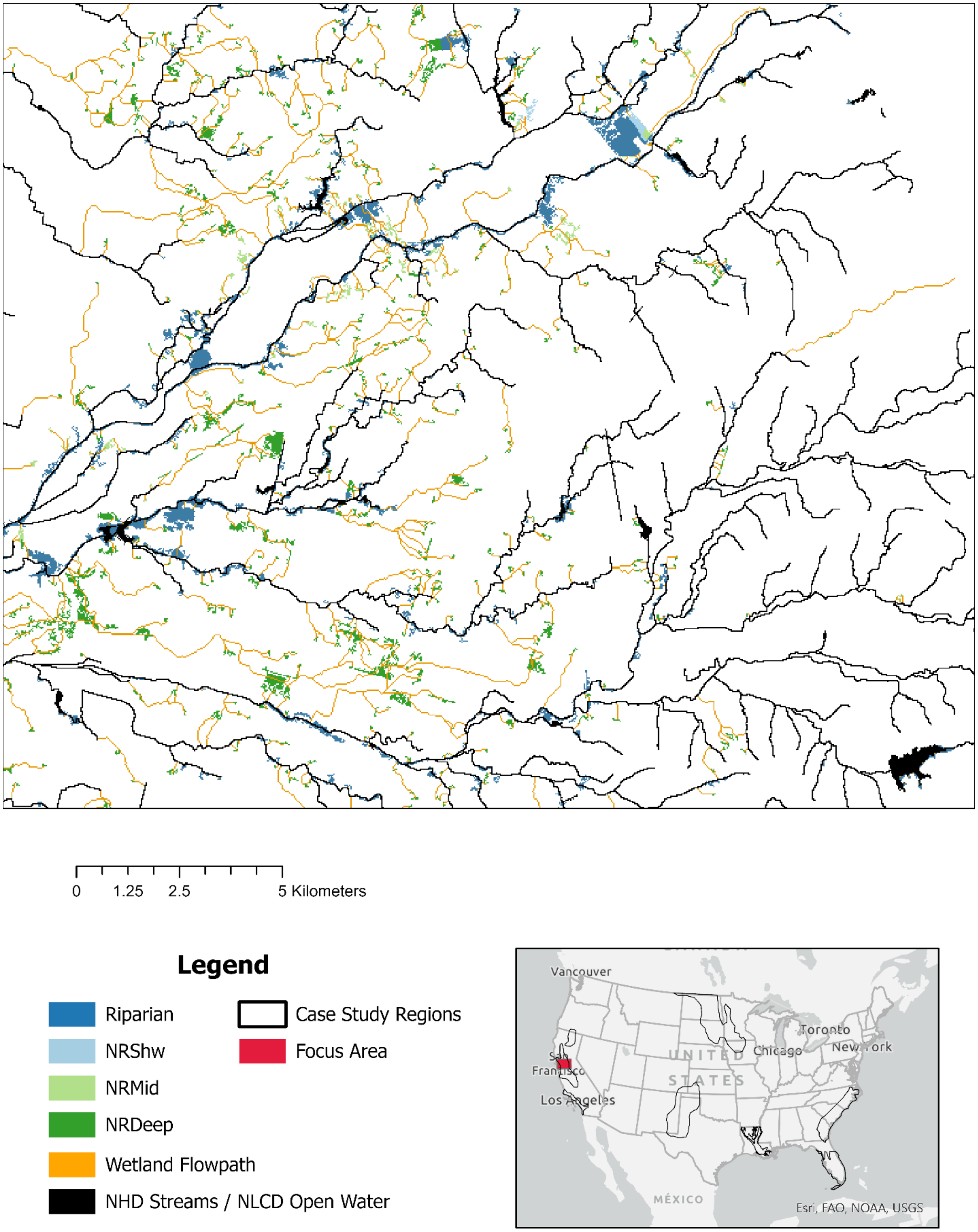

Fig. S4. Area within California vernal pools case study region showing example of individual wetlands by connectivity class. Although region is named for vernal pools, other wetland types may occur and would be incorporated into the analysis.

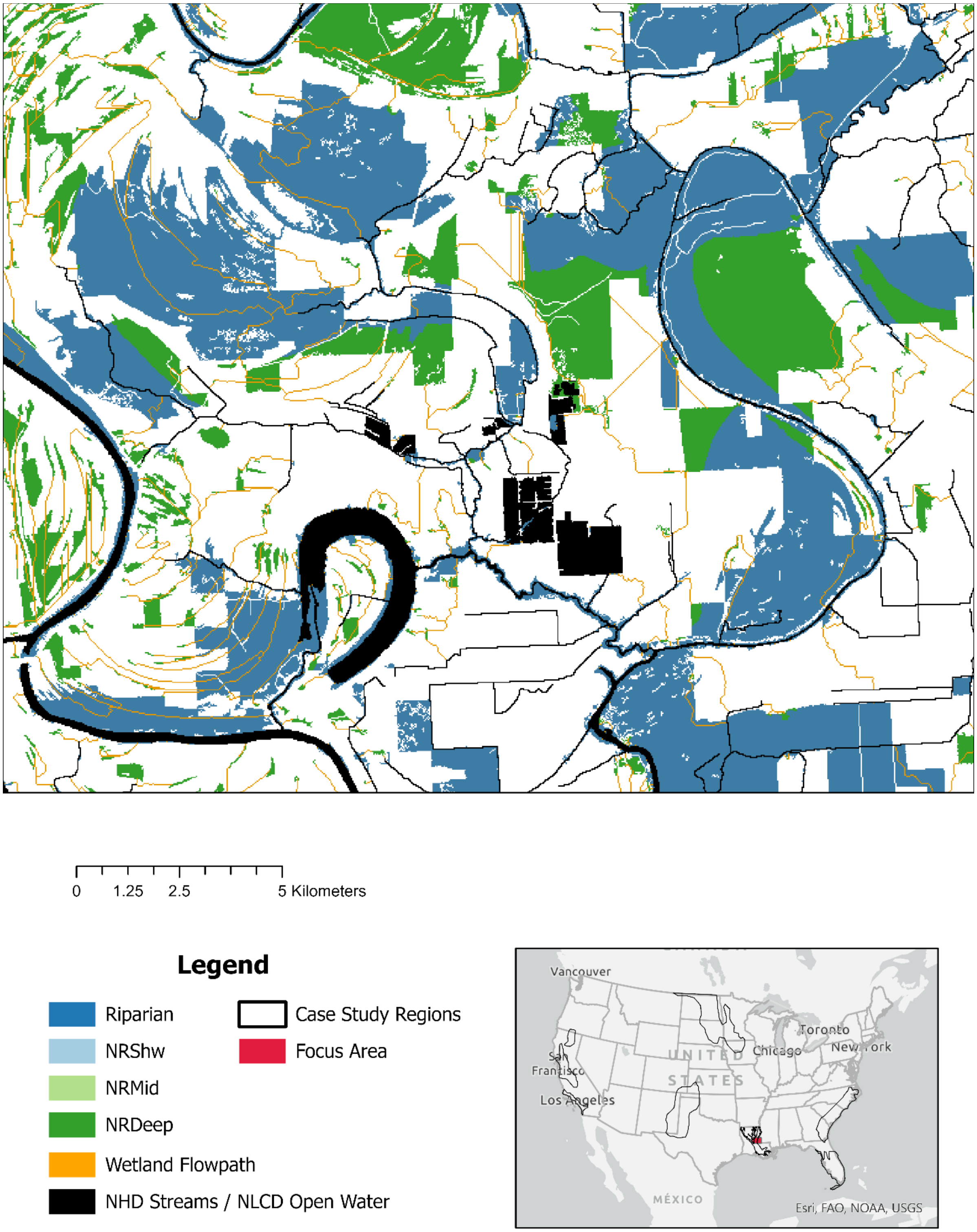

Fig. S5. Area within Louisiana bottomland hardwoods and swamps case study region showing example of individual wetlands by connectivity class. Although region is named for bottomland hardwoods and swamps, other wetland types may occur and would be incorporated into the analysis.

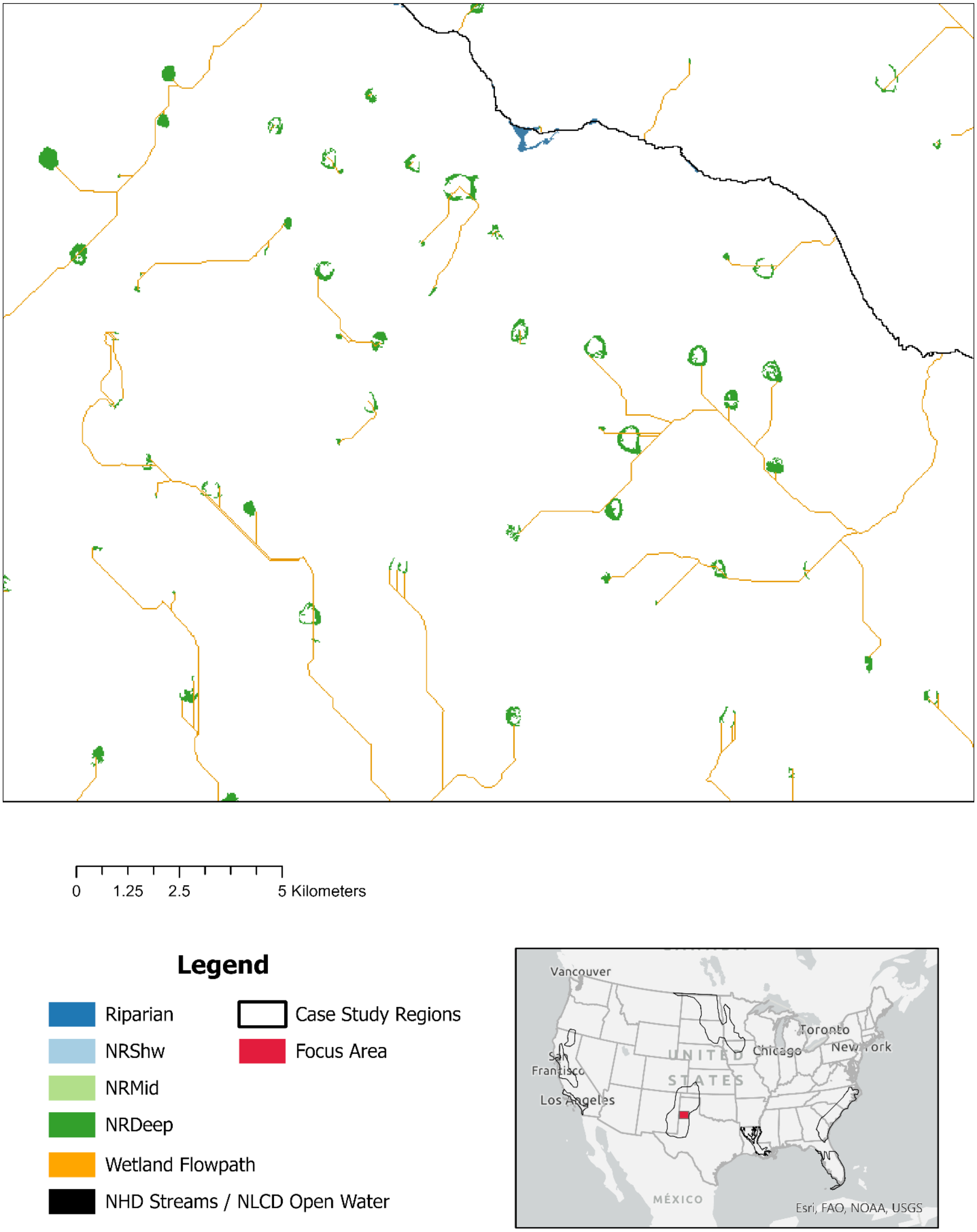

Fig. S6. Area within playa lakes case study region showing example of individual wetlands by connectivity class. Although region is named for playa lakes, other wetland types may occur and would be incorporated into the analysis.

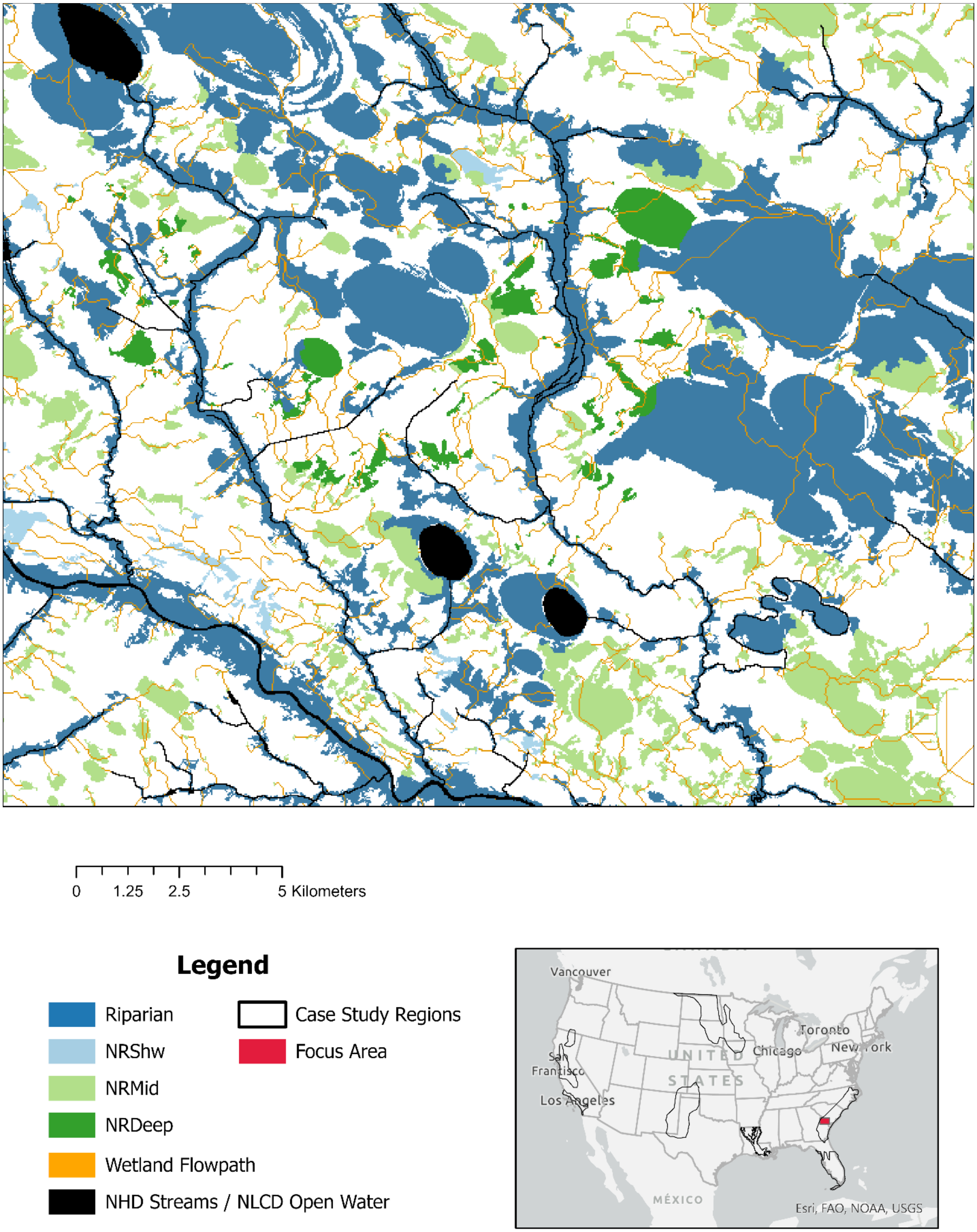

Fig. S7. Area within pocosins and Carolina bays case study region showing example of individual wetlands by connectivity class. Although region is named for pocosins and Carolina bays, other wetland types may occur and would be incorporated into the analysis.

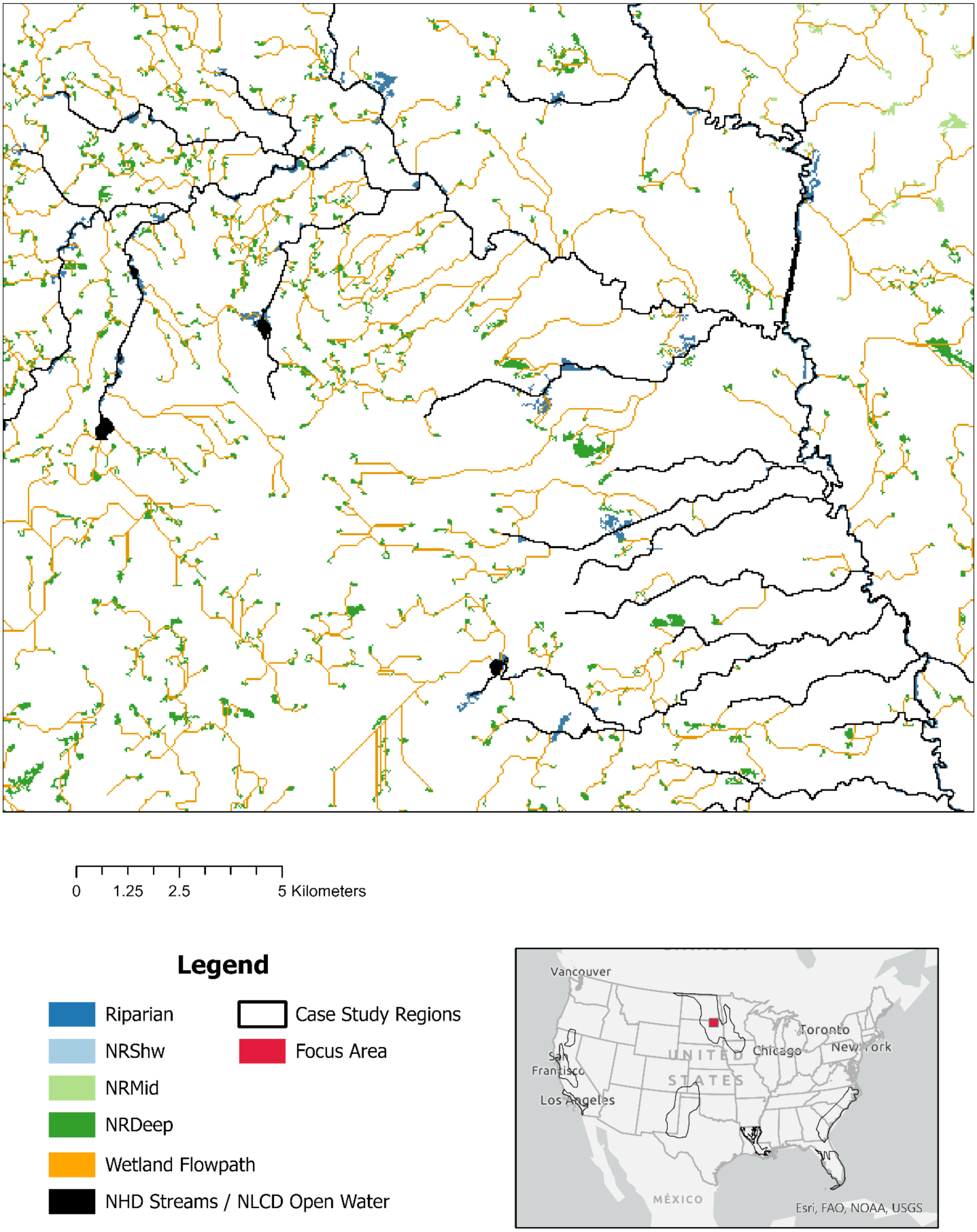

Fig. S8. Area within prairie potholes case study region showing example of individual wetlands by connectivity class. Although region is named for prairie potholes, other wetland types may occur and would be incorporated into the analysis.

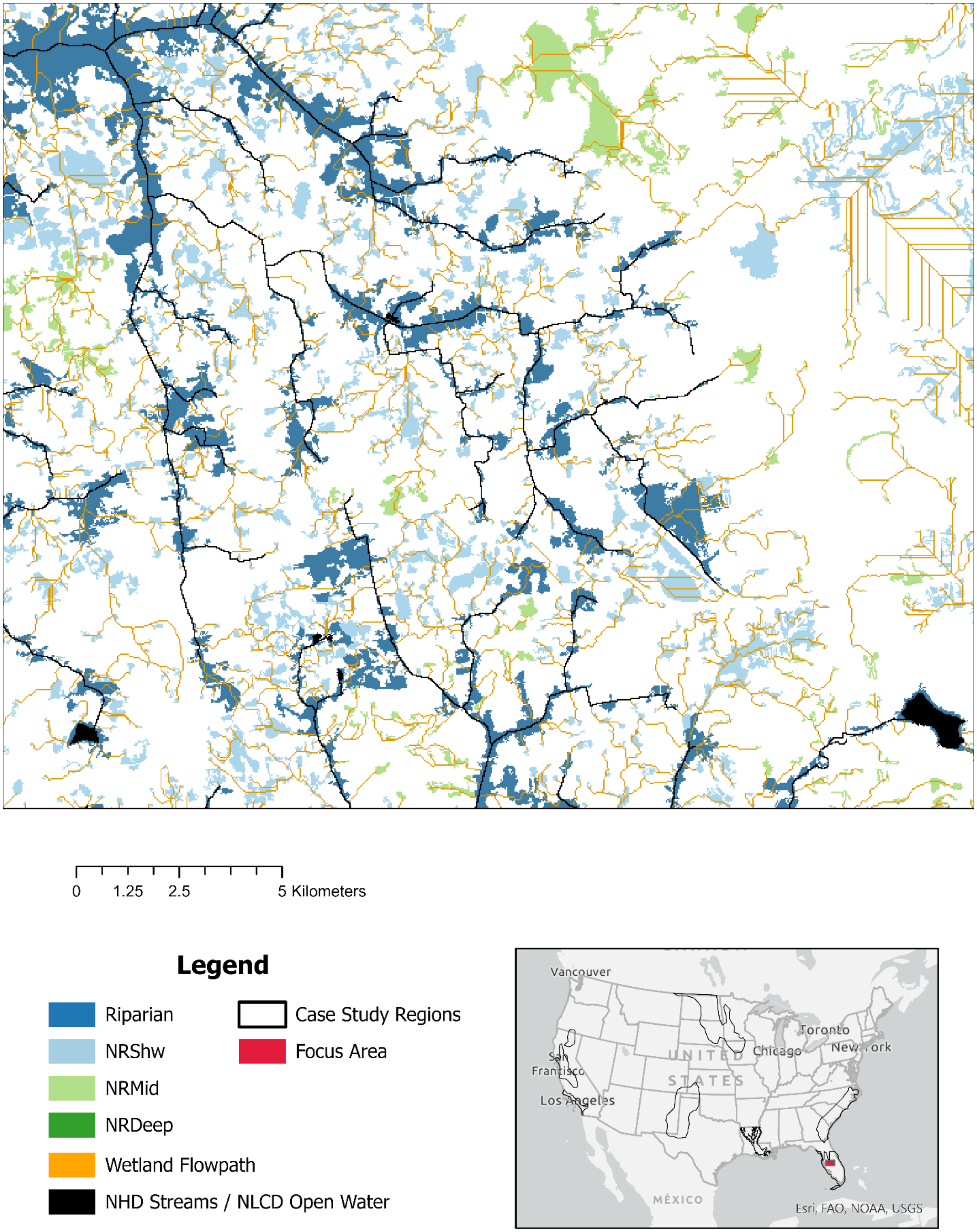

Fig. S9. Area within Southern Florida case study region showing example of individual wetlands by connectivity class.

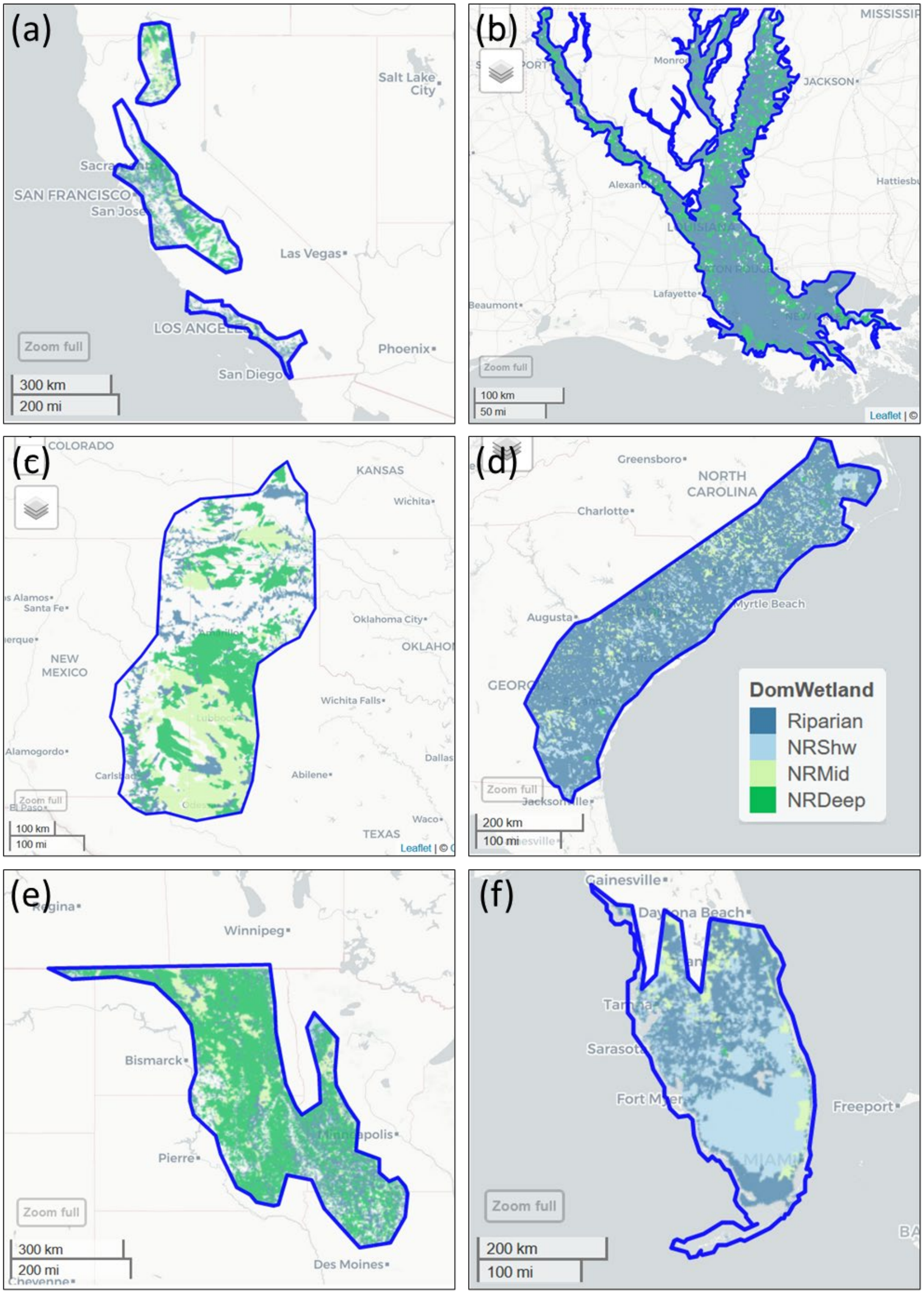

Fig. S10. Dominant wetland hydrologic connectivity class within NHDPlusV2 catchment across six regional case study locations: (a) California vernal pools, (b) Louisiana bottomland hardwoods and swamps, (c) playa lakes, (d) prairie potholes, (e) pocosins and Carolina bays, and (f) Southern Florida. See Fig. S1 for locations within conterminous US.

Table S1. Expected magnitude, number of wetlands, percent by number, wetland area, and percent by area for wetland connectivity classes in six regional case studies (see Fig. S1). Expected magnitude is a categorical assessment of the relative area of wetlands within the region based on a combination of the literature, first principals, and familiarity with these regions; low, medium, and high categories were considered to be comprised of 0-10%, 11-50%, and 51-100% wetland area, respectively. Expected values in **bold** were consistent with actual wetland area values. Although each region except Southern Florida is named for iconic wetlands, other wetlands occur within the regions and were incorporated into the analysis.

**Variable**	**Wetland Hydrologic Connectivity Class***				
	**Riparian**	**NRShw**	**NRMid**	**NRDeep**	**Total**
	California Vernal Pools				
Expected magnitude	**High**	Medium	**Low**	**Medium**	
Number of wetlands	20401	1454	5304	11076	38235
Percent by number	53.4	3.8	13.9	29.0	100
Wetland area (km^2)	1151.1	32.4	82.8	290.8	1557.0
Percent by area	73.9	2.1	5.3	18.7	100
	Louisiana Bottomland Hardwoods and Swamps				
Expected magnitude	**High**	**Low**	**Low**	**Medium**	
Number of wetlands	27405	1404	1401	27316	57526
Percent by number	47.6	2.4	2.4	47.5	100
Wetland area (km^2)	12386.4	102.9	61.5	1794.2	14345.0
Percent by area	86.4	0.7	0.4	12.5	100
	Playa Lakes				
Expected magnitude	**High**	**Low**	**Medium**	**Medium**	
Number of wetlands	7481	377	5654	6182	19694
Percent by number	38.0	1.9	28.7	31.4	100
Wetland area (km^2)	326.8	3.7	106.7	184.0	621.1
Percent by area	52.6	0.6	17.2	29.6	100
	Pocosins and Carolina Bays				
Expected magnitude	**High**	**Medium**	Medium	**Low**	
Number of wetlands	85264	108013	96248	8973	298498
Percent by number	28.6	36.2	32.2	3.0	100
Wetland area (km^2)	32478.6	5487.4	3793.1	410.4	42169.4
Percent by area	77.0	13.0	9.0	1.0	100
	Prairie Potholes				
Expected magnitude	**Medium**	**Low**	Low	**High**	
Number of wetlands	65443	9615	57858	253771	386687
Percent by number	16.9	2.5	15.0	65.6	100
Wetland area (km^2)	3348.3	223.8	1507.7	5957.6	11037.4
Percent by area	30.3	2.0	13.7	54.0	100
	Southern Florida				
Expected magnitude	**High**	**Medium**	Medium	**Low**	
Number of wetlands	28685	114226	22863	1877	167651

Percent by number	17.1	68.1	13.6	1.1	100
Wetland area (km^2)	17614.9	11824.2	1141.4	68.3	30648.8
Percent by area	57.5	38.6	3.7	0.2	100

*Riparian wetlands (highest connectivity); NRShw=non-riparian shallow wetlands (moderate-high connectivity); NRMid=non-riparian mid-depth wetlands (moderate-low connectivity); NRDeep=non-riparian deep wetlands (lowest connectivity).

Table S2. List and description of response and predictor variables used in mixed effect modeling of 11 stream water quality constituents. Predictor variables with dashes in the transform column were not transformed. NRSA = National Rivers and Streams Assessment. *Present analysis

Variable	Description	Transform	Mean	SD	Source
	Response Variables				
Al	Total dissolved aluminum (mg/L)	ln	-4.793	1.478	[66]
ANC	Acid neutralizing capacity (µeq/L)	ln	7.597	0.896	[66]
pH	Laboratory measured pH	^4	4024.08	1014.66	[66]
Ca	Calcium (mg/L)	ln	3.218	1.283	[66]
Mg	Magnesium (mg/L)	ln	2.082	1.369	[66]
Cond	Specific conductance (µS/cm)	ln	5.672	1.235	[66]
DOC	Dissolved organic carbon (mg/L)	ln	1.185	0.872	[66]
Color	True color (APHA Pt-Co Units)	ln	2.855	0.837	[66]
NO_3	Nitrate (NO_3^-) as nitrogen (mgN/L)	ln	-1.618	1.757	[66]
TSS	Total suspended solids (mg/L)	ln	2.244	1.713	[66]
Turb	Turbidity (Nephelometric Turbidity Unit)	ln	1.846	1.623	[66]
	Predictor Variables				
pctRipar	% of watershed area classified as Riparian wetlands	–	3.126	6.663	*
pctNRShallow	% of watershed area classified as NRShw wetlands	–	0.317	1.770	*
pctNRMid	% of watershed area classified as NRMid wetlands	–	0.686	1.804	*
pctNRDeep	% of watershed area classified as NRDeep wetlands	–	0.413	0.996	*
AgKffactWs	Mean soil erodibility (Kf) factor (unitless) of soils within watershed on agricultural land. The Kf factor is used in	–	0.072	0.087	[67]

	the Universal Soil Loss Equation (USLE) and represents a relative index of susceptibility of bare, cultivated soil to particle detachment and transport by rainfall				
Al2O3Ws	Mean % of lithological aluminum oxide (Al_2O_3) content in surface or near surface geology within watershed	–	10.030	3.373	67
BFIWs	Baseflow is the component of streamflow that can be attributed to ground-water discharge into streams. The Baseflow Index (BFI) is the ratio of baseflow to total flow, expressed as a percentage within watershed	–	45.542	16.345	67
CaOWs	Mean % of lithological calcium oxide (CaO) content in surface or near surface geology within watershed	–	8.029	7.234	67
ClayWs	Mean % clay content of soils (STATSGO) within watershed	–	22.849	9.426	67
CompStrgthWs	Mean lithological uniaxial compressive strength (megaPascals) content in surface or near surface geology within watershed	–	74.552	45.450	67
HydrlCondWs	Mean lithological hydraulic	–	14.245	28.879	67

	conductivity (micrometers per second) content in surface or near surface geology within watershed				
K2OWs	Mean % of lithological potassium oxide (K_2O) content in surface or near surface geology within watershed	–	1.926	0.637	[67]
KgNperWsArea	Mean rate of fertilizer, cultivated biological nitrogen fixation (CBNF), and manure application to agricultural land from confined animal feeding operations in kg N/ha/yr, within watershed	ln	2.831	1.105	[67]
MgOWs	Mean % of lithological magnesium oxide (MgO) content in surface or near surface geology within watershed	–	2.786	2.009	[67]
Na2OWs	Mean % of lithological sodium oxide (Na_2O) content in surface or near surface geology within watershed	–	1.344	0.862	[67]
NWs	Mean % of lithological nitrogen (N) content in surface or near surface geology within watershed	–	0.174	0.202	[67]
OmWs	Mean organic matter content (% by weight) of soils (STATSGO) within watershed	–	1.596	2.621	[67]

P2O5Ws	Mean % of lithological phosphorous oxide (P_2O_5) content in surface or near surface geology within watershed	–	0.174	0.086	[67]
SiO2Ws	Mean % of lithological silicon dioxide (SiO_2) content in surface or near surface geology within watershed	–	54.322	11.756	[67]
SN_2008Ws	Annual gradient map of precipitation-weighted mean deposition for average sulfur & nitrogen wet deposition for 2008 in kg of S+N/ha/yr within watershed	–	437.194	210.571	[67]
SWs	Mean % of lithological sulfur (S) content in surface or near surface geology within watershed	–	0.274	0.484	[67]
WsArea	Watershed area (km^2) at NHDPlus stream segment outlet, i.e., at the most downstream location of the vector line segment	ln	6.122	3.457	[67]
imp.ws	Mean imperviousness of anthropogenic surfaces (NLCD 2006) within watershed	ln	0.592	0.606	[68]
for.ws	% of watershed area classified as deciduous and coniferous forest land covers (NLCD 2006 class 41 and 42)	–	39.883	29.813	[68]
prec	Mean annual precipitation (mm)	–	948.739	380.879	[68]

pt.N.aw	Point Source N (kg N/ha/yr)	ln	0.152	0.385	[68]
ag.ws	% of watershed area classified as crop and hay land use (NLCD 2006 class 81 and 82)	–	23.227	26.453	[68]
subsz	Mean substrate size (mm)	log10	0.144	1.329	[67,68]

Table S3. Standardized population mean regression slopes [confidence intervals] for four wetland hydrologic connectivity classes and model performances for four groups of stream constituents using linear mixed effects models. The slope represents the standardized relationship between the constituent and wetland averaged across all regions. Bold type indicates that the regression slope plus or minus the confidence interval (two times the standard error of the slope estimate) did not overlap with zero. Twenty-four physiographic regions (https://water.usgs.gov/GIS/metadata/usgswrd/XML/physio.xml) were used as the grouping factor for mixed effects modeling for all constituents except for NO_3, where the nine NRSA ecoregions[69] were used. Wetland hydrologic connectivity classes are listed in order of ascending connectivity. Note that the importance of wetlands relative to other watershed metrics varied between constituents; full results for all covariates available in this Supplement as "Mixed Effects Models - Four Wetland Classes" and "Mixed Effects Models - Single Wetland Class". Param Range reports the range of standardized parameter values present in each model, including from non-wetland covariates. The marginal R^2 is the variation in the response variable explained by the model fixed effects alone. The conditional R^2 is the variation explained by both fixed and random effects. RMSE = root mean squared error.

Response#	n	pctNRDeep	pctNRMid	pctNRShw	pctRiparian	Param Range	Marginal R^2	Conditional R^2	RMSE
				Acidification					
Al	1180	**-0.086 [0.058]**	0.056 [0.058]	**0.126 [0.044]**	**0.194 [0.133]***	-0.24 – 0.19	0.152	0.235	0.81
ANC	1788	**0.046 [0.026]**	-0.019 [0.032]	**-0.047 [0.026]**	**-0.086 [0.031]**	-0.36 – 0.31	0.6	0.674	0.48
pH	1764	**0.039 [0.032]**	**-0.049 [0.039]**	**-0.061 [0.032]**	-0.136 [0.158]*	-0.40 – 0.25	0.504	0.566	0.60
Ca	1787	**0.034 [0.029]**	-0.034 [0.158]*	-0.02 [0.028]	**-0.069 [0.035]**	-0.35 – 0.34	0.584	0.642	0.54
Mg	1787	**0.045 [0.029]**	-0.059 [0.151]*	-0.009 [0.028]	**-0.05 [0.035]**	-0.51 – 0.22	0.601	0.643	0.54
Cond	1764	**0.027 [0.025]**	-0.015 [0.03]	-0.01 [0.025]	**-0.076 [0.031]**	-0.45 – 0.22	0.668	0.693	0.47
				Brownification					
DOC	1788	**0.088 [0.033]**	**0.161 [0.041]**	**0.179 [0.087]***	**0.391 [0.187]***	-0.39 – 0.39	0.573	0.595	0.61
Color	1786	**0.056 [0.038]**	**0.146 [0.048]**	**0.188 [0.118]***	**0.442 [0.199]***	-0.13 – 0.44	0.426	0.461	0.75
				Eutrophication					
NO_3	1338	-0.047 [0.099]*	0.02 [0.114]*	-0.046 [0.072]*	-0.052 [0.061]	-0.52 – 0.19	0.394	0.397	0.70
				Sedimentation					
TSS	1694	-0.02 [0.041]	-0.021 [0.05]	-0.038 [0.043]	**-0.095 [0.049]**	-0.22 – -0.02	0.172	0.258	0.79
Turb	1764	-0.001 [0.039]	0.003 [0.048]	-0.031 [0.04]	-0.024 [0.047]	-0.24 – 0.12	0.255	0.324	0.75

#Al = aluminum, ANC = acid neutralizing capacity, Ca = calcium, Mg = magnesium, Cond = specific conductance, DOC = dissolved organic carbon, NO_3 = nitrate, TSS = total suspended sediments, Turb = turbidity.

*Indicates a regional random effect (i.e., significant regional variation) based on the likelihood ratio test (LRT).

Table S4. Standardized population mean regression slopes [confidence intervals] for all wetlands combined and model performances for four groups of stream constituents using linear mixed effects models. The slope represents the standardized relationship between the constituent and wetland averaged across all regions. Bold type indicates that the regression slope plus or minus the confidence interval (two times the standard error of the slope estimate) did not overlap with zero. Twenty-four physiographic regions (https://water.usgs.gov/GIS/metadata/usgswrd/XML/physio.xml) were used as the grouping factor for mixed effects modeling for all constituents except for NO_3, where the nine NRSA ecoregions[69] were used. Note that the importance of wetlands relative to other watershed metrics varied between constituents; full results for all covariates available in this Supplement as "Mixed Effects Models - Four Wetland Classes" and "Mixed Effects Models - Single Wetland Class". Param Range reports the range of standardized parameter values present in each model, including from non-wetland covariates. The marginal R^2 is the variation in the response variable explained by the model fixed effects alone. The conditional R^2 is the variation explained by both fixed and random effects. RMSE = root mean squared error.

**Response[#]**	**pctWetland**	**Param Range**	**Marginal R^2**	**Conditional R^2**	**RMSE**
			Acidification		
Al	**0.184 [0.136]***	-0.21 – 0.18	0.125	0.22	0.82
ANC	**-0.106 [0.032]**	-0.34 – 0.31	0.595	0.677	0.48
pH	-0.097 [0.167]*	-0.40 – 0.25	0.479	0.545	0.60
Ca	**-0.082 [0.036]**	-0.35 – 0.34	0.572	0.641	0.53
Mg	**-0.05 [0.036]**	-0.53 – 0.23	0.599	0.642	0.55
Cond	**-0.08 [0.03]**	-0.45 – 0.22	0.665	0.693	0.48
			Brownification		
DOC	**0.551 [0.156]***	-0.41 – 0.55	0.545	0.576	0.62
Color	**0.583 [0.161]***	-0.09 – 0.58	0.399	0.435	0.76
			Eutrophication		
NO_3	**-0.094 [0.056]**	-0.09 – 0.52	0.386	0.386	0.71
			Sedimentation		
TSS	**-0.131 [0.05]**	-0.22 – -0.05	0.173	0.258	0.79
Turb	-0.041 [0.048]	-0.24 – 0.12	0.256	0.323	0.75

[#]Al = aluminum, ANC = acid neutralizing capacity, Ca = calcium, Mg = magnesium, Cond = specific conductance, DOC = dissolved organic carbon, NO_3 = nitrate, TSS = total suspended sediments, Turb = turbidity.
[*]Indicates a regional random effect (i.e., significant regional variation) based on the likelihood ratio test (LRT).

Table S5. Wald chi-square tests for the fixed effects between pairwise comparisons of non-riparian wetland connectivity classes. Test determines whether the difference between the standardized population mean regression slopes (Table S3) of the two wetland classes is significantly different than zero (i.e., tests whether slope1 – slope2 = 0). Values are p-values, and **bold values** are significant at p = 0.05.

Constituent*	NRDeep–NRShw	NRDeep–NRMid	NRMid–NRShw
Acidification			
Al	**0.0000**	**0.0002**	**0.0456**
ANC	**0.0000**	**0.0013**	0.1486
pH	**0.0000**	**0.0003**	0.6415
Ca	**0.0056**	0.4020	0.8625
Mg	**0.0065**	0.1773	0.5082
Cond	**0.0320**	**0.0298**	0.7649
Brownification			
DOC	**0.0445**	**0.0036**	0.6899
Color	**0.0316**	**0.0029**	0.4990
Eutrophication			
NO_3	0.9945	0.3740	0.3167
Sedimentation			
TSS	0.5439	0.9698	0.6048
Turb	0.2692	0.8728	0.2400

*Al = aluminum, ANC = acid neutralizing capacity, Ca = calcium, Mg = magnesium, Cond = specific conductance, DOC = dissolved organic carbon, NO_3 = nitrate, TSS = total suspended sediments, Turb = turbidity.

REFERENCES

1. Tiner, R. W., Bergquist, H. C., DeAlessio, G. P. & Starr, M. J. Geographically isolated wetlands: a preliminary assessment of their characteristics and status in selected areas of the United States. (US Department of the Interior, Fish and Wildlife Service, Northeast Region, Hadley, MA, 2002).
2. Hobson, W. A. & Dahlgren, R. A. Soil forming processes in vernal pools of northern California, Chico area. in *Ecology, Conservation, and Management of Vernal Pool Ecosystems* (eds C. W. Witham *et al.*). Pp. 24-37 (California Native Plant Society, 1998).
3. Rains, M. C., Dahlgren, R. A., Fogg, G. E., Harter, T. & Williamson, R. J. Geological control of physical and chemical hydrology in California vernal pools. *Wetlands* **28**, 347-362 (2008).
4. Rains, M. C., Fogg, G. E., Harter, T., Dahlgren, R. A. & Williamson, R. J. The role of perched aquifers in hydrological connectivity and biogeochemical processes in vernal pool landscapes, Central Valley, California. *Hydrological Processes* **20**, 1157-1175 (2006).
5. Jokerst, J. D. Floristic analysis of volcanic mudflow vernal pools. in *Vernal pool plants: their habitat and biology. Studies from the Herbarium Number 8* (eds D. H. Ikeda & R. A. Schlising). Pp. 1-26 (California State University, 1990).
6. Weitkamp, W. A., Graham, R. C., Anderson, M. A. & Amrhein, C. Pedogenesis of a vernal pool Entisol-Alfisol-Vertisol catena in southern California. *Soil Science Society of America Journal* **60**, 316-323, doi:https://doi.org/10.2136/sssaj1996.03615995006000010048x (1996).
7. Smith, D. W. & Verrill, W. L. Vernal pool-soil-landform relationships in the Central Valley, California. in *Ecology, Conservation, and Management of Vernal Pool Ecosystems – Proceedings from a 1996 Conference* (eds C. W. Witham *et al.*). Pp. 15-23 (California Native Plant Society, 1998).
8. Winter, T. C. The concept of hydrologic landscapes. *Journal of the American Water Resources Association* **37**, 335-349 (2001).
9. Holland, R. F. Great Valley vernal pool distribution, photorevised 1996. in *Ecology, conservation, and management of vernal pool ecosystems* (eds C. W. Witham *et al.*). Pp. 71-75 (California Native Plants Society, 1998).
10. Bonada, N. & Resh, V. H. Mediterranean-climate streams and rivers: geographically separated but ecologically comparable freshwater systems. *Hydrobiologia* **719**, 1-29, doi:10.1007/s10750-013-1634-2 (2013).
11. Stepchinski, L. M. *et al.* Hydrologic connectivity and flow generation from California vernal pool, swale, and headwater stream complexes to downstream waters. *Wetlands* (in review).
12. Criss, R. E. & Davisson, M. L. Isotopic imaging of surface water/groundwater interactions, Sacramento Valley, California. *Journal of Hydrology* **178**, 205-222, doi:https://doi.org/10.1016/0022-1694(96)83733-4 (1996).
13. Fisk, H. N. Geological Investigation of the Alluvial Valley of the Lower Mississippi River. 78 pp. (Mississippi River Commission, Vicksburg, Mississippi, 1944).

14 Kroes, D. E. *et al.* Hydrologic modification and channel evolution degrades connectivity on the Atchafalaya River floodplain. *Earth Surface Processes and Landforms* **47**, 1790-1807, doi:https://doi.org/10.1002/esp.5347 (2022).
15 Gardiner, E. S. & Oliver, J. M. Restoration of bottomland hardwood forests in Lower Mississippi Aluvial Valey, U.S.A. in *Restoration of boreal and temperate forests.* (eds J. A. Stanturf & P. Madsen). Pp. 235-251 (CRC Press, 2005).
16 Patterson, W. B. *Vegetation, soils, and hydrology of central Louisiana bottomland hardwood forest types.* Ph.D. thesis, Louisiana State University, Baton Rouge, LA (1997).
17 Berkowitz, J. F., Johnson, D. R. & Price, J. J. Forested wetland hydrology in a large Mississippi River tributary system. *Wetlands* **40**, 1133-1148, doi:10.1007/s13157-019-01249-5 (2020).
18 Hudson, P. F., Sounny-Slitine, M. A. & LaFevor, M. A new longitudinal approach to assess hydrologic connectivity: Embanked floodplain inundation along the lower Mississippi River. *Hydrological Processes* **27**, 2187-2196, doi:https://doi.org/10.1002/hyp.9838 (2013).
19 Junk, W. J., Bayley, P. B. & Sparks, R. E. The flood pulse concept in river-floodplain systems. in *Proceedings of the International Large River Symposium Canadian Special Publication of Fisheries and Aquatic Sciences 106* (ed D. P. Dodge). Pp. 110-127 (Fisheries and Oceans Canada, 1989).
20 Haukos, D. A. & Smith, L. M. Past and future impacts of wetland regulations on playa ecology in the southern great plains. *Wetlands* **23**, 577-589, doi:10.1672/0277-5212(2003)023[0577:PAFIOW]2.0.CO;2 (2003).
21 Tiner, R. W. Geographically isolated wetlands of the United States. *Wetlands* **23**, 494-516 (2003).
22 Bolen, E. G., Smith, L. M. & Schramm, H. L., Jr. Playa lakes: prairie wetlands of the Southern High Plains. *Bioscience* **39**, 615-623, doi:10.2307/1311091 (1989).
23 Sublette, J. E. & Sublette, M. S. The limnology of playa lakes on the Llano Estacado, New Mexico and Texas. *The Southwestern Naturalist* **12**, 369–406 (1967).
24 Scanlon, B. *et al.* Evaluation of Enhanced Recharge Potential to the Ogallala Aquifer in the Brazos River Basin, Hale County, Texas. (Austin, Texas, 2003).
25 Brooks, E., Emel, J., Jokisch, B. & Robbins, P. *The Llano Estacado of the US Southern High Plains: Environmental Transformation and the Prospect for Sustainability*. (United Nations University Press, 2000).
26 Center for Geospatial Technology. Depth to Water Map. Geography of the Ogallala Aquifer in Texas, Texas Tech University. https://www.depts.ttu.edu/geospatial/center/Ogallala/Storymap/Index.html. (undated)
27 U.S. EPA. Level III and IV Ecoregions of the Continental United States. (US EPA National Health and Environmental Effects Research Laboratory, Western Ecology Division, Corvallis, OR, 2013).
28 Fretwell, J. D., J.S. Williams, and P.J. Redman (compilers). *National Water Summary on Wetland Resources*. (United States Government Printing Office, 1996).
29 Shepard, J. P. A statistical description of hydric soils of the Southeastern United States. (U.S. Department of Agriculture, U.S Forest Service, Southern Forest Experiment Station, New Orleans, Louisiana, United States, 1993).

30 Wharton, C. H., Kitchens, W. M., Pendleton, E. C. & Sipe, T. W. The Ecology of Bottomland Hardwood Swamps of the Southeast: A Community Profile. Report No. FWS/OBS-81/37, (US Department of the Interior, Washington, DC, 1982).
31 Prouty, W. F. Carolina Bays and their origin. *Geological Society of America Bulletin* **63**, 167-224 (1952).
32 Richardson, C. J. Pocosins: vanishing wastelands or valuable wetlands. *Bioscience* **33**, 626-633 (1983).
33 Sullivan, D. G., White, J. G. & Vepraskas, M. J. Using Land-Use Change, Soil Characteristics, and a Semi-Automated On-Line GIS Database to Inventory Carolina Bays. *Wetlands* **37**, 89-98, doi:10.1007/s13157-016-0842-8 (2017).
34 Bennett, S. H. & Nelson, J. B. Distribution and Status of Carolina Bays in South Carolina. (South Carolina Wildlife and Marine Resources Department, 1991).
35 Folkerts, G. W. Citronelle ponds: little-known wetlands of the central Gulf Coastal Plain, USA. *Natural Areas Journal* **17**, 6-16 (1997).
36 Fenstermacher, D. E., Rabenhorst, M. C., Lang, M. W., McCarty, G. W. & Needelman, B. A. Distribution, Morphometry, and Land Use of Delmarva Bays. *Wetlands* **34**, 1219-1228, doi:10.1007/s13157-014-0583-5 (2014).
37 Sharitz, R. R. & Gresham, C. A. Pocosins and Carolina Bays. in *Southern Forested Wetlands* (eds M.G. Messina & W.H. Conner). (Lewis Publishers, 1998).
38 Pyzoha, J. E., Callahan, T. J., Sun, G., Trettin, C. C. & Miwa, M. A conceptual hydrologic model for a forested Carolina bay depressional wetland on the Coastal Plain of South Carolina, USA. *Hydrological Processes* **22**, 2689-2698, doi:10.1002/hyp.6866 (2008).
39 U.S. EPA. Connectivity of Streams and Wetlands to Downstream Waters: A Review and Synthesis of the Scientific Evidence. 408 pp. (Office of Research and Development, U.S. Environmental Protection Agency, Washington, D.C., 2015).
40 LaBaugh, J. W. *et al.* Long-term changes in pond permanence, size, and salinity in Prairie Pothole Region wetlands: The role of groundwater-pond interaction. *Journal of Hydrology: Regional Studies* **17**, 1-23, doi:https://doi.org/10.1016/j.ejrh.2018.03.003 (2018).
41 Brannen, R., Spence, C. & Ireson, A. Influence of shallow groundwater–surface water interactions on the hydrological connectivity and water budget of a wetland complex. *Hydrological Processes* **29**, 3862-3877, doi:10.1002/hyp.10563 (2015).
42 Euliss, N. H. *et al.* The wetland continuum: a conceptual framework for interpreting biological studies. *Wetlands* **24**, 448-458 (2004).
43 Winter, T. C. & Rosenberry, D. O. The interaction of ground water with prairie pothole wetlands in the Cottonwood Lake area, east-central North Dakota, 1979–1990. *Wetlands* **15**, 193-211, doi:10.1007/bf03160700 (1995).
44 Neff, B. P. & Rosenberry, D. O. Groundwater connectivity of upland-embedded wetlands in the Prairie Pothole Region. *Wetlands* **38**, 51-63, doi:10.1007/s13157-017-0956-7 (2018).
45 van der Kamp, G. & Hayashi, M. Groundwater-wetland ecosystem interaction in the semiarid glaciated plains of North America. *Hydrogeology Journal* **17**, 203-214, doi:10.1007/s10040-008-0367-1 (2009).
46 Neff, B. P. *et al.* A hydrologic landscapes perspective on groundwater connectivity of depressional wetlands. *Water* **12**, 50, doi:doi:10.3390/w12010050 (2020).

47 Leibowitz, S. G., Mushet, D. M. & Newton, W. E. Intermittent surface water connectivity: fill and spill vs. fill and merge dynamics. *Wetlands* **36 (Suppl 2)**, S323-S342, doi:10.1007/s13157-016-0830-z (2016).
48 Leibowitz, S. G. & Vining, K. C. Temporal connectivity in a prairie pothole complex. *Wetlands* **23**, 13-25 (2003).
49 Shaw, D. A., Vanderkamp, G., Conly, F. M., Pietroniro, A. & Martz, L. The fill-spill hydrology of prairie wetland complexes during drought and deluge. *Hydrological Processes* **26**, 3147-3156, doi:10.1002/hyp.8390 (2012).
50 Vanderhoof, M. K., Christensen, J. R. & Alexander, L. C. Patterns and drivers for wetland connections in the Prairie Pothole Region, United States. *Wetlands Ecology and Management* **25**, 275-297, doi:10.1007/s11273-016-9516-9 (2017).
51 Brooks, J. R. *et al.* Estimating wetland connectivity to streams in the Prairie Pothole Region: an isotopic and remote sensing approach. *Water Resources Research* **54**, 955-977, doi:10.1002/2017WR021016 (2018).
52 Miller, B. A., Crumpton, W. G. & van der Valk, A. G. Wetland hydrologic class change from prior to European settlement to present on the Des Moines Lobe, Iowa. *Wetlands Ecology and Management* **20**, 1-8, doi:10.1007/s11273-011-9237-z (2012).
53 Natural Resources Conservation Service. Land resource regions and major land resource areas of the United States, the Caribbean, and the Pacific Basin. (U.S. Department of Agriculture, 2022).
54 Lane, C. R., D'Amico, E. & Autrey, B. Isolated wetlands of the southeastern United States: abundance and expected condition. *Wetlands* **32**, 753-767 (2012).
55 White, W. R. & Crisman, T. L. Headwater streams of Florida: types, distribution and a framework for conservation. *River Research and Applications* **32**, 452-461, doi:https://doi.org/10.1002/rra.2845 (2016).
56 Davis, S. M., and J.C. Ogden. *Everglades*. (St. Lucie Press, 1994).
57 Carlisle, V. W., and F.L. Moritz. *Florida soil identification handbook : thermic and hyperthermic temperature zones, including selected interpretations*. (Florida Cooperative Extension Service, University of Florida, Institute of Food and Agricultural Sciences, 1978).
58 Brown, R. B., Stone, E. L. & Carlisle, V. W. Soils. in *Ecosystems of Florida* (eds R. L. Myers & J. J. Ewel). Pp. 35-69 (University Presses of Florida, 1990).
59 Chen, E. & Gerber, J. F. Climate. in *Ecosystems of Florida* (eds R. L. Myers & J. J. Ewel). Pp. 11-34 (University Presses of Florida, 1990).
60 Nordlie, F. G. Rivers and springs. in *Ecosystems of Florida* (eds R. L. Myers & J. J. Ewel). Pp. 392-428 (University Presses of Florida, 1990).
61 Abrahamson, W. G. & Hartnett, D. C. Pine flatwoods and dry prairies. in *Ecosystems of Florida* (eds R. L. Myers & J. J. Ewel). Pp. 103-150 (University Presses of Florida, 1990).
62 Ewel, K. C. Swamps. in *Ecosystems of Florida* (eds R. L. Myers & J. J. Ewel). Pp. 281-323 (University Presses of Florida, 1990).
63 Kushlan, J. A. Freshwater marshes. in *Ecosystems of Florida* (eds R. L. Myers & J. J. Ewel). Pp. 323-363 (University Presses of Florida, 1990).
64 Brown, R. B., Stone, E. L. & Carlisle, W. W. Soils. in *Ecosystems of Florida* (eds R.M. Myers & J.J. Ewel). 35-69 (University Presses of Florida, 1990).

65 Louisiana Department of Wildlife and Fisheries. Plant Regions of Louisiana. https://www.wlf.louisiana.gov/assets/Resources/Publications/Plants_and_Natural_Communities/Native-Plant-Guide-Map.pdf. (undated)
66 U.S. EPA. National Rivers and Streams Assessment: Laboratory Methods Manual. Revision No. 1. (U.S. Environmental Protection Agency, Office of Water and Office of Research and Development, 2009).
67 Hill, R. A., Weber, M. H., Leibowitz, S. G., Olsen, A. R. & Thornbrugh, D. J. The Stream-Catchment (StreamCat) dataset: a database of watershed metrics for the conterminous United States. *Journal of the American Water Resources Association* **52**, 120-128, doi:10.1111/1752-1688.12372 (2016).
68 Bellmore, R. A. *et al.* Nitrogen inputs drive nitrogen concentrations in U.S. streams and rivers during summer low flow conditions. *Sci. Total Environ.* **639**, 1349-1359, doi:https://doi.org/10.1016/j.scitotenv.2018.05.008 (2018).
69 U.S. EPA. National Rivers and Streams Assessment 2008-2009: A Collaborative Survey. (U.S. Environmental Protection Agency, Office of Water and Office of Research and Development, Washington, D.C., 2016).

Mixed Effects Models - Four Wetland Classes

Introduction

- Code of mixed effects models used to assess the relative influence of four wetland classes on water quality
- Code:
    1. Aggregates water constituent data and watershed variables for modeling.
    2. Conducts mixed effects modeling with backwards selection.
    3. Presents models output, including model performance (e.g., marginal and conditional r-squared values), variable inflation factors (correlations among covariates), and model output (e.g., model parameters).

Get physiographic regions

- Physiographic regions of the conterminous US were obtained from: https://water.usgs.gov/GIS/metadata/usgswrd/XML/physio.xml
- Regions were used in models as grouping variable (random effect)
- Both Physiographic Divisions and Provinces were tested in preliminary models, but Provinces were ultimately selected

```
library(sf)
sf::sf_use_s2(FALSE)
physio <- st_read('./mixed-effects-model-data/physio.shp')[,
c('DIVISION','PROVINCE')]
pts <- read.csv('./mixed-effects-model-data/siteinfo.csv')[, c('SITE_ID',
'LAT_DD83',  'LON_DD83')]
pts <- st_as_sf(pts, coords = c("LON_DD83", "LAT_DD83"), crs =
st_crs(physio), agr = "constant")
pts <- st_join(pts, physio)
st_geometry(pts) <- NULL
```

Read and prep data

Several datasets were combined for modeling:

- Response variable: Water quality measurements from the 2008/2009 National Rivers and Streams Assessment.
- Where necessary, response variables were transformed to achieve normally distributed residual errors.
- Plots of residual errors vs. fitted values identified diagonal stripes in TSS, Al, and NO3, indicating inflation due to zeros or detection limits (censored values). Although techniques exist to model censored or zero-inflated data, we chose to remove these values for simplicity and to use the same statistical methods across all constituent types.

- Covariates:
- StreamCat data [Hill et al. 2016](#) - Watershed soils, chemical content of the lithology, atmospheric deposition, agriculture on erodible soils, and base flow index.
- [Belmore et al. 2018](#) - Watershed forest cover, impervious surfaces, % agricultural cover, watershed area, precipitation, point-source N input, agricultural N inputs, and instream substrate size.
- Percentage of watersheds comprised of each wetland class described in the main text: (1) riparian, (2) non-riparian shallow, (3) non-riparian mid depth, and (4) non-riparian deep.
- Both response and predictor variables are centered (mean=0) and scaled (SD=1) to facilitate comparison of regression coefficinets among models.

```
library(stringr)

#Get non-zero minimum / 10
min2 <- function(x){
  nzmin <- min(x[x > 0], na.rm = T) / 5
  return(nzmin)
}

#Read StreamCat predictor table and select just variables needed for
analysis.
sc <- read.csv('./mixed-effects-model-data/FINAL_TABLE.csv')
sc <- sc[,
c(c('SITE_ID','HydrlCondWs','CaOWs','BFIWs','CompStrgthWs','AgKffactWs',
               'SiO2Ws','Al2O3Ws','SWs','OmWs','K2OWs','MgOWs',
               'NWs','P2O5Ws','SN_2008Ws','Na2OWs','ClayWs'))]
#Read NRSA siteinfo table to get the 9 aggregated ecoregions
siteinfo <- read.csv('./mixed-effects-model-data/siteinfo.csv')[,
c('SITE_ID', 'AGGR_ECO9_2015')]
siteinfo <- merge(siteinfo, pts, by='SITE_ID')

# Read Rebecca Bellmore's original table (Bellmore et al. 2018)
# Includes several instream NRSA variables from Rebecca's paper,
# her point source estimates, and kg of nitrogen applied to the watershed.
# Select just columns needed for analysis.
covars <- read.csv('./mixed-effects-model-data/bellmore-covariates.csv')
covars$kgNperWsArea <- log(covars$AllKgNWs / covars$WsAreaHa) #Kg N per
watershed area (hectares)
covars <- subset(covars, select = -c(WsAreaHa,AllKgNWs))

# Read in table with wetland percentages & select just columns needed
wtarea <- read.csv('./mixed-effects-model-data/pct-wetland-types.csv')
wtarea$WsArea <- log(wtarea$WsArea)

# Read in NRSA chem data and select appropriate columns.
chem <- read.csv('./mixed-effects-model-data/chem.csv')
chem$NO3 <- chem$NO3 + chem$NO2
# Remove data below detection limit (different for 2008 and 2009)
chem$NO3 <- ifelse(chem$NO3 < 0.011 & chem$YEAR == 2008, NA,
                   ifelse(chem$NO3 < 0.005 & chem$YEAR == 2009, NA,
chem$NO3))
```

```
chemlist <- c('TSS','AL', 'CA', 'ANC', 'COLOR', 'COND', 'DOC', 'MG','NO3',
              'TURB','PHLAB')
chem <- chem[chem$VISIT_NO == 1, ] # Select measurement from first visit only
chem <- chem[, c('SITE_ID',chemlist)]

# Transform some chemistry variables based on exploratory modeling.
chem$PHLAB <- chem$PHLAB^4
chem$ANC <- log(chem$ANC + abs(min(chem$ANC, na.rm = T))+1)
chem$TSS[chem$TSS == 0] <- NA
chem$TSS <- log(chem$TSS + min2(chem$TSS))
chem$AL[chem$AL == 0] <- NA
chem$AL <- log(chem$AL + min2(chem$AL))
# Remove horizontal stripes from AL data (possible detection limits)
chem$AL[chem$AL < -8.04 & chem$AL > -8.4] <- NA
chem$AL[chem$AL < -5.293 & chem$AL > -5.295] <- NA
chem$CA <- log(chem$CA)
chem$COLOR <- log(chem$COLOR+1)
chem$COND <- log(chem$COND)
chem$DOC <- log(chem$DOC)
chem$MG <- log(chem$MG + min2(chem$MG))
chem$NO3 <- log(chem$NO3)
chem$TURB <- log(chem$TURB)

#Center (mean=0) and scale (SD=1) response data
chem[,2:ncol(chem)] <- scale(chem[,2:ncol(chem)])

# Merge predictor data into final  table
dat <- merge(wtarea, sc, by='SITE_ID')
dat <- merge(dat, covars, by='SITE_ID')
dat <- merge(siteinfo, dat, by='SITE_ID', all.x = F, all.y = T)
dat <- dat[!is.na(dat$PROVINCE), ]

#Center (mean=0) and scale (SD=1) response data
dat[,5:ncol(dat)] <- scale(dat[,5:ncol(dat)])
```

Define models

- Each model as 13-16 predictor variables that were selected based on previous work (e.g., Bellmore et al. 2018) or judgement of coauthors.
- Every model includes as predictor variables the percent (%) of each watershed composed of:
  - Riparian wetlands (pctRipar)
  - Non-riparian wetlands with shallow flow paths (pctNRShallow)
  - Non-riparian wetlands with mid-depth flow paths (pctNRMid)
  - Non-riparian wetlands with fill and spill and deep flow paths (pctNRDeep)
- Metadata for chemical constituents can be found here.

- Preliminary modeling tested National Aquatic Resources Survey Ecoregions (NARS) and USGS Physiographic Regions. Most models worked best with Physiographic Provinces, but NO3 worked best with the NARS Ecoregions.

```
mods <- c(
  'PHLAB ~ WsArea + pctRipar + pctNRShallow + pctNRMid + pctNRDeep + imp.ws +
          for.ws + prec + ag.ws + HydrlCondWs + CaOWs',
  'TSS ~ WsArea + pctRipar + pctNRShallow + pctNRMid + pctNRDeep + imp.ws +
          for.ws + prec + subsz + HydrlCondWs + CompStrgthWs + AgKffactWs +
BFIWs',
  'AL ~ WsArea + pctRipar + pctNRShallow + pctNRMid + pctNRDeep + imp.ws +
        for.ws + prec + subsz + ag.ws + HydrlCondWs + CompStrgthWs + Al2O3Ws
+ BFIWs',
  'CA ~ WsArea + pctRipar + pctNRShallow + pctNRMid + pctNRDeep + imp.ws +
          for.ws + prec + subsz + ag.ws + HydrlCondWs + CompStrgthWs + CaOWs
+ BFIWs',
  'ANC ~ WsArea + pctRipar + pctNRShallow + pctNRMid + pctNRDeep + imp.ws +
          for.ws + prec + subsz + ag.ws + HydrlCondWs + CompStrgthWs + CaOWs
+ BFIWs',
  'COLOR ~ WsArea + pctRipar + pctNRShallow + pctNRMid + pctNRDeep + imp.ws +
          for.ws + prec + subsz + pt.N.aw + ag.ws + kgNperWsArea +
HydrlCondWs + CompStrgthWs + BFIWs',
  'COND ~ WsArea + pctRipar + pctNRShallow + pctNRMid + pctNRDeep + imp.ws +
          for.ws + prec + subsz + ag.ws + HydrlCondWs + CompStrgthWs + CaOWs
+ SWs + BFIWs',
  'DOC ~ WsArea + pctRipar + pctNRShallow + pctNRMid + pctNRDeep + imp.ws +
          for.ws + prec + subsz + ag.ws + OmWs + BFIWs',
  'MG ~ WsArea + pctRipar + pctNRShallow + pctNRMid + pctNRDeep + imp.ws +
          for.ws + prec + subsz + ag.ws + HydrlCondWs + CompStrgthWs + MgOWs
+ BFIWs',
  'NO3 ~ pctRipar + pctNRShallow + pctNRMid + pctNRDeep + imp.ws +
          for.ws + prec + pt.N.aw + NWs + kgNperWsArea*BFIWs',
  'TURB ~ WsArea + pctRipar + pctNRShallow + pctNRMid + pctNRDeep + imp.ws +
          for.ws + prec + subsz + pt.N.aw + ag.ws + kgNperWsArea + BFIWs +
ClayWs + CompStrgthWs'

)

used.region <- c(
  'PROVINCE',
  'PROVINCE',
  'PROVINCE',
  'PROVINCE',
  'PROVINCE',
  'PROVINCE',
  'PROVINCE',
  'PROVINCE',
  'PROVINCE',
  'AGGR_ECO9_2015',
  'PROVINCE'
)
```

Mixed-effects models

- Loop through each constituent model.

- First, build the most complex model with `lme4:lmer`. First model contains all fixed effect and random slopes for each covariate with respect to the eco- or physiographic regions.
    - Use `||` to denote uncorrelated random effects.
    - Variable inflation factor of fixed effects across all models was <5.
- Next, use `lmerTest::step` to conduct backward selection on random effects, then fixed effects. Force the model to include fixed effects for pctRipar, pctNRShallow, pctNRMid, and pctNRDeep.
- Note: It is possible for a covariate to be included as a random effect (random slope) but be excluded as a fixed effect. In such cases, the mean slope was not different from zero, but the random slopes did vary by region such that their inclusion was necessary for the model.

```
library(lme4);library(lmerTest)

models <- list()
for(i in 1:length(mods)){
  # Get the name of the current response variable from formula
  response <- str_trim(str_split(mods[i], '~')[[1]][1])
  # Get the list of random slopes to test from the fixed effects formula
  randos <- str_trim(str_split(mods[i], '~')[[1]][2])
  # Select correct response variable, merge with predictors, remove NAs and
possible duplicates
  tmpchem <- chem[, c('SITE_ID', response)]
  tmpdat <- merge(dat, tmpchem, by='SITE_ID')
  tmpdat <- na.omit(tmpdat)
  tmpdat <- tmpdat[!duplicated(tmpdat),]
  # Construct model formula that includes all fixed effects and random slopes
  # for each fixed effect with respect to ecoregion or physiographic region
  mod <- paste0('lmer(', mods[i], ' + ',
                '(1 + ', randos, ' || ', used.region[i], '), data=tmpdat)')
  # Run model
  mod <- eval(parse(text=mod))
  # Use lmerTest::step to select reduced model
  mod.reduced <- get_model(step(mod, alpha.random = 0.05,
                               keep =
c('pctRipar','pctNRShallow','pctNRMid','pctNRDeep')))
  models[[i]] <- mod.reduced
}

saveRDS(models, './mixed-effects-model-data/model-objects-fixed-random-
reduced-2021.08.06.rds')
```

Model Results

```
library(jtools);library(performance)

models <- readRDS('./mixed-effects-model-data/model-objects-fixed-random-
reduced-2021.08.06.rds')

for(i in 1:length(models)){
```

```
  print('------------------------------------------------------------------
----------')

  print(names(models[[i]]@frame[1]))
  print('--------------------Model Performance-------------------')
  print(model_performance(models[[i]]))
  print('--------------------Variable Inflation Factor-------------------')
  print(car::vif(models[[i]]))
  print('--------------------Model Output-------------------')
  print(summ(models[[i]]))
}
## [1] "------------------------------------------------------------------
---------"
## [1] "PHLAB"
## [1] "--------------------Model Performance-------------------"
## # Indices of model performance
##
## AIC      |      BIC | R2 (cond.) | R2 (marg.) |   ICC |  RMSE | Sigma
## ---------------------------------------------------------------------
## 3532.089 | 3636.120 |      0.566 |      0.504 | 0.126 | 0.602 | 0.616
## [1] "--------------------Variable Inflation Factor-------------------"
##       WsArea     pctRipar pctNRShallow     pctNRMid    pctNRDeep
prec
##     1.025850     1.036127     1.011803     1.025130     1.031516
1.017308
##        ag.ws  HydrlCondWs        CaOWs
##     1.012686     1.020239     1.010642
## [1] "-------------------Model Output-------------------"
## MODEL INFO:
## Observations: 1764
## Dependent Variable: PHLAB
## Type: Mixed effects linear regression
##
## MODEL FIT:
## AIC = 3532.09, BIC = 3636.12
## Pseudo-R² (fixed effects) = 0.50
## Pseudo-R² (total) = 0.57
##
## FIXED EFFECTS:
## ------------------------------------------------------------
##                       Est.   S.E.   t val.      d.f.      p
## ------------------ ------- ------ -------- --------- ------
## (Intercept)           0.04   0.07     0.65     30.00   0.52
## WsArea                0.18   0.03     6.83     14.79   0.00
## pctRipar             -0.14   0.08    -1.72      5.40   0.14
## pctNRShallow         -0.06   0.02    -3.78   1689.62   0.00
## pctNRMid             -0.05   0.02    -2.54   1646.63   0.01
## pctNRDeep             0.04   0.02     2.42   1694.03   0.02
## prec                 -0.40   0.05    -7.49     18.43   0.00
## ag.ws                 0.14   0.04     3.47      9.62   0.01
## HydrlCondWs           0.06   0.02     3.28   1719.34   0.00
## CaOWs                 0.25   0.04     5.87     14.71   0.00
## ------------------------------------------------------------
##
## p values calculated using Satterthwaite d.f.
##
## RANDOM EFFECTS:
```

```
## --------------------------------------
##    Group       Parameter    Std. Dev.
## ------------ ------------- -----------
##   PROVINCE    (Intercept)     0.23
##  PROVINCE.1     WsArea        0.07
##  PROVINCE.2    pctRipar       0.20
##  PROVINCE.3     imp.ws        0.14
##  PROVINCE.4     for.ws        0.11
##  PROVINCE.5      prec         0.17
##  PROVINCE.6      ag.ws        0.09
##  PROVINCE.7      CaOWs        0.14
##   Residual                    0.62
## --------------------------------------
##
## Grouping variables:
## ----------------------------
##   Group     # groups   ICC
## ---------- ---------- ------
##  PROVINCE      24      0.10
## ----------------------------
## [1] "----------------------------------------------------------------------------
---------"
## [1] "TSS"
## [1] "-------------------Model Performance-------------------"
## # Indices of model performance
##
## AIC      |      BIC | R2 (cond.) | R2 (marg.) |   ICC |  RMSE | Sigma
## ---------------------------------------------------------------------
## 4233.135 | 4320.092 |      0.258 |      0.172 | 0.104 | 0.785 | 0.800
## [1] "-------------------Variable Inflation Factor--------------------"
##     pctRipar pctNRShallow     pctNRMid    pctNRDeep       for.ws
subsz
##     1.142558     1.090900     1.053223     1.036520     1.035560
1.031012
##  HydrlCondWs CompStrgthWs
##     1.207484     1.219798
## [1] "-------------------Model Output-------------------"
## MODEL INFO:
## Observations: 1694
## Dependent Variable: TSS
## Type: Mixed effects linear regression
##
## MODEL FIT:
## AIC = 4233.13, BIC = 4320.09
## Pseudo-R² (fixed effects) = 0.17
## Pseudo-R² (total) = 0.26
##
## FIXED EFFECTS:
## ------------------------------------------------------------
##                       Est.   S.E.   t val.      d.f.      p
## ------------------ ------- ------ -------- --------- ------
## (Intercept)          -0.13   0.08    -1.64     19.07   0.12
## pctRipar             -0.10   0.02    -3.87   1570.71   0.00
## pctNRShallow         -0.04   0.02    -1.77   1651.10   0.08
## pctNRMid             -0.02   0.03    -0.85   1467.89   0.40
## pctNRDeep            -0.02   0.02    -0.98   1584.78   0.33
## for.ws               -0.22   0.05    -4.32     24.44   0.00
```

```
## subsz                -0.20   0.02    -8.04   1681.46   0.00
## HydrlCondWs          -0.05   0.02    -2.23   1628.68   0.03
## CompStrgthWs         -0.09   0.03    -2.57   1422.30   0.01
## ------------------------------------------------------------
##
## p values calculated using Satterthwaite d.f.
##
## RANDOM EFFECTS:
## --------------------------------------
##    Group       Parameter    Std. Dev.
## ------------ ------------- -----------
##   PROVINCE    (Intercept)     0.27
##  PROVINCE.1     WsArea        0.14
##  PROVINCE.2     for.ws        0.15
##  PROVINCE.3      prec         0.19
##  PROVINCE.4   AgKffactWs      0.11
##  PROVINCE.5      BFIWs        0.12
##   Residual                    0.80
## --------------------------------------
##
## Grouping variables:
## ----------------------------
##   Group     # groups   ICC
## ---------- ---------- ------
##  PROVINCE      23      0.09
## ----------------------------
## [1] "----------------------------------------------------------------------------
---------"
## [1] "AL"
## [1] "-------------------Model Performance-------------------"
## # Indices of model performance
##
## AIC      |      BIC | R2 (cond.) | R2 (marg.) |   ICC |  RMSE | Sigma
## --------------------------------------------------------------------
## 3021.362 | 3087.315 |      0.235 |      0.152 | 0.098 | 0.812 | 0.826
## [1] "-------------------Variable Inflation Factor-------------------"
##       WsArea     pctRipar pctNRShallow     pctNRMid    pctNRDeep
for.ws
##     1.016681     1.106214     1.036115     1.058993     1.068930
1.060754
## CompStrgthWs
##     1.059613
## [1] "-------------------Model Output-------------------"
## MODEL INFO:
## Observations: 1180
## Dependent Variable: AL
## Type: Mixed effects linear regression
##
## MODEL FIT:
## AIC = 3021.36, BIC = 3087.31
## Pseudo-R² (fixed effects) = 0.15
## Pseudo-R² (total) = 0.24
##
## FIXED EFFECTS:
## ------------------------------------------------------------
##                        Est.   S.E.   t val.      d.f.      p
## ------------------ ------- ------ -------- --------- ------
```

```
## (Intercept)          -0.24   0.08    -3.17     23.18   0.00
## WsArea               -0.06   0.03    -2.20   1135.87   0.03
## pctRipar              0.19   0.07     2.91      4.31   0.04
## pctNRShallow          0.13   0.02     5.76   1130.60   0.00
## pctNRMid              0.06   0.03     1.94   1063.42   0.05
## pctNRDeep            -0.09   0.03    -2.99   1069.30   0.00
## for.ws                0.14   0.04     3.66    525.89   0.00
## CompStrgthWs          0.12   0.04     3.38    938.07   0.00
## ------------------------------------------------------------
##
## p values calculated using Satterthwaite d.f.
##
## RANDOM EFFECTS:
## --------------------------------------
##     Group       Parameter    Std. Dev.
## ------------ ------------- -----------
##    PROVINCE    (Intercept)     0.27
##   PROVINCE.1    pctRipar       0.12
##   PROVINCE.2     imp.ws        0.09
##   PROVINCE.3      prec         0.21
##    Residual                    0.83
## --------------------------------------
##
## Grouping variables:
## ----------------------------
##    Group     # groups   ICC
## ---------- ---------- ------
##   PROVINCE      24      0.09
## ----------------------------
## [1] "-----------------------------------------------------------------------
---------"
## [1] "CA"
## [1] "--------------------Model Performance-------------------"
## # Indices of model performance
##
## AIC      |      BIC | R2 (cond.) | R2 (marg.) |   ICC |  RMSE | Sigma
## ---------------------------------------------------------------------
## 3170.708 | 3291.450 |      0.642 |      0.584 | 0.139 | 0.526 | 0.540
## [1] "--------------------Variable Inflation Factor--------------------"
##        WsArea     pctRipar pctNRShallow     pctNRMid    pctNRDeep
imp.ws
##      1.047268     1.106163     1.070376     1.009235     1.031324
1.042197
##        for.ws         prec        subsz CompStrgthWs        CaOWs
##      1.114066     1.091206     1.032990     1.013942     1.006282
## [1] "--------------------Model Output-------------------"
## MODEL INFO:
## Observations: 1787
## Dependent Variable: CA
## Type: Mixed effects linear regression
##
## MODEL FIT:
## AIC = 3170.71, BIC = 3291.45
## Pseudo-R² (fixed effects) = 0.58
## Pseudo-R² (total) = 0.64
##
## FIXED EFFECTS:
```

```
## ----------------------------------------------------------
##                         Est.   S.E.   t val.      d.f.      p
## ------------------ ------- ------ -------- --------- ------
## (Intercept)           0.01   0.07     0.17     28.79   0.86
## WsArea                0.07   0.02     4.28   1748.55   0.00
## pctRipar             -0.07   0.02    -3.97   1625.73   0.00
## pctNRShallow         -0.02   0.01    -1.43   1681.30   0.15
## pctNRMid             -0.03   0.08    -0.43      6.47   0.68
## pctNRDeep             0.03   0.01     2.34   1644.74   0.02
## imp.ws                0.17   0.04     4.23     14.51   0.00
## for.ws               -0.16   0.04    -4.06     25.06   0.00
## prec                 -0.35   0.05    -6.65     18.13   0.00
## subsz                -0.05   0.02    -2.97   1754.14   0.00
## CompStrgthWs         -0.15   0.04    -3.43     11.11   0.01
## CaOWs                 0.34   0.04     8.61     14.68   0.00
## ----------------------------------------------------------
##
## p values calculated using Satterthwaite d.f.
##
## RANDOM EFFECTS:
## ----------------------------------------
##    Group       Parameter     Std. Dev.
## ------------ -------------- -----------
##   PROVINCE    (Intercept)      0.22
##  PROVINCE.1     pctNRMid       0.22
##  PROVINCE.2      imp.ws        0.12
##  PROVINCE.3      for.ws        0.11
##  PROVINCE.4       prec         0.16
##  PROVINCE.5      ag.ws         0.10
##  PROVINCE.6   CompStrgthWs     0.14
##  PROVINCE.7      CaOWs         0.13
##  PROVINCE.8      BFIWs         0.17
##   Residual                     0.54
## ----------------------------------------
##
## Grouping variables:
## ---------------------------
##   Group     # groups   ICC
## ---------- ---------- ------
##  PROVINCE      24      0.09
## ---------------------------
## [1] "----------------------------------------------------------------------
---------"
## [1] "ANC"
## [1] "-------------------Model Performance-------------------"
## # Indices of model performance
##
## AIC      |      BIC | R2 (cond.) | R2 (marg.) |   ICC |  RMSE | Sigma
## ---------------------------------------------------------------------
## 2834.177 | 2949.443 |      0.674 |      0.600 | 0.185 | 0.476 | 0.490
## [1] "-------------------Variable Inflation Factor-------------------"
##     pctRipar pctNRShallow     pctNRMid    pctNRDeep       imp.ws
for.ws
##     1.214204     1.088483     1.047055     1.041230     1.038828
1.165435
##         prec        subsz        ag.ws CompStrgthWs        CaOWs
##     1.067147     1.029424     1.218882     1.008158     1.005010
```

```
## [1] "-------------------Model Output-------------------"
## MODEL INFO:
## Observations: 1788
## Dependent Variable: ANC
## Type: Mixed effects linear regression
##
## MODEL FIT:
## AIC = 2834.18, BIC = 2949.44
## Pseudo-R² (fixed effects) = 0.60
## Pseudo-R² (total) = 0.67
##
## FIXED EFFECTS:
## -----------------------------------------------------------
##                       Est.   S.E.   t val.      d.f.      p
## ------------------ ------- ------ -------- --------- ------
## (Intercept)          -0.02   0.06    -0.24     27.35   0.81
## pctRipar             -0.09   0.02    -5.51   1733.13   0.00
## pctNRShallow         -0.05   0.01    -3.65   1677.07   0.00
## pctNRMid             -0.02   0.02    -1.18   1738.77   0.24
## pctNRDeep             0.05   0.01     3.58   1696.00   0.00
## imp.ws                0.09   0.03     3.11      9.55   0.01
## for.ws               -0.14   0.04    -3.29     26.66   0.00
## prec                 -0.36   0.05    -7.54     18.75   0.00
## subsz                -0.04   0.01    -2.88   1742.18   0.00
## ag.ws                 0.05   0.02     2.35   1263.54   0.02
## CompStrgthWs         -0.15   0.05    -2.79     16.40   0.01
## CaOWs                 0.31   0.04     7.26     17.53   0.00
## -----------------------------------------------------------
##
## p values calculated using Satterthwaite d.f.
##
## RANDOM EFFECTS:
## ---------------------------------------
##     Group       Parameter     Std. Dev.
## ------------ -------------- -----------
##   PROVINCE    (Intercept)      0.23
##  PROVINCE.1      WsArea        0.08
##  PROVINCE.2      imp.ws        0.08
##  PROVINCE.3      for.ws        0.15
##  PROVINCE.4       prec         0.14
##  PROVINCE.5   CompStrgthWs     0.20
##  PROVINCE.6      CaOWs         0.15
##  PROVINCE.7      BFIWs         0.15
##   Residual                     0.49
## ---------------------------------------
##
## Grouping variables:
## ----------------------------
##   Group     # groups   ICC
## ---------- ---------- ------
##  PROVINCE      24      0.12
## ----------------------------
## [1] "-------------------------------------------------------------------------
---------"
## [1] "COLOR"
## [1] "-------------------Model Performance-------------------"
## # Indices of model performance
```

```
##
## AIC      |      BIC | R2 (cond.) | R2 (marg.) |   ICC |  RMSE | Sigma
## -----------------------------------------------------------------------
## 4267.972 | 4361.263 |      0.461 |      0.426 | 0.061 | 0.746 | 0.759
## [1] "--------------------Variable Inflation Factor--------------------"
##     pctRipar pctNRShallow     pctNRMid    pctNRDeep         prec
subsz
##     1.047344     1.003745     1.047703     1.036918     1.022404
1.034475
##        ag.ws        BFIWs
##     1.048926     1.063338
## [1] "--------------------Model Output--------------------"
## MODEL INFO:
## Observations: 1786
## Dependent Variable: COLOR
## Type: Mixed effects linear regression
##
## MODEL FIT:
## AIC = 4267.97, BIC = 4361.26
## Pseudo-R² (fixed effects) = 0.43
## Pseudo-R² (total) = 0.46
##
## FIXED EFFECTS:
## ------------------------------------------------------------
##                        Est.   S.E.   t val.      d.f.      p
## ------------------ ------- ------ -------- --------- ------
## (Intercept)           0.02   0.06     0.25     25.36   0.81
## pctRipar              0.44   0.10     4.43      7.28   0.00
## pctNRShallow          0.19   0.06     3.20      1.04   0.19
## pctNRMid              0.15   0.02     6.07   1716.13   0.00
## pctNRDeep             0.06   0.02     2.91   1743.80   0.00
## prec                 -0.13   0.03    -4.17    502.07   0.00
## subsz                -0.10   0.02    -4.29   1745.60   0.00
## ag.ws                 0.13   0.04     3.07     74.80   0.00
## BFIWs                -0.11   0.03    -4.19   1069.85   0.00
## ------------------------------------------------------------
##
## p values calculated using Satterthwaite d.f.
##
## RANDOM EFFECTS:
## ---------------------------------------
##    Group       Parameter     Std. Dev.
## ------------ -------------- -----------
##   PROVINCE    (Intercept)      0.19
##  PROVINCE.1      WsArea        0.09
##  PROVINCE.2     pctRipar       0.26
##  PROVINCE.3   pctNRShallow     0.08
##  PROVINCE.4      for.ws        0.13
##  PROVINCE.5   kgNperWsArea     0.18
##  PROVINCE.6   HydrlCondWs      0.11
##   Residual                     0.76
## ---------------------------------------
##
## Grouping variables:
## ----------------------------
##   Group     # groups   ICC
## ---------- ---------- ------
```

```
##  PROVINCE      24      0.05
## ----------------------------
## [1] "------------------------------------------------------------------------
---------"
## [1] "COND"
## [1] "-------------------Model Performance-------------------"
## # Indices of model performance
##
## AIC      |      BIC | R2 (cond.) | R2 (marg.) |   ICC |  RMSE | Sigma
## ---------------------------------------------------------------------
## 2751.098 | 2871.556 |      0.693 |      0.668 | 0.077 | 0.474 | 0.487
## [1] "-------------------Variable Inflation Factor-------------------"
##       WsArea     pctRipar pctNRShallow     pctNRMid    pctNRDeep
imp.ws
##     1.022984     1.237177     1.109360     1.123147     1.028865
1.059511
##       for.ws         prec        subsz CompStrgthWs        CaOWs
BFIWs
##     1.187148     1.090458     1.042136     1.032597     1.007491
1.153649
## [1] "-------------------Model Output-------------------"
## MODEL INFO:
## Observations: 1764
## Dependent Variable: COND
## Type: Mixed effects linear regression
##
## MODEL FIT:
## AIC = 2751.10, BIC = 2871.56
## Pseudo-R² (fixed effects) = 0.67
## Pseudo-R² (total) = 0.69
##
## FIXED EFFECTS:
## ----------------------------------------------------------
##                       Est.   S.E.   t val.      d.f.      p
## ------------------ ------- ------ -------- --------- ------
## (Intercept)          -0.06   0.05    -1.30     27.94   0.20
## WsArea                0.06   0.02     2.67     15.36   0.02
## pctRipar             -0.08   0.02    -5.00   1657.51   0.00
## pctNRShallow         -0.01   0.01    -0.76   1685.94   0.45
## pctNRMid             -0.02   0.02    -1.01   1686.44   0.31
## pctNRDeep             0.03   0.01     2.16   1689.68   0.03
## imp.ws                0.22   0.03     6.50     16.63   0.00
## for.ws               -0.14   0.02    -5.93    458.73   0.00
## prec                 -0.45   0.05    -8.65     26.03   0.00
## subsz                -0.06   0.01    -3.74   1714.59   0.00
## CompStrgthWs         -0.11   0.03    -3.42      9.13   0.01
## CaOWs                 0.20   0.03     6.47     11.94   0.00
## BFIWs                -0.14   0.02    -8.37   1458.13   0.00
## ----------------------------------------------------------
##
## p values calculated using Satterthwaite d.f.
##
## RANDOM EFFECTS:
## --------------------------------------
##    Group       Parameter     Std. Dev.
## ------------ -------------- -----------
##   PROVINCE    (Intercept)      0.14
```

```
##  PROVINCE.1      WsArea        0.07
##  PROVINCE.2      imp.ws        0.10
##  PROVINCE.3       prec         0.19
##  PROVINCE.4      ag.ws         0.08
##  PROVINCE.5   CompStrgthWs     0.09
##  PROVINCE.6      CaOWs         0.09
##  PROVINCE.7       SWs          0.31
##   Residual                     0.49
## ---------------------------------------
##
## Grouping variables:
## ----------------------------
##   Group     # groups   ICC
## ---------- ---------- ------
##  PROVINCE      24      0.05
## ----------------------------
## [1] "-------------------------------------------------------------------------
---------"
## [1] "DOC"
## [1] "-------------------Model Performance-------------------"
## # Indices of model performance
##
## AIC      |      BIC | R2 (cond.) | R2 (marg.) |   ICC |  RMSE | Sigma
## ----------------------------------------------------------------------
## 3616.670 | 3715.469 |      0.595 |      0.573 | 0.051 | 0.610 | 0.623
## [1] "-------------------Variable Inflation Factor--------------------"
##     pctRipar pctNRShallow     pctNRMid    pctNRDeep       imp.ws
prec
##     1.029164     1.005503     1.055183     1.029769     1.015055
1.020184
##        subsz        BFIWs
##     1.019650     1.046326
## [1] "-------------------Model Output-------------------"
## MODEL INFO:
## Observations: 1788
## Dependent Variable: DOC
## Type: Mixed effects linear regression
##
## MODEL FIT:
## AIC = 3616.67, BIC = 3715.47
## Pseudo-R² (fixed effects) = 0.57
## Pseudo-R² (total) = 0.60
##
## FIXED EFFECTS:
## -------------------------------------------------------------
##                       Est.   S.E.   t val.      d.f.      p
## ------------------ ------- ------ -------- --------- ------
## (Intercept)          -0.04   0.06    -0.75     18.06   0.46
## pctRipar              0.39   0.09     4.19      3.43   0.02
## pctNRShallow          0.18   0.04     4.13      1.45   0.09
## pctNRMid              0.16   0.02     7.84   1702.74   0.00
## pctNRDeep             0.09   0.02     5.33   1672.81   0.00
## imp.ws                0.09   0.02     4.18    559.43   0.00
## prec                 -0.39   0.06    -6.41     21.57   0.00
## subsz                -0.13   0.02    -7.07   1744.47   0.00
## BFIWs                -0.27   0.02   -13.00   1146.73   0.00
## -------------------------------------------------------------
```

```
##
## p values calculated using Satterthwaite d.f.
##
## RANDOM EFFECTS:
## ---------------------------------------
##     Group       Parameter     Std. Dev.
## ------------ -------------- -----------
##   PROVINCE    (Intercept)      0.14
##  PROVINCE.1      WsArea        0.09
##  PROVINCE.2     pctRipar       0.25
##  PROVINCE.3   pctNRShallow     0.06
##  PROVINCE.4      for.ws        0.14
##  PROVINCE.5       prec         0.22
##  PROVINCE.6      ag.ws         0.24
##  PROVINCE.7       OmWs         0.21
##   Residual                     0.62
## ---------------------------------------
##
## Grouping variables:
## ----------------------------
##   Group     # groups   ICC
## ---------- ---------- ------
##  PROVINCE      24      0.03
## ----------------------------
## [1] "----------------------------------------------------------------------------------"
## [1] "MG"
## [1] "-------------------Model Performance-------------------"
## # Indices of model performance
##
## AIC      |      BIC | R2 (cond.) | R2 (marg.) |   ICC |  RMSE | Sigma
## ---------------------------------------------------------------------
## 3233.959 | 3360.190 |      0.643 |      0.601 | 0.107 | 0.538 | 0.553
## [1] "-------------------Variable Inflation Factor--------------------"
##       WsArea     pctRipar pctNRShallow     pctNRMid    pctNRDeep
imp.ws
##     1.050684     1.111146     1.087337     1.012558     1.030935
1.030774
##         prec        subsz        ag.ws CompStrgthWs        MgOWs
BFIWs
##     1.047916     1.023322     1.038986     1.046034     1.026066
1.032075
## [1] "-------------------Model Output-------------------"
## MODEL INFO:
## Observations: 1787
## Dependent Variable: MG
## Type: Mixed effects linear regression
##
## MODEL FIT:
## AIC = 3233.96, BIC = 3360.19
## Pseudo-R² (fixed effects) = 0.60
## Pseudo-R² (total) = 0.64
##
## FIXED EFFECTS:
## -------------------------------------------------------------
##                         Est.   S.E.   t val.      d.f.      p
## ----------------- ------- ------ -------- --------- ------
```

```
## (Intercept)          -0.09   0.06    -1.46     31.92   0.15
## WsArea                0.04   0.02     2.43   1748.10   0.02
## pctRipar             -0.05   0.02    -2.84   1655.55   0.00
## pctNRShallow         -0.01   0.01    -0.61   1682.44   0.54
## pctNRMid             -0.06   0.08    -0.79      4.67   0.47
## pctNRDeep             0.04   0.01     3.08   1682.35   0.00
## imp.ws                0.13   0.03     4.10     11.40   0.00
## prec                 -0.51   0.06    -9.15     22.03   0.00
## subsz                -0.06   0.03    -2.36     15.53   0.03
## ag.ws                 0.18   0.06     2.90     11.56   0.01
## CompStrgthWs         -0.13   0.02    -5.30   1497.30   0.00
## MgOWs                 0.22   0.03     7.05     20.15   0.00
## BFIWs                -0.13   0.03    -3.97      8.83   0.00
## -----------------------------------------------------------
##
## p values calculated using Satterthwaite d.f.
##
## RANDOM EFFECTS:
## --------------------------------------
##    Group       Parameter    Std. Dev.
## ------------ ------------- -----------
##   PROVINCE    (Intercept)     0.19
##  PROVINCE.1    pctNRMid       0.21
##  PROVINCE.2     imp.ws        0.08
##  PROVINCE.3     for.ws        0.13
##  PROVINCE.4      prec         0.19
##  PROVINCE.5      subsz        0.07
##  PROVINCE.6      ag.ws        0.19
##  PROVINCE.7      MgOWs        0.10
##  PROVINCE.8      BFIWs        0.08
##   Residual                    0.55
## --------------------------------------
##
## Grouping variables:
## ----------------------------
##   Group     # groups   ICC
## ---------- ---------- ------
##  PROVINCE      24      0.07
## ----------------------------
## [1] "-----------------------------------------------------------------------------
---------"
## [1] "NO3"
## [1] "-------------------Model Performance-------------------"
## # Indices of model performance
##
## AIC      |      BIC | R2 (cond.) | R2 (marg.) |   ICC |  RMSE | Sigma
## ---------------------------------------------------------------------
## 3077.656 | 3181.634 |      0.397 |      0.394 | 0.005 | 0.703 | 0.716
## [1] "-------------------Variable Inflation Factor-------------------"
##          pctRipar       pctNRShallow           pctNRMid
pctNRDeep
##          1.144707           1.039303           1.078895
1.012216
##            imp.ws               prec       kgNperWsArea
BFIWs
##          1.036899           1.028636           1.009162
1.072258
```

```
## kgNperWsArea:BFIWs
##            1.011677
## [1] "-------------------Model Output-------------------"
## MODEL INFO:
## Observations: 1338
## Dependent Variable: NO3
## Type: Mixed effects linear regression
##
## MODEL FIT:
## AIC = 3077.66, BIC = 3181.63
## Pseudo-R² (fixed effects) = 0.39
## Pseudo-R² (total) = 0.40
##
## FIXED EFFECTS:
## ----------------------------------------------------------------
##                              Est.   S.E.   t val.     d.f.      p
## ------------------------ ------- ------ -------- --------- ------
## (Intercept)                -0.16   0.05    -3.18   121.19   0.00
## pctRipar                   -0.05   0.03    -1.69   663.06   0.09
## pctNRShallow               -0.05   0.04    -1.29     1.03   0.41
## pctNRMid                    0.02   0.06     0.36     3.65   0.74
## pctNRDeep                  -0.05   0.05    -0.95     2.78   0.42
## imp.ws                      0.16   0.03     6.31   303.80   0.00
## prec                       -0.19   0.08    -2.50     6.51   0.04
## kgNperWsArea                0.52   0.08     6.69     6.17   0.00
## BFIWs                       0.09   0.03     3.01   234.66   0.00
## kgNperWsArea:BFIWs          0.15   0.05     3.18     6.28   0.02
## ----------------------------------------------------------------
##
## p values calculated using Satterthwaite d.f.
##
## RANDOM EFFECTS:
## ---------------------------------------------------
##       Group            Parameter       Std. Dev.
## ------------------ -------------------- -----------
##   AGGR_ECO9_2015       pctNRShallow        0.04
##  AGGR_ECO9_2015.1        pctNRMid          0.09
##  AGGR_ECO9_2015.2       pctNRDeep          0.10
##  AGGR_ECO9_2015.3         for.ws           0.10
##  AGGR_ECO9_2015.4          prec            0.19
##  AGGR_ECO9_2015.5        pt.N.aw           0.21
##  AGGR_ECO9_2015.6          NWs             0.17
##  AGGR_ECO9_2015.7      kgNperWsArea        0.21
##  AGGR_ECO9_2015.8   kgNperWsArea:BFIWs     0.11
##      Residual                              0.72
## ---------------------------------------------------
##
## Grouping variables:
## ----------------------------------
##      Group        # groups   ICC
## ---------------- ---------- ------
##  AGGR_ECO9_2015      9       0.00
## ----------------------------------
## [1] "--------------------------------------------------------------------
---------"
## [1] "TURB"
## [1] "-------------------Model Performance-------------------"
```

```
## # Indices of model performance
##
## AIC      |      BIC | R2 (cond.) | R2 (marg.) |   ICC |  RMSE | Sigma
## ---------------------------------------------------------------------
## 4258.862 | 4351.942 |      0.324 |      0.255 | 0.092 | 0.753 | 0.767
## [1] "--------------------Variable Inflation Factor--------------------"
##     pctRipar pctNRShallow     pctNRMid    pctNRDeep       for.ws
prec
##     1.220738     1.107478     1.079687     1.027399     1.205468
1.141834
##        subsz kgNperWsArea       ClayWs
##     1.028267     1.227111     1.070403
## [1] "-------------------Model Output-------------------"
## MODEL INFO:
## Observations: 1764
## Dependent Variable: TURB
## Type: Mixed effects linear regression
##
## MODEL FIT:
## AIC = 4258.86, BIC = 4351.94
## Pseudo-R² (fixed effects) = 0.25
## Pseudo-R² (total) = 0.32
##
## FIXED EFFECTS:
## ------------------------------------------------------------
##                       Est.   S.E.   t val.      d.f.      p
## ------------------ ------- ------ -------- --------- ------
## (Intercept)          -0.08   0.07    -1.18     15.02   0.26
## pctRipar             -0.02   0.02    -1.01   1724.11   0.31
## pctNRShallow         -0.03   0.02    -1.56   1716.22   0.12
## pctNRMid              0.00   0.02     0.15   1572.89   0.88
## pctNRDeep            -0.00   0.02    -0.07   1639.76   0.94
## for.ws               -0.10   0.04    -2.19     39.81   0.03
## prec                 -0.19   0.06    -3.36     15.53   0.00
## subsz                -0.24   0.04    -6.45     13.80   0.00
## kgNperWsArea          0.09   0.03     2.67    690.85   0.01
## ClayWs                0.12   0.03     4.38   1313.85   0.00
## ------------------------------------------------------------
##
## p values calculated using Satterthwaite d.f.
##
## RANDOM EFFECTS:
## --------------------------------------
##    Group       Parameter    Std. Dev.
## ------------ ------------- -----------
##   PROVINCE    (Intercept)     0.24
##  PROVINCE.1     WsArea        0.11
##  PROVINCE.2     for.ws        0.12
##  PROVINCE.3      prec         0.14
##  PROVINCE.4      subsz        0.12
##  PROVINCE.5      BFIWs        0.15
##   Residual                    0.77
## --------------------------------------
##
## Grouping variables:
## ----------------------------
##   Group     # groups   ICC
```

```
## ---------- ---------- ------
##  PROVINCE      24      0.08
## ----------------------------
```

Mixed Effects Models - Single Wetland Class

Introduction

- Code of mixed effects models used to assess the relative influence of wetlands (single class) on water quality
- Code:
    1. Aggregates water constituent data and watershed variables for modeling.
    2. Conducts mixed effects modeling with backwards selection.
    3. Presents models output, including model performance (e.g., marginal and conditional r-squared values), variable inflation factors (correlations among covariates), and model output (e.g., model parameters).

Get physiographic regions

- Physiographic regions of the conterminous US were obtained from: https://water.usgs.gov/GIS/metadata/usgswrd/XML/physio.xml
- Regions were used in models as grouping variable (random effect)
- Both Physiographic Divisions and Provinces were tested in preliminary models, but Provinces were ultimately selected

```
library(sf)
sf::sf_use_s2(FALSE)
physio <- st_read('./mixed-effects-model-data/physio.shp')[,
c('DIVISION','PROVINCE')]
pts <- read.csv('./mixed-effects-model-data/siteinfo.csv')[, c('SITE_ID',
'LAT_DD83',  'LON_DD83')]
pts <- st_as_sf(pts, coords = c("LON_DD83", "LAT_DD83"), crs =
st_crs(physio), agr = "constant")
pts <- st_join(pts, physio)
st_geometry(pts) <- NULL
```

Read and prepare data

Several datasets were combined for modeling:

- Response variable: Water quality measurements from the 2008/2009 National Rivers and Streams Assessment.
- Where necessary, response variables were transformed to achieve normally distributed residual errors.
- Plots of residual errors vs. fitted values identified diagonal stripes in TSS, Al, and NO3, indicating inflation due to zeros or detection limits (censored values). Although techniques exist to model censored or zero-inflated data, we chose to remove these values for simplicity and to use the same statistical methods across all constituent types.

- Covariates:
- StreamCat data Hill et al. 2016 - Watershed soils, chemical content of the lithology, atmospheric deposition, agriculture on erodible soils, and base flow index.
- Belmore et al. 2018 - Watershed forest cover, impervious surfaces, % agricultural cover, watershed area, precipitation, point-source N input, agricultural N inputs, and instream substrate size.
- Percentage of watersheds comprised of wetlands (no connectivity classes)
- Both response and predictor variables are centered (mean=0) and scaled (SD=1) to facilitate comparison of regression coefficinets among models.

```
library(stringr)

#Get non-zero minimum / 10
min2 <- function(x){
  nzmin <- min(x[x > 0], na.rm = T) / 5
  return(nzmin)
}

#Read StreamCat predictor table and select just variables needed for
analysis.
sc <- read.csv('./mixed-effects-model-data/FINAL_TABLE.csv')
sc <- sc[,
c(c('SITE_ID','HydrlCondWs','CaOWs','BFIWs','CompStrgthWs','AgKffactWs',
               'SiO2Ws','Al2O3Ws','SWs','OmWs','K2OWs','MgOWs',
               'NWs','P2O5Ws','SN_2008Ws','Na2OWs','ClayWs'))]
#Read NRSA siteinfo table to get the 9 aggregated ecoregions
siteinfo <- read.csv('./mixed-effects-model-data/siteinfo.csv')[,
c('SITE_ID', 'AGGR_ECO9_2015')]
siteinfo <- merge(siteinfo, pts, by='SITE_ID')

# Read Rebecca Bellmore's original table (Bellmore et al. 2018)
# Includes several instream NRSA variables from Rebecca's paper,
# her point source estimates, and kg of nitrogen applied to the watershed.
# Select just columns needed for analysis.
covars <- read.csv('./mixed-effects-model-data/bellmore-covariates.csv')
covars$kgNperWsArea <- log(covars$AllKgNWs / covars$WsAreaHa) #Kg N per
watershed area (hectares)
covars <- subset(covars, select = -c(WsAreaHa,AllKgNWs))

# Read in table with wetland percentages & select just columns needed
wtarea <- read.csv('./mixed-effects-model-data/pct-wetland-types.csv')
wtarea$WsArea <- log(wtarea$WsArea)
#Create aggregate wetland metric by combining all classes
wtarea$pctWetland <- rowSums(wtarea[,3:ncol(wtarea)])

# Read in NRSA chem data and select appropriate columns.
chem <- read.csv('./mixed-effects-model-data/chem.csv')
chem$NO3 <- chem$NO3 + chem$NO2
# Remove data below detection limit (different for 2008 and 2009)
chem$NO3 <- ifelse(chem$NO3 < 0.011 & chem$YEAR == 2008, NA,
                   ifelse(chem$NO3 < 0.005 & chem$YEAR == 2009, NA,
chem$NO3))
```

```
chemlist <- c('TSS','AL', 'CA', 'ANC', 'COLOR', 'COND', 'DOC', 'MG','NO3',
              'TURB','PHLAB')
chem <- chem[chem$VISIT_NO == 1, ] # Select measurement from first visit only
chem <- chem[, c('SITE_ID',chemlist)]

# Transform some chemistry variables based on exploratory modeling.
chem$PHLAB <- chem$PHLAB^4
chem$ANC <- log(chem$ANC + abs(min(chem$ANC, na.rm = T))+1)
chem$TSS[chem$TSS == 0] <- NA
chem$TSS <- log(chem$TSS + min2(chem$TSS))
chem$AL[chem$AL == 0] <- NA
chem$AL <- log(chem$AL + min2(chem$AL))
# Remove horizontal stripes from AL data (possible detection limits)
chem$AL[chem$AL < -8.04 & chem$AL > -8.4] <- NA
chem$AL[chem$AL < -5.293 & chem$AL > -5.295] <- NA
chem$CA <- log(chem$CA)
chem$COLOR <- log(chem$COLOR+1)
chem$COND <- log(chem$COND)
chem$DOC <- log(chem$DOC)
chem$MG <- log(chem$MG + min2(chem$MG))
chem$NO3 <- log(chem$NO3)
chem$TURB <- log(chem$TURB)

#Center (mean=0) and scale (SD=1) response data
chem[,2:ncol(chem)] <- scale(chem[,2:ncol(chem)])

# Merge predictor data into final  table
dat <- merge(wtarea, sc, by='SITE_ID')
dat <- merge(dat, covars, by='SITE_ID')
dat <- merge(siteinfo, dat, by='SITE_ID', all.x = F, all.y = T)
dat <- dat[!is.na(dat$PROVINCE), ]

#Center (mean=0) and scale (SD=1) response data
dat[,5:ncol(dat)] <- scale(dat[,5:ncol(dat)])
```

Define models

- Each model as 13-16 predictor variables that were selected based on previous work (e.g., Bellmore et al. 2018) or judgement of coauthors.
- Every model includes as predictor variables the percent (%) of each watershed composed of wetlands (i.e., no classes)
- Metadata for chemical constituents can be found here.
- Preliminary modeling tested National Aquatic Resources Survey Ecoregions (NARS) and USGS Physiographic Regions. Most models worked best with Physiographic Provinces, but NO3 worked best with the NARS Ecoregions.

```
mods <- c(
  'PHLAB ~ WsArea + pctWetland + imp.ws +
          for.ws + prec + ag.ws + HydrlCondWs + CaOWs',
  'TSS ~ WsArea + pctWetland + imp.ws +
          for.ws + prec + subsz + HydrlCondWs + CompStrgthWs + AgKffactWs +
BFIWs',
```

```
  'AL ~ WsArea + pctWetland + imp.ws +
        for.ws + prec + subsz + ag.ws + HydrlCondWs + CompStrgthWs + Al2O3Ws
+ BFIWs',
  'CA ~ WsArea + pctWetland + imp.ws +
          for.ws + prec + subsz + ag.ws + HydrlCondWs + CompStrgthWs + CaOWs
+ BFIWs',
  'ANC ~ WsArea + pctWetland + imp.ws +
          for.ws + prec + subsz + ag.ws + HydrlCondWs + CompStrgthWs + CaOWs
+ BFIWs',
  'COLOR ~ WsArea + pctWetland + imp.ws +
          for.ws + prec + subsz + pt.N.aw + ag.ws + kgNperWsArea +
HydrlCondWs + CompStrgthWs + BFIWs',
  'COND ~ WsArea + pctWetland + imp.ws +
          for.ws + prec + subsz + ag.ws + HydrlCondWs + CompStrgthWs + CaOWs
+ SWs + BFIWs',
  'DOC ~ WsArea + pctWetland + imp.ws +
          for.ws + prec + subsz + ag.ws + OmWs + BFIWs',
  'MG ~ WsArea + pctWetland + imp.ws +
          for.ws + prec + subsz + ag.ws + HydrlCondWs + CompStrgthWs + MgOWs
+ BFIWs',
  'NO3 ~ pctWetland + imp.ws +
          for.ws + prec + pt.N.aw + NWs + kgNperWsArea*BFIWs',
  'TURB ~ WsArea + pctWetland + imp.ws +
          for.ws + prec + subsz + pt.N.aw + ag.ws + kgNperWsArea + BFIWs +
ClayWs + CompStrgthWs'

)

used.region <- c(
  'PROVINCE',
  'PROVINCE',
  'PROVINCE',
  'PROVINCE',
  'PROVINCE',
  'PROVINCE',
  'PROVINCE',
  'PROVINCE',
  'PROVINCE',
  'AGGR_ECO9_2015',
  'PROVINCE'
)
```

Mixed-effects models

- Loop through each constituent model.
- First, build the most complex model with `lme4:lmer`. First model contains all fixed effect and random slopes for each covariate with respect to the eco- or physiographic regions.
- Use `||` to denote uncorrelated random effects. Variable inflation factor of fixed effects across all models was <5.

- Next, use `lmerTest::step` to conduct backward selection on random effects, then fixed effects. Force the model to include fixed effects for pctRipar, pctNRShallow, pctNRMid, and pctNRDeep.
- Note: It is possible for a covariate to be included as a random effect (random slope) but be excluded as a fixed effect. In such cases, the mean slope was not different from zero, but the random slopes did vary by region such that their inclusion was necessary for the model.

```
library(lme4);library(lmerTest)

models <- list()
for(i in 1:length(mods)){
  # Get the name of the current response variable from formula
  response <- str_trim(str_split(mods[i], '~')[[1]][1])
  # Get the list of random slopes to test from the fixed effects formula
  randos <- str_trim(str_split(mods[i], '~')[[1]][2])
  # Select correct response variable, merge with predictors, remove NAs and
possible duplicates
  tmpchem <- chem[, c('SITE_ID', response)]
  tmpdat <- merge(dat, tmpchem, by='SITE_ID')
  tmpdat <- na.omit(tmpdat)
  tmpdat <- tmpdat[!duplicated(tmpdat),]
  print(length(unique(tmpdat$PROVINCE)))
  # Construct model formula that includes all fixed effects and random slopes
for each fixed effect with respect to ecoregion or physiographic region
  mod <- paste0('lmer(', mods[i], ' + ',
                '(1 + ', randos, ' || ', used.region[i], '), data=tmpdat)')
  # Run model
  mod <- eval(parse(text=mod))
  # Use lmerTest::step to select reduced model
  mod.reduced <- get_model(step(mod, alpha.random = 0.05,
                               keep = c('pctWetland')))
  models[[i]] <- mod.reduced
}

saveRDS(models, './mixed-effects-model-data/full-wetlands-models-
2021.08.06.rds')
```

Model Results

```
library(jtools);library(performance)

models <- readRDS('./mixed-effects-model-data/full-wetlands-models-
2021.08.06.rds')

for(i in 1:length(models)){
  print('-----------------------------------------------------------------
----------')

  print(names(models[[i]]@frame[1]))
  print('--------------------Model Performance--------------------')
  print(model_performance(models[[i]]))
  print('--------------------Variable Inflation Factor--------------------')
  print(car::vif(models[[i]]))
  print('--------------------Model Output--------------------')
```

```
  print(summ(models[[i]]))
}
## [1] "------------------------------------------------------------------------
---------"
## [1] "PHLAB"
## [1] "--------------------Model Performance-------------------"
## # Indices of model performance
##
## AIC      |      BIC | R2 (cond.) | R2 (marg.) |   ICC |  RMSE | Sigma
## ---------------------------------------------------------------------
## 3519.072 | 3606.678 |      0.545 |      0.479 | 0.127 | 0.603 | 0.617
## [1] "--------------------Variable Inflation Factor--------------------"
##      WsArea  pctWetland        prec       ag.ws HydrlCondWs       CaOWs
##    1.022713    1.006113    1.016015    1.005158    1.012388    1.009184
## [1] "--------------------Model Output--------------------"
## MODEL INFO:
## Observations: 1764
## Dependent Variable: PHLAB
## Type: Mixed effects linear regression
##
## MODEL FIT:
## AIC = 3519.07, BIC = 3606.68
## Pseudo-R² (fixed effects) = 0.48
## Pseudo-R² (total) = 0.54
##
## FIXED EFFECTS:
## ------------------------------------------------------------
##                      Est.   S.E.   t val.      d.f.      p
## ----------------- ------- ------ -------- --------- ------
## (Intercept)          0.07   0.07     1.06     30.16   0.30
## WsArea               0.18   0.03     6.68     15.76   0.00
## pctWetland          -0.10   0.08    -1.16      8.15   0.28
## prec                -0.40   0.05    -7.45     18.77   0.00
## ag.ws                0.16   0.04     3.84      8.43   0.00
## HydrlCondWs          0.05   0.02     3.17   1723.60   0.00
## CaOWs                0.25   0.04     5.86     14.94   0.00
## ------------------------------------------------------------
##
## p values calculated using Satterthwaite d.f.
##
## RANDOM EFFECTS:
## ---------------------------------------
##    Group       Parameter    Std. Dev.
## ------------ ------------- -----------
##   PROVINCE    (Intercept)     0.24
##  PROVINCE.1     WsArea        0.07
##  PROVINCE.2   pctWetland      0.23
##  PROVINCE.3     imp.ws        0.14
##  PROVINCE.4     for.ws        0.13
##  PROVINCE.5      prec         0.17
##  PROVINCE.6      ag.ws        0.08
##  PROVINCE.7      CaOWs        0.14
##   Residual                    0.62
## ---------------------------------------
##
## Grouping variables:
## ----------------------------
```

```
##   Group     # groups   ICC
## ---------- ---------- ------
##  PROVINCE      24      0.09
## ----------------------------
## [1] "--------------------------------------------------------------------------
---------"
## [1] "TSS"
## [1] "--------------------Model Performance--------------------"
## # Indices of model performance
##
## AIC      |      BIC | R2 (cond.) | R2 (marg.) |   ICC |  RMSE | Sigma
## ---------------------------------------------------------------------
## 4210.055 | 4280.708 |      0.258 |      0.173 | 0.104 | 0.785 | 0.799
## [1] "--------------------Variable Inflation Factor--------------------"
##   pctWetland       for.ws        subsz  HydrlCondWs CompStrgthWs
##     1.016399     1.035404     1.025670     1.194088     1.206555
## [1] "--------------------Model Output--------------------"
## MODEL INFO:
## Observations: 1694
## Dependent Variable: TSS
## Type: Mixed effects linear regression
##
## MODEL FIT:
## AIC = 4210.06, BIC = 4280.71
## Pseudo-R² (fixed effects) = 0.17
## Pseudo-R² (total) = 0.26
##
## FIXED EFFECTS:
## ------------------------------------------------------------
##                       Est.   S.E.   t val.      d.f.      p
## ------------------ ------- ------ -------- --------- ------
## (Intercept)          -0.13   0.08    -1.64     19.10   0.12
## pctWetland           -0.13   0.02    -5.23   1192.53   0.00
## for.ws               -0.22   0.05    -4.31     24.52   0.00
## subsz                -0.20   0.02    -8.09   1683.96   0.00
## HydrlCondWs          -0.05   0.02    -2.20   1622.88   0.03
## CompStrgthWs         -0.08   0.03    -2.54   1416.52   0.01
## ------------------------------------------------------------
##
## p values calculated using Satterthwaite d.f.
##
## RANDOM EFFECTS:
## ---------------------------------------
##    Group       Parameter    Std. Dev.
## ------------ ------------- -----------
##   PROVINCE    (Intercept)     0.27
##  PROVINCE.1     WsArea        0.14
##  PROVINCE.2     for.ws        0.15
##  PROVINCE.3      prec         0.19
##  PROVINCE.4   AgKffactWs      0.11
##  PROVINCE.5      BFIWs        0.12
##   Residual                    0.80
## ---------------------------------------
##
## Grouping variables:
## ----------------------------
##   Group     # groups   ICC
```

```
## ---------- ---------- ------
##  PROVINCE      23      0.09
## ----------------------------
## [1] "----------------------------------------------------------------------------
---------"
## [1] "AL"
## [1] "-------------------Model Performance-------------------"
## # Indices of model performance
##
## AIC      |      BIC | R2 (cond.) | R2 (marg.) |   ICC |  RMSE | Sigma
## ---------------------------------------------------------------------
## 3029.153 | 3090.033 |      0.220 |      0.125 | 0.109 | 0.815 | 0.830
## [1] "-------------------Variable Inflation Factor-------------------"
##   pctWetland         prec        ag.ws  HydrlCondWs CompStrgthWs
Al2O3Ws
##     1.006515     1.011137     1.047467     1.217825     1.227604
1.038755
## [1] "-------------------Model Output-------------------"
## MODEL INFO:
## Observations: 1180
## Dependent Variable: AL
## Type: Mixed effects linear regression
##
## MODEL FIT:
## AIC = 3029.15, BIC = 3090.03
## Pseudo-R² (fixed effects) = 0.13
## Pseudo-R² (total) = 0.22
##
## FIXED EFFECTS:
## ----------------------------------------------------------
##                       Est.   S.E.   t val.      d.f.      p
## ------------------ ------- ------ -------- --------- ------
## (Intercept)          -0.21   0.08    -2.73     24.31   0.01
## pctWetland            0.18   0.07     2.71      4.82   0.04
## prec                  0.10   0.04     2.68    400.96   0.01
## ag.ws                -0.13   0.04    -3.51   1019.00   0.00
## HydrlCondWs          -0.08   0.03    -2.72   1158.79   0.01
## CompStrgthWs          0.10   0.04     2.67    954.59   0.01
## Al2O3Ws               0.07   0.03     2.22    708.07   0.03
## ----------------------------------------------------------
##
## p values calculated using Satterthwaite d.f.
##
## RANDOM EFFECTS:
## ------------------------------------------
##    Group       Parameter    Std. Dev.
## ------------ ------------- -----------
##   PROVINCE    (Intercept)     0.29
##  PROVINCE.1     WsArea        0.13
##  PROVINCE.2   pctWetland      0.14
##  PROVINCE.3     imp.ws        0.12
##   Residual                    0.83
## ------------------------------------------
##
## Grouping variables:
## ----------------------------
##   Group     # groups   ICC
```

```
## ---------- ---------- ------
##  PROVINCE      24      0.10
## ----------------------------
## [1] "-----------------------------------------------------------------
---------"
## [1] "CA"
## [1] "-------------------Model Performance-------------------"
## # Indices of model performance
##
## AIC      |      BIC | R2 (cond.) | R2 (marg.) |   ICC |  RMSE | Sigma
## ---------------------------------------------------------------------
## 3160.156 | 3258.945 |      0.641 |      0.572 | 0.162 | 0.533 | 0.546
## [1] "-------------------Variable Inflation Factor--------------------"
##       WsArea   pctWetland       imp.ws       for.ws         prec
subsz
##     1.044462     1.019046     1.046949     1.125512     1.099699
1.028577
## CompStrgthWs        CaOWs
##     1.012002     1.004433
## [1] "-------------------Model Output-------------------"
## MODEL INFO:
## Observations: 1787
## Dependent Variable: CA
## Type: Mixed effects linear regression
##
## MODEL FIT:
## AIC = 3160.16, BIC = 3258.94
## Pseudo-R² (fixed effects) = 0.57
## Pseudo-R² (total) = 0.64
##
## FIXED EFFECTS:
## ----------------------------------------------------------
##                       Est.   S.E.   t val.      d.f.      p
## ------------------ ------- ------ -------- ---------- ------
## (Intercept)           0.02   0.07     0.33     26.16   0.74
## WsArea                0.07   0.02     4.29   1744.78   0.00
## pctWetland           -0.08   0.02    -4.62   1475.39   0.00
## imp.ws                0.16   0.04     4.40     12.65   0.00
## for.ws               -0.16   0.04    -4.11     25.21   0.00
## prec                 -0.35   0.05    -7.08     17.28   0.00
## subsz                -0.05   0.02    -3.21   1757.72   0.00
## CompStrgthWs         -0.15   0.04    -3.51     11.18   0.00
## CaOWs                 0.34   0.04     8.70     14.77   0.00
## ----------------------------------------------------------
##
## p values calculated using Satterthwaite d.f.
##
## RANDOM EFFECTS:
## ---------------------------------------
##    Group       Parameter     Std. Dev.
## ------------ -------------- -----------
##   PROVINCE    (Intercept)      0.24
##  PROVINCE.1      imp.ws        0.10
##  PROVINCE.2      for.ws        0.11
##  PROVINCE.3       prec         0.14
##  PROVINCE.4      ag.ws         0.10
##  PROVINCE.5   CompStrgthWs     0.15
```

```
##  PROVINCE.6      CaOWs         0.13
##  PROVINCE.7      BFIWs         0.18
##   Residual                     0.55
## ---------------------------------------
##
## Grouping variables:
## ----------------------------
##   Group    # groups   ICC
## ---------- ---------- ------
##  PROVINCE     24      0.12
## ----------------------------
## [1] "------------------------------------------------------------------------
---------"
## [1] "ANC"
## [1] "--------------------Model Performance-------------------"
## # Indices of model performance
##
## AIC      |      BIC | R2 (cond.) | R2 (marg.) |   ICC |  RMSE | Sigma
## -------------------------------------------------------------------------
## 2833.163 | 2931.963 |      0.677 |      0.595 | 0.203 | 0.480 | 0.493
## [1] "--------------------Variable Inflation Factor--------------------"
##   pctWetland       imp.ws       for.ws         prec        subsz
ag.ws
##     1.097311     1.122374     1.167808     1.081355     1.024475
1.205686
## CompStrgthWs        CaOWs
##     1.007773     1.004278
## [1] "--------------------Model Output-------------------"
## MODEL INFO:
## Observations: 1788
## Dependent Variable: ANC
## Type: Mixed effects linear regression
##
## MODEL FIT:
## AIC = 2833.16, BIC = 2931.96
## Pseudo-R² (fixed effects) = 0.60
## Pseudo-R² (total) = 0.68
##
## FIXED EFFECTS:
## -----------------------------------------------------------
##                       Est.   S.E.   t val.      d.f.      p
## ------------------ ------- ------ -------- --------- ------
## (Intercept)           0.02   0.07     0.24     26.16   0.81
## pctWetland           -0.11   0.02    -6.51   1660.49   0.00
## imp.ws                0.07   0.02     4.02   1382.76   0.00
## for.ws               -0.16   0.04    -3.70     26.01   0.00
## prec                 -0.34   0.04    -8.17     15.56   0.00
## subsz                -0.05   0.01    -3.03   1744.17   0.00
## ag.ws                 0.06   0.02     2.82   1241.56   0.00
## CompStrgthWs         -0.15   0.05    -2.86     16.57   0.01
## CaOWs                 0.31   0.04     7.26     17.73   0.00
## -----------------------------------------------------------
##
## p values calculated using Satterthwaite d.f.
##
## RANDOM EFFECTS:
## ---------------------------------------
```

```
##    Group        Parameter     Std. Dev.
## ------------ -------------- -----------
##   PROVINCE    (Intercept)      0.25
##  PROVINCE.1      WsArea        0.08
##  PROVINCE.2      for.ws        0.16
##  PROVINCE.3       prec         0.11
##  PROVINCE.4   HydrlCondWs      0.05
##  PROVINCE.5   CompStrgthWs     0.20
##  PROVINCE.6      CaOWs         0.16
##  PROVINCE.7      BFIWs         0.16
##   Residual                     0.49
## ---------------------------------------
##
## Grouping variables:
## ----------------------------
##   Group     # groups   ICC
## ---------- ---------- ------
##  PROVINCE      24      0.14
## ----------------------------
## [1] "---------------------------------------------------------------------------
---------"
## [1] "COLOR"
## [1] "-------------------Model Performance-------------------"
## # Indices of model performance
##
## AIC      |      BIC | R2 (cond.) | R2 (marg.) |   ICC |  RMSE | Sigma
## ---------------------------------------------------------------------
## 4297.162 | 4368.502 |      0.435 |      0.399 | 0.059 | 0.762 | 0.773
## [1] "-------------------Variable Inflation Factor-------------------"
## pctWetland     imp.ws       prec      subsz      ag.ws      BFIWs
##   1.016266   1.049992   1.025353   1.026301   1.074605   1.039613
## [1] "-------------------Model Output-------------------"
## MODEL INFO:
## Observations: 1786
## Dependent Variable: COLOR
## Type: Mixed effects linear regression
##
## MODEL FIT:
## AIC = 4297.16, BIC = 4368.50
## Pseudo-R² (fixed effects) = 0.40
## Pseudo-R² (total) = 0.44
##
## FIXED EFFECTS:
## ----------------------------------------------------------
##                      Est.   S.E.   t val.      d.f.      p
## ----------------- ------- ------ -------- --------- ------
## (Intercept)          0.01   0.06     0.17     23.98   0.87
## pctWetland           0.58   0.08     7.27      8.48   0.00
## imp.ws               0.05   0.02     1.99    749.17   0.05
## prec                -0.15   0.03    -4.75    534.26   0.00
## subsz               -0.09   0.02    -4.20   1757.49   0.00
## ag.ws                0.15   0.04     3.43     73.97   0.00
## BFIWs               -0.09   0.03    -3.66   1137.27   0.00
## ----------------------------------------------------------
##
## p values calculated using Satterthwaite d.f.
##
```

```
## RANDOM EFFECTS:
## ---------------------------------------
##    Group       Parameter     Std. Dev.
## ------------ -------------- -----------
##   PROVINCE    (Intercept)      0.19
##  PROVINCE.1    pctWetland      0.20
##  PROVINCE.2      for.ws        0.14
##  PROVINCE.3   kgNperWsArea     0.20
##  PROVINCE.4   HydrlCondWs      0.12
##   Residual                    0.77
## ---------------------------------------
##
## Grouping variables:
## ----------------------------
##   Group     # groups   ICC
## ---------- ---------- ------
##  PROVINCE      24      0.05
## ----------------------------
## [1] "----------------------------------------------------------------------
---------"
## [1] "COND"
## [1] "-------------------Model Performance-------------------"
## # Indices of model performance
##
## AIC      |      BIC | R2 (cond.) | R2 (marg.) |   ICC |  RMSE | Sigma
## ---------------------------------------------------------------------
## 2734.122 | 2832.678 |      0.693 |      0.665 | 0.085 | 0.478 | 0.489
## [1] "-------------------Variable Inflation Factor-------------------"
##       WsArea   pctWetland       imp.ws       for.ws         prec
subsz
##     1.022092     1.125918     1.053985     1.222673     1.087435
1.041764
## CompStrgthWs         CaOWs        BFIWs
##     1.075898     1.009298     1.123171
## [1] "-------------------Model Output-------------------"
## MODEL INFO:
## Observations: 1764
## Dependent Variable: COND
## Type: Mixed effects linear regression
##
## MODEL FIT:
## AIC = 2734.12, BIC = 2832.68
## Pseudo-R² (fixed effects) = 0.66
## Pseudo-R² (total) = 0.69
##
## FIXED EFFECTS:
## ------------------------------------------------------------
##                        Est.   S.E.   t val.      d.f.      p
## ------------------ ------- ------ -------- --------- ------
## (Intercept)          -0.05   0.05    -1.12     28.06   0.27
## WsArea                0.06   0.02     2.66     15.81   0.02
## pctWetland           -0.08   0.02    -5.26   1551.60   0.00
## imp.ws                0.22   0.03     6.68     16.06   0.00
## for.ws               -0.14   0.02    -5.74    520.23   0.00
## prec                 -0.45   0.05    -8.82     26.07   0.00
## subsz                -0.06   0.01    -3.83   1725.20   0.00
## CompStrgthWs         -0.11   0.02    -5.55   1555.69   0.00
```

```
## CaOWs                 0.20   0.03     6.40     13.72   0.00
## BFIWs                -0.14   0.02    -8.68   1507.51   0.00
## ------------------------------------------------------------
##
## p values calculated using Satterthwaite d.f.
##
## RANDOM EFFECTS:
## --------------------------------------
##    Group       Parameter    Std. Dev.
## ------------ ------------- -----------
##   PROVINCE    (Intercept)     0.15
##  PROVINCE.1     WsArea        0.07
##  PROVINCE.2     imp.ws        0.09
##  PROVINCE.3      prec         0.18
##  PROVINCE.4      ag.ws        0.09
##  PROVINCE.5      CaOWs        0.10
##  PROVINCE.6       SWs         0.30
##   Residual                    0.49
## --------------------------------------
##
## Grouping variables:
## ----------------------------
##   Group     # groups   ICC
## ---------- ---------- ------
##  PROVINCE      24      0.05
## ----------------------------
## [1] "----------------------------------------------------------------------------
---------"
## [1] "DOC"
## [1] "-------------------Model Performance-------------------"
## # Indices of model performance
##
## AIC      |      BIC | R2 (cond.) | R2 (marg.) |   ICC |  RMSE | Sigma
## ---------------------------------------------------------------------
## 3653.812 | 3730.656 |      0.576 |      0.545 | 0.068 | 0.622 | 0.635
## [1] "-------------------Variable Inflation Factor-------------------"
## pctWetland     imp.ws       prec      subsz      BFIWs
##   1.012656   1.012043   1.018887   1.014883   1.010873
## [1] "-------------------Model Output-------------------"
## MODEL INFO:
## Observations: 1788
## Dependent Variable: DOC
## Type: Mixed effects linear regression
##
## MODEL FIT:
## AIC = 3653.81, BIC = 3730.66
## Pseudo-R² (fixed effects) = 0.55
## Pseudo-R² (total) = 0.58
##
## FIXED EFFECTS:
## -----------------------------------------------------------
##                        Est.   S.E.   t val.      d.f.      p
## ----------------- ------- ------ -------- --------- ------
## (Intercept)         -0.05   0.06    -0.81     18.69   0.43
## pctWetland           0.55   0.08     7.09      5.62   0.00
## imp.ws               0.10   0.02     4.85    696.05   0.00
## prec                -0.41   0.06    -6.77     20.91   0.00
```

```
## subsz               -0.13   0.02    -6.84   1765.46   0.00
## BFIWs               -0.26   0.02   -12.30   1279.01   0.00
## -----------------------------------------------------------
##
## p values calculated using Satterthwaite d.f.
##
## RANDOM EFFECTS:
## --------------------------------------
##    Group       Parameter    Std. Dev.
## ------------ ------------- -----------
##   PROVINCE    (Intercept)     0.17
##  PROVINCE.1     WsArea        0.08
##  PROVINCE.2   pctWetland      0.20
##  PROVINCE.3     for.ws        0.16
##  PROVINCE.4      prec         0.21
##  PROVINCE.5      ag.ws        0.25
##  PROVINCE.6      OmWs         0.20
##   Residual                    0.63
## --------------------------------------
##
## Grouping variables:
## ----------------------------
##   Group     # groups   ICC
## ---------- ---------- ------
##  PROVINCE      24      0.04
## ----------------------------
## [1] "-----------------------------------------------------------------------
---------"
## [1] "MG"
## [1] "-------------------Model Performance-------------------"
## # Indices of model performance
##
## AIC      |      BIC | R2 (cond.) | R2 (marg.) |   ICC |  RMSE | Sigma
## ---------------------------------------------------------------------
## 3222.795 | 3327.073 |      0.642 |      0.599 | 0.108 | 0.545 | 0.558
## [1] "-------------------Variable Inflation Factor-------------------"
##       WsArea   pctWetland       imp.ws         prec        subsz
ag.ws
##     1.041010     1.025297     1.027905     1.048175     1.021291
1.039310
## CompStrgthWs        MgOWs        BFIWs
##     1.043087     1.025942     1.039517
## [1] "-------------------Model Output-------------------"
## MODEL INFO:
## Observations: 1787
## Dependent Variable: MG
## Type: Mixed effects linear regression
##
## MODEL FIT:
## AIC = 3222.80, BIC = 3327.07
## Pseudo-R² (fixed effects) = 0.60
## Pseudo-R² (total) = 0.64
##
## FIXED EFFECTS:
## -------------------------------------------------------------
##                         Est.   S.E.   t val.      d.f.      p
## ------------------ ------- ------ -------- --------- ------
```

```
## (Intercept)          -0.06   0.06    -1.04     29.23   0.31
## WsArea                0.04   0.02     2.38   1751.51   0.02
## pctWetland           -0.05   0.02    -2.78   1332.99   0.01
## imp.ws                0.13   0.03     4.41     10.81   0.00
## prec                 -0.53   0.06    -9.32     21.85   0.00
## subsz                -0.06   0.03    -2.44     15.49   0.03
## ag.ws                 0.18   0.06     2.95     11.50   0.01
## CompStrgthWs         -0.13   0.02    -5.40   1425.09   0.00
## MgOWs                 0.23   0.03     7.27     20.00   0.00
## BFIWs                -0.14   0.03    -4.58      7.41   0.00
## ------------------------------------------------------------
##
## p values calculated using Satterthwaite d.f.
##
## RANDOM EFFECTS:
## ---------------------------------------
##    Group       Parameter    Std. Dev.
## ------------ ------------- -----------
##   PROVINCE    (Intercept)     0.19
##  PROVINCE.1     imp.ws        0.07
##  PROVINCE.2     for.ws        0.13
##  PROVINCE.3      prec         0.20
##  PROVINCE.4      subsz        0.07
##  PROVINCE.5      ag.ws        0.19
##  PROVINCE.6      MgOWs        0.09
##  PROVINCE.7      BFIWs        0.07
##   Residual                    0.56
## ---------------------------------------
##
## Grouping variables:
## ----------------------------
##   Group     # groups   ICC
## ---------- ---------- ------
##  PROVINCE      24      0.08
## ----------------------------
## [1] "----------------------------------------------------------------------
---------"
## [1] "NO3"
## [1] "-------------------Model Performance-------------------"
## # Indices of model performance
##
## AIC      |      BIC | R2 (cond.) | R2 (marg.) |   ICC |  RMSE | Sigma
## ---------------------------------------------------------------------
## 3060.858 | 3128.444 |      0.386 |      0.386 | 0.000 | 0.711 | 0.722
## [1] "-------------------Variable Inflation Factor-------------------"
##         pctWetland             imp.ws       kgNperWsArea
BFIWs
##           1.104095           1.007025           1.005238
1.094124
## kgNperWsArea:BFIWs
##           1.017566
## [1] "-------------------Model Output-------------------"
## MODEL INFO:
## Observations: 1338
## Dependent Variable: NO3
## Type: Mixed effects linear regression
##
```

```
## MODEL FIT:
## AIC = 3060.86, BIC = 3128.44
##
## FIXED EFFECTS:
## ---------------------------------------------------------------
##                              Est.   S.E.   t val.     d.f.      p
## ------------------------- ------- ------ -------- -------- ------
## (Intercept)                 -0.17   0.05    -3.54   201.28   0.00
## pctWetland                  -0.09   0.03    -3.35   461.98   0.00
## imp.ws                       0.16   0.03     6.15   356.06   0.00
## kgNperWsArea                 0.52   0.08     6.50     6.31   0.00
## BFIWs                        0.10   0.03     3.28   239.49   0.00
## kgNperWsArea:BFIWs           0.15   0.05     3.21     6.64   0.02
## ---------------------------------------------------------------
##
## p values calculated using Satterthwaite d.f.
##
## RANDOM EFFECTS:
## --------------------------------------------------
##       Group             Parameter        Std. Dev.
## ------------------ -------------------- -----------
##   AGGR_ECO9_2015          for.ws           0.10
##  AGGR_ECO9_2015.1          prec            0.26
##  AGGR_ECO9_2015.2        pt.N.aw           0.21
##  AGGR_ECO9_2015.3          NWs             0.19
##  AGGR_ECO9_2015.4      kgNperWsArea        0.21
##  AGGR_ECO9_2015.5   kgNperWsArea:BFIWs     0.11
##      Residual                              0.72
## --------------------------------------------------
##
## Grouping variables:
## ---------------------------------
##      Group        # groups   ICC
## ---------------- ---------- ------
##  AGGR_ECO9_2015      9       0.01
## ---------------------------------
## [1] "------------------------------------------------------------------------
---------"
## [1] "TURB"
## [1] "-------------------Model Performance-------------------"
## # Indices of model performance
##
## AIC      |      BIC | R2 (cond.) | R2 (marg.) |   ICC |  RMSE | Sigma
## ---------------------------------------------------------------------
## 4236.789 | 4313.444 |      0.323 |      0.256 | 0.090 | 0.753 | 0.767
## [1] "-------------------Variable Inflation Factor-------------------"
##   pctWetland       for.ws         prec        subsz kgNperWsArea
ClayWs
##     1.099594     1.204604     1.139598     1.027133     1.207957
1.052349
## [1] "-------------------Model Output-------------------"
## MODEL INFO:
## Observations: 1764
## Dependent Variable: TURB
## Type: Mixed effects linear regression
##
## MODEL FIT:
```

```
## AIC = 4236.79, BIC = 4313.44
## Pseudo-R² (fixed effects) = 0.26
## Pseudo-R² (total) = 0.32
##
## FIXED EFFECTS:
## ------------------------------------------------------------
##                        Est.   S.E.   t val.      d.f.      p
## ------------------ ------- ------ -------- --------- ------
## (Intercept)          -0.08   0.07    -1.17     14.83   0.26
## pctWetland           -0.04   0.02    -1.69   1403.35   0.09
## for.ws               -0.10   0.04    -2.20     39.82   0.03
## prec                 -0.19   0.06    -3.35     15.49   0.00
## subsz                -0.24   0.04    -6.50     13.82   0.00
## kgNperWsArea          0.09   0.03     2.71    698.97   0.01
## ClayWs                0.12   0.03     4.44   1293.08   0.00
## ------------------------------------------------------------
##
## p values calculated using Satterthwaite d.f.
##
## RANDOM EFFECTS:
## ----------------------------------------
##    Group       Parameter    Std. Dev.
## ------------ ------------- -----------
##   PROVINCE    (Intercept)     0.24
##  PROVINCE.1     WsArea        0.11
##  PROVINCE.2     for.ws        0.12
##  PROVINCE.3      prec         0.14
##  PROVINCE.4      subsz        0.12
##  PROVINCE.5      BFIWs        0.15
##   Residual                    0.77
## ----------------------------------------
##
## Grouping variables:
## ----------------------------
##   Group     # groups   ICC
## ---------- ---------- ------
##  PROVINCE      24      0.08
## ----------------------------
```
